# Investigative research for occurrences of hydrogen, helium, methane and carbon dioxide in West Macedonia: linking geological reservoirs to subsurface gas generation and migration

**DOI:** 10.12688/openreseurope.21180.1

**Published:** 2025-09-05

**Authors:** Pavlos Tyrologou, Nikolaos Koukouzas, Nazaré Couto, Christos L. Stergiou, Júlio Carneiro

**Affiliations:** 1 Chemical Process and Energy Resources Institute (CPERI), Centre for Research and Technology Hellas (CERTH), Marousi, Egialias 52, 151 25,, Greece; 2 Center for Environmental and Sustainability Research & CHANGE - Global Change and Sustainability Institute, NOVA School of Science and Technology, NOVA University, Lisbon, Campus de Caparica, 2829-516 Caparica, Portugal; 3 ICT/IIFA, Geosciences Department, Universidade de Évora, Évora, R. Romão Ramalho, 7000-671, Portugal

**Keywords:** helium, methane, natural hydrogen, geological storage, gas migration, isotope, geological reservoirs

## Abstract

**Background:**

Climate change, the need for energy optimisation and higher efficiency have led to the adoption of the Paris Agreement as a response to the urge for action. The European Union has translated the aforementioned into an action framework via the Green Deal and the EU taxonomy regulation. These have initiated a series of research actions under the EU Horizon programme. Part of this research is based on carbon dioxide capture and geological storage, such as the Pilot Strategy, and hydrogen storage, such as the HyStorIES, both Horizon 2020 project. A focused hydrogeochemical survey as part of a larger mapping survey was conducted in West Macedonia to identify a potentially suitable location for gas reservoirs, gas sources and gas migration routes based on previous research. Gases investigated were hydrogen, helium, methane and carbon dioxide. The study involved isotopes to identify the source of gases and thus provide clues for generation and migration routes.

**Methods:**

The investigation presented in this study deployed sequential spring and borehole water sampling for geochemical analysis of trace elements and gas analysis for hydrogen, helium, methane and carbon dioxide to identify and characterise gaseous geological reservoirs. The investigation extended into isotope studies for d ^13^C _TDC_, d ^13^C _CH4_, dD _CH4_, δD _H20_, δ ^18^Ο _Η2Ο_.

**Results:**

The analysis provided evidence for the existence of helium, biogenic methane, carbon dioxide and traces of hydrogen that need to be further investigated for validation and better understanding of the gas generation and migration routes.

**Conclusions:**

The data suggests the existence of helium, methane, carbon dioxide and validated trace concentrations of hydrogen from previous studies in the wider area. Isotopic analysis provides strong evidence for biotic generation of methane, whereas helium comes from a deeper source. This preliminary investigation indicates the existence of multiple gas generation and migration mechanisms and paves the way for further research.

## Plain language summary

As our humanity evolves, we realise that our current way of converting energy is not sustainable, it creates geopolitical tensions, competition for resources and results in climate change. The future of the current generation and future ones is at stake. We have already experienced the results of climate change and its direct impacts on the food production chain, as well as water scarcity, manifesting themselves in cost and price increases in developed countries, whereas in underdeveloped and developing countries, the situation is difficult to estimate, but it is even worse. Our research focuses on alternative ways of managing energy and carbon dioxide storage to battle climate change for a just society. In addition, we investigate alternative localised natural sources of energy such as natural hydrogen and methane to support energy independence for prosperous societies.

## Introduction

The relatively recent political change on tackling the climate change issue has been expressed with the Paris Agreement ^1^ on a global scale. The Green Deal ^2^ in the European Union has signalled the way forward for the energy transition and decarbonisation of industry. The urgent need to address greenhouse gas emissions from fossil fuel combustion has led to a major transition toward alternative energy sources ^3^. Carbon capture and storage ^4, 5^ technology has provided an intermediate solution to keep business as usual before more holistic approaches are employed that will allow for a smoother technical and social transition everywhere. Renewable energy, including wind turbines and solar panels, can be part of the solution given that their cost is highly reduced, albeit the issue of the instability they create to the grid they tend to produce during surplus energy not absorbed by demand.

To store the excess energy and stabilise the grid, various feasible solutions have been presented. Some include pumped storage hydropower, compressed air storage, underground carbon dioxide circulation as a medium to harvest thermal energy and convert it again to electricity ^6^, compressed air storage and green hydrogen production produced by hydrolysis that can be temporarily stored in lined rock caverns, salt cavities or saline aquifers and depleted hydrocarbons fields ^7^. Underground hydrogen storage and geological carbon storage have been developed almost in tandem, and the latter has drawn on substantial technical knowledge from the former. Lately, hydrogen and its potential use as a fuel alternative that can stabilise the grid and decarbonise the industry have received a lot of attention. One of the main issues with green hydrogen is that it is a relatively expensive technology to implement, which has an impact on developing countries that may not be able to adopt the technology, despite recognising its importance. As the scientific society strives to find solutions to advance the democratisation of energy and address climate change, the aforementioned technologies have serendipitously led to discoveries and the conceptualisation of natural hydrogen resources. In a similar fashion, work on Pilot Strategy, an EU Horizon2020-funded project related to Carbon Geological Storage, has stumbled upon what appears to be natural hydrogen and helium gas emission during field geological investigation to identify suitable carbon dioxide reservoir rock storage in West Macedonia, Greece. This is the subject of the present scientific publication, which is elaborated in the subsequent sections and investigates the soundness of the scientific hypothesis of natural hydrogen occurrence in West Macedonia.

## Current state of the art of natural hydrogen

The concept of natural hydrogen, although not new, still remains in its infancy with only a handful of researchers together and some pioneer industries actively pursuing the subject to identify large and constant sources that will be able to support a reliable energy transition without leaving anyone behind ^8– 12^.

Based on stochastic modelling earth is estimated to be capable of producing some 5.6 × 10 ^6^ Mt of hydrogen. While most of it is unrecoverable because its accumulation is too deep, too small in volume, dispersed, or too far away offshore. Still, if a small portion of this, such as 10 ^5^ Mt, can be recovered, it would be able to supply the global needs for 200 years with net-zero carbon emissions ^13^. This will provide enough time to achieve nuclear fusion ^14^ and make the next revolutionary step in human civilisation history based on the Kardashev scale ^15^.

Promising results for natural hydrogen have already been provided in Mali ^16^, in Namibia ^17^, in Colombia ^18^, in Australia ^19^, and in the Balkans in Albania and Kosovo ^20^. Daskalopoulou *et al.*, 2018 ^21^, have also reported traces of natural hydrogen in West Macedonia together with helium and methane. Natural hydrogen mechanisms are still under development, but in general include:

1) Contact of water with reducing agents in the mantle ^22– 24^.
2) Reaction of water with ultrabasic rocks, serpentinization ^25^- type reactions involving the reduction of water by iron-rich minerals have generally been regarded as requiring high temperatures (>~200°C) ^26^, although this has been recently challenged with observation at lower temperature conditions (<200°C) ^27, 28^.
3) Reduction of water by iron-rich minerals in banded iron formations ^29, 30^ or biotite-rich granites ^31^.
4) High–thermal maturity organic-rich rocks ^32, 33^.
5) Decomposition of organic matter ^22^ of NH _3 _to N and H _2 _at high temperature (up to 600°C).
6) Natural electrolysis of water ^34^.
7) Natural radiolysis of water ^19, 22^.
8) Mantle degassing ^22, 35^.
9) Biological activity ^25^ via microbes in soils and coal beds.
10) Cataclasis ^22, 23^ during earthquakes.

Hydrogen is highly reactive and susceptible to redox conditions, so preservation throughout migration is a risk ^13^. In addition, microbial communities are capable of utilising and producing hydrogen that exists in the subsurface ^36^. Thus, understanding the mechanisms of hydrogen generation, residence time in reservoirs, biotic and abiotic loss are essential for guiding its exploration and sustainable production. This is also making sure that resource exploitation approaches of the past, whose activities harmed the environment, its ecosystem services, and human health, will not be repeated. On the same note, to reach the carbon net-zero targets, deploying natural hydrogen in the EU will be based on investment and development of exploration and production strategies ^13^ that will satisfy the taxonomy regulation objectives ^37– 41^. Exploration itself at this current stage is based on research and development of natural hydrogen conceptual geological models. The latter will allow for global investigation of related geological analogues as a prospective place for exploitation.

## Previous research related to West Macedonia and neighbouring areas

The PilotSTRATEGY project, funded by the Horizon2020 programme, investigates geological CO _2_ storage sites in industrial regions of Southern and Eastern Europe to support the development of large-scale carbon capture and storage (CCS). It is the successor of the StrategyCCUS project, also funded by the Horizon 2020 programme and consequently builds upon the research funding of its predecessor ^5^. In Greece, West Macedonia, PilotSTRATEGY focuses on the deep saline aquifers, porous rock formations filled with brine several kilometres below ground, which promise a large capacity for storing CO _2_ captured from industrial clusters. The project investigates the geomechanical characteristics and the geological storage capacity of the Mesohellenic Basin ^42^. However, during the investigation, an additional research area related to hydrogen occurrences was identified. To better understand this, studies from geologically related areas are examined to provide insight into the mechanisms of hydrogen generation and migration. Where possible, geological analogues will be sought out to draw similarities and conclusions for further work and exploration.

Research conducted by Levy *et al.*, 2023 ^20^ has identified hot spots of hydrogen occurrences in Albania and Kosova. The study comprised the collection of water samples from natural springs that were analysed against C and H isotopes of CH _4 _and H _2_ when that was feasible. Four springs out of 21 sites provided strong evidence for H _2_ occurrence. The recent finding in Albania, given the geological proximity and affinity to a certain extent, suggests that similar results may appear in Greece in the Mesohellenic basin.

The Mirdita ophiolite in Albania presents interest and a potential analogue as it is located in the Albanides mountain belt that is a segment of the Dinarides–Albanides–Hellenides orogen. It is bounded on the east by the Pelagonian block and on the west by the Krasta, Cukali, and Kruja zones. The latter is well-known for its geothermal potential due to thrust faults that allow hot fluids percolation. The western massifs of the Mirdita ophiolite are mantle domes of harzburgite (peridotite composed of olivine and orthopyroxene and small amounts of chromium-rich spinel) capped by mylonitic plagioclase-amphibole peridotite of 400 m thickness. Harzburgite peridotite is composed of olivine and orthopyroxene and small quantities of chromium-rich spinel formed in the upper mantle ^43^. The eastern massifs of the Mirdita ophiolite are mainly harzburgite. The northeastern part of the ophiolite complex can reach 14 km in thickness, whereas the west and southeast are estimated to be 2 km thick ^20^. In Kosova, ophiolites are present in most of the country, while of interest to this work are the ophiolites of the West Vardar zone, part of the Dinarides, in the continuity of Mirdita ophiolite. Of similar interest is the central ophiolite of Kosova that has hydrothermally metamorphosed, leading to the economic precipitation of magnesite ^20^.

In Central Greece, Tsikouras *et al*. (2013) ^44^ and Etiope *et al.* (2013) ^45^ investigated 21 ophiolitic springs in the serpentinised Othrys ophiolite. Four hyperalkaline springs (pH 10.7–11.3) with calcium-rich (Ca–OH) waters indicated the presence of methane and hydrogen. These springs, one in Archani and three in Ekkara, are located along creeks and tectonically related to faults that are directly linked to the main regional active fault system. An estimation revealed that collectively the springs produce some 100 kg/year of methane ^45^. In contrast, methane flux from the soil surrounding the Ekkara well in a 300 m ^2^ area gives an estimated methane production of 70 kg per year ^45^. The remaining 17 springs, which had pH<8.7 values, did not show detectable CH _4_ or H _2_.

The Othrys ophiolite complex is located in central Greece, specifically within the Dinaric–Hellenic ophiolite belt and lies uncomfortably into the Pelagonian carbonate platform. It represents an obduction event where an oceanic lithosphere has been thrust onto continental crust. The sampling location area was within the Archani and Ekkara area, at the southwestern part of the Othrys ophiolite, where outcrops of medium- to coarse-grained serpentinised harzburgite with lesser plagioclase-bearing lherzolite are present. These are complemented by dunite forming thin layers, a few cm to a few meters thick, intercalated in the harzburgite. A remnant doleritic sheeted-dyke complex is tectonically overlain by peridotites. Whitish rodingite dykes are found in serpentinised harzburgites very close to the Archani spring ^28^. The peridotites are in tectonic contact with an ophiolitic mélange, which in turn tectonically overlies a Late Cretaceous-Tertiary flysch. An active fault zone, along the Leontari-Anavra line, is found north of Ekkara, at the south border of the Thessaly Plain ^28, 44, 45^.

The water spring isotope chemical analysis of CH _4_ has revealed that the origin of the methane is not biotic. Thus, in hyperalkaline high silica fluids can positively influence the production of H _2_ whereas low silica fluids may result in low H _2_ production. Othrys springs albeit hydrogen positive, still are releasing low amounts of H _2_ compared to other similar places ^28, 44, 45^. This can be attributed to:

(i) normal hydrogen generation due to interaction with low-silica fluids alone, followed by consumption through CO _2_ hydrogenation and/or loss to the surface as water degasses. Note that H _2_ solubility is much less compared to CH _4_, by about an order of magnitude.
(ii) direct methanation processes involving both low- and high-silica fluids ^13, 44, 45^. High-silica activity promotes the formation of talc or other silica-rich phases, which consumes Mg and reduces the production of brucite (Mg(OH) _2_, thereby lowering H _2_ generation.
(iii) Microbial activity that consumes hydrogen.
(iv) Τemperature decrease or modiﬁcations in water–rock ratios ^45^

Daskalopoulou *et al.*, 2018 ^21^, in their study, identified in West Macedonia and particularly in Katakali and Mesohori, traces of helium and hydrogen with significant values of methane. Katakali provided 4 ppm He, 11 ppm H _2_ and 880.000 ppm CH _4_. The Mesohori sample registered 4 ppm He, 1.5 ppm H _2_ and 479 ppm CH _4_. These values albeit low are indicative and worth investigating to understand the underlying fluid generation and migration.

## Derived geological conceptual model to be used as an analogue

Areas with potential for natural hydrogen exploration/exploitation require a source of sufficient hydrogen generation, a mechanism for vertical and lateral fluid migration, porous rock reservoirs for storage, and low permeability rocks (seals) to prevent leakage and allow for economically viable reserves. An additional component that relates to preservation needs to be considered due to hydrogen’s high reactivity and microbial consumption. Natural hydrogen is generated in high depths, while it is consumed in shallow ones ^46^.

The main takeaway from the previous sections is that the detection of abiotic methane, brucite and carbonates is a useful indicator for the presence of natural hydrogen ^17, 47^. These indicators may be found together or individually in the field with closely related ultrabasic rocks. Helium is also generated by decay of radiogenic nuclides in similar rocks and can be related to natural hydrogen ^46^. The presence of talc indicates low chances of hydrogen being freely available. In hyperalkaline systems of Ca-OH, high-silica fluids decrease H _2_ production, whereas low-silica fluids enhance H _2_ generation. Evidence of low silica activity can be indicated by magnetite abundance and the formation of garnet ^45^. Hyperalkaline springs found in ophiolites are related to serpentinization ^20^ via the following general chemical Equation 1:


$$\text{F}{\text{e}}^{2+}+{\text{H}}_{2}\text{O}\overset{\text{yields}}{\to }\text{F}{\text{e}}^{3}+\frac{1}{2}{\text{H}}_{2}+\text{O}{\text{H}}^{-}\;(Equation 1)$$

In terms of the geological setting of interest, ophiolites are thrusted and overlain by carbonate rocks, which can provide ample quantities of CO _2,_ leading to the following very slow reaction ( Equation 2) but nonetheless important over geological time:


$$\text{C}{\text{O}}_{2}+4{\text{H}}_{2}=\text{C}{\text{H}}_{4}+2{\text{H}}_{2}\;\mathrm{with}\;\text{T}<100{}^{\circ}\text{C}\;{\left(\text{Sabatier}\;\text{reaction}\right)}^{28}\;(Equation 2)$$

whereas thrust and other related faults allow for fluid percolation from deep aquifers below or from rainwater above to enable the above reactions to take place. Reaction described in Equation 2 can be catalysed by magnetite (Fe3O4), chromite (FeCr2O4) and awaruite (Ni3Fe), at temperatures above 200 °C ^45, 48^.

## Geological setting for West Macedonia

**Figure 1.**
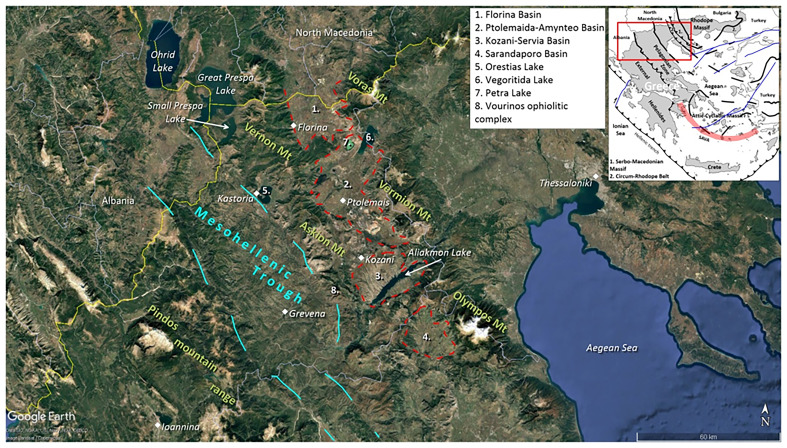
Satellite imagery showing the location of the Mesohellenic Trough, SAVA = South Aegean Volcanic Arc, NAFZ = North Anatolian Fault Zone, modified after Stergiou *et al.* 2025 ^50^ and provided under licence: CC-BY 4.0.

The Mesohellenic Trough (MHT), or Mesohellenic Basin (MHB), is a late-orogenic, molassic-type basin that developed from the Middle Eocene to Middle Miocene in northwestern Greece and southern Albania ^49^. It is presented as a favourable area with a potential for hydrogen accumulations associated with the long-distance lateral flow migration that commonly occurs in sedimentary basins. Long-distance lateral migration is defined as migration across map distances of up to 250 km ^46^. Figure 1 depicts the location of the Mesohellenic Trough (cyan-dashed lines) and of the Florina, Ptolemais-Amyntaio, Kozani-Servia, Sarandaporos Basins (red-dashed lines) across northern Greece. Major mountains and lakes are highlighted. The inset map depicts the study area (red rectangle) with respect to the geotectonic zones and the major tectonic features of the Hellenic Orogen and the Aegean domain ^50^.

The MHT extends over 200 km in a NNW-SSE direction and is 20 to 40 km wide, lying along the suture between the Apulian plate and the Pelagonian nappe pile, thus marking the boundary between the Internal and External Hellenides ^51– 53^. The basin formed atop westward-emplaced Tethyan ophiolites following their final emplacement between Middle and Late Eocene during the Alpine orogeny, with subsidence driven by crustal loading and flexure ^49, 54^. The southern part of the basin is structurally divided by the Theopetra-Theotokos Structure, a horst or faulted anticline, which separates the basin into western and eastern sub-basins. These are represented by the Pentalofon and Tsotyli–Ondria formations, respectively.

The stratigraphy of the MHT is subdivided into seven major lithostratigraphic units ^55– 58^. These include: 1) the Middle-Upper Eocene Krania Formation (Fm) incorporating conglomerates and olistoliths (lower sequence) and turbiditic beds (upper sequence) with a thickness of 1500 m, 2) the Oligocene Eptachorion Fm with silty marls and very-fine sandstone beds overlying thick conglomerates (~1000 m in thickness), 3) the Upper Oligocene-Lower Miocene Taliaros/Tsarnos Fm and the equivalent Pentalofos Fm, showing sequences from sandstones to conglomerates (~2500 m in thickness), 4) the Lower to Middle Miocene Tsotyli Fm with marl-sandstone alternations in the north and conglomerates in the south (~600 m in thickness), and 5) the Ondria Fm and the equivalent Orlias Fm (Lower to Middle Miocene) comprising fossiliferous limestones, marls, and sandstones (≥350 m in thickness). These ΜΗΤ thick sedimentary strata, as a geological reservoir, offer the benefit of (1) sufficient burial to compact rocks to low porosity and (2) a higher likelihood of one or more potential sealing formations throughout the stratigraphic sequence ^46^. Tyrologou *et al.* 2023 have confirmed with field surveys and laboratory investigation the existence of favourable rock sealing conditions related to the Pentalofos, Eptachori and Tsotyli sedimentary formations ^42^ due to their low porosity and permeability values.

Major tectonic unconformities define the bases of the Krania, Eptachorion, and Tsotyli Formations. Lithologies within these formations include conglomerates of fan-delta and alluvial fan origin, turbiditic sandstones and shales, as well as deltaic, floodplain, and shelf sandstones and siltstones ^55– 57^. These sediments show a general coarsening trend from north to south and were deposited during a progressive shallowing of the basin, with a maximum sedimentary thickness reaching 4.5 km near Grevena ^51, 59^. These sedimentary facies hold potential as porous media capable of economically accumulating hydrogen while being protected from overlain low permeability rocks described previously. Upper Miocene unconformably overlies the molassic sequence to Quaternary deposits.

**Figure 2.**
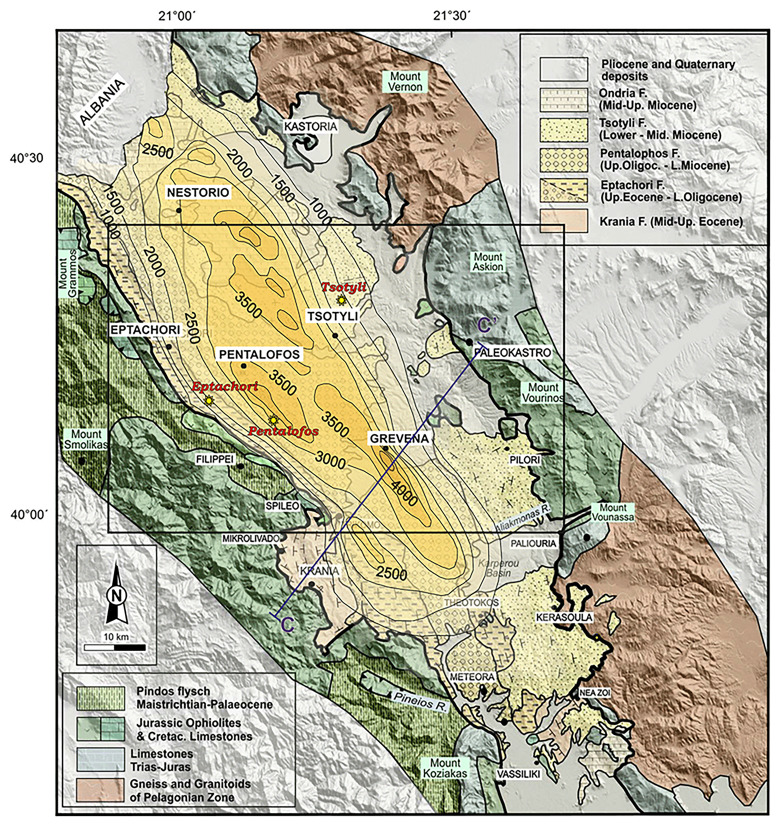
Modified Geological map and isodepths of the basement rocks of the Mesohellenic Basin ^42^, licence: CC-BY 4.0.

**Figure 3.**
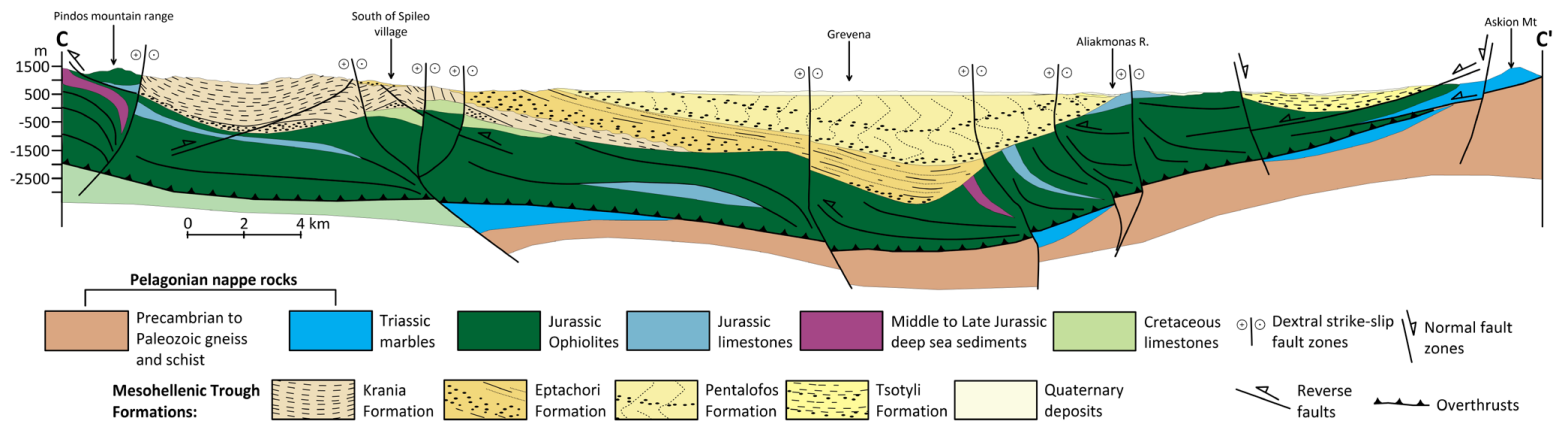
Modified geological cross-section of Mesohellenic Basin ^42^, licence: CC-BY 4.0.

The MHT depicted in Figure 2 is bounded to the west by the Pindos mountain range, comprising Triassic to Jurassic ophiolites, mélange units, Cretaceous limestones, and Maastrichtian to Palaeocene flysch deposits ^49^. The eastern boundary is defined by the Pelagonian nappe rocks exposed at the Vernon and Askion mountains. They include Precambrian to Palaeozoic metamorphic gneiss and schist, Permo-Triassic rift-related volcanosedimentary successions, and Cretaceous carbonates. Thrusted ophiolitic units are also found, with the Vourinos ophiolitic complex being the most profound one ^60^. The MHT has asymmetrical synclinal geometry, with sharp eastward dips at the western boundary and gradual westward dips towards the eastern margin. This asymmetry is ascribed to a combination of depositional tilting and post-depositional tectonic deformation ^53^. The Theopetra-Theotokos Structure in the southern portion of the basin exposes the basement ophiolitic and carbonate units and runs almost parallel to the main basin axis ^53, 58^. A complex tectonic development influenced by both brittle and semi-ductile deformation occurred in the basin. The final emplacement of the Pindos ophiolites was associated with strike-slip faulting and NE-verging thrusting during the first tectonic phase (T1, Middle to Late Eocene), which was characterised by a transpressional regime with NE-SW maximum compressive stress. Along NW-SE to NNW-SSE faults, dextral strike-slip tectonics dominated the subsequent phase (T2, Early to Late Oligocene), allowing for lateral basin displacements and subsidence ( Figure 3) ^61^. A shift to extensional tectonics is reflected in later periods (T3 to T5, Miocene to Quaternary), when shifting fault patterns govern sedimentation and depocenter migration. Recent seismicity (e.g. the 1995 Grevena-Kozani earthquake sequence ^62, 63^; in the Grevena-Kozani region supports the ongoing N-S extension, which suggests ongoing reactivation of inherited structures within the MHT.

The Jurassic oceanic lithosphere was obducted during the closing of the Mesozoic Tethys Ocean, forming the Vourinos and Pindos ophiolites that are exposed along the edges of the MHT ^52, 64^. The Vourinos ophiolite is a well-preserved Penrose-type section made up of pillow lavas, sheeted dikes, a thick ultramafic-mafic cumulate sequence, and mantle peridotites. Hydrothermal activity during seafloor spreading is indicated by features including epidotized dikes, sulfide mineralisation, and jaspers ^65^. In contrast, the Pindos ophiolite to the west is made up of a number of petrologically overlapping, tectonically imbricated nappes that have a total reconstructed thickness reaching 4 km. The upper plutonic suite, which includes dunite, troctolite, and gabbro, and the lower suite, which includes dunite, lherzolite, wehrlite, and pyroxenite, make up its cumulate section, which is less than one kilometre thick and extends from a transitional Moho to the base of the sheeted dike complex ^60^. Large sheeted dikes and pillow lavas that have been preserved in tectonic windows and nappe sequences sit on top of this structure. Despite differences in structure and thickness, both ophiolites are classified as supra-subduction zone ophiolites with island arc tholeiitic and boninitic affinities ^60^. A continuous magnetic anomaly beneath the Mesohellenic Trough suggests a common ophiolitic root, supporting a shared tectonic origin ^51, 65^.

Ophiolites are abundant in the area, offering promising potential as a source rock for natural hydrogen through serpentinization. The underlying Triassic and Jurassic limestones can contribute significant quantities of CO _2_, mobilised by migratory fluids that percolate vertically and laterally through the rock mass as a result of the prevailing tectonic setting and associated strain.

**Figure 4.**
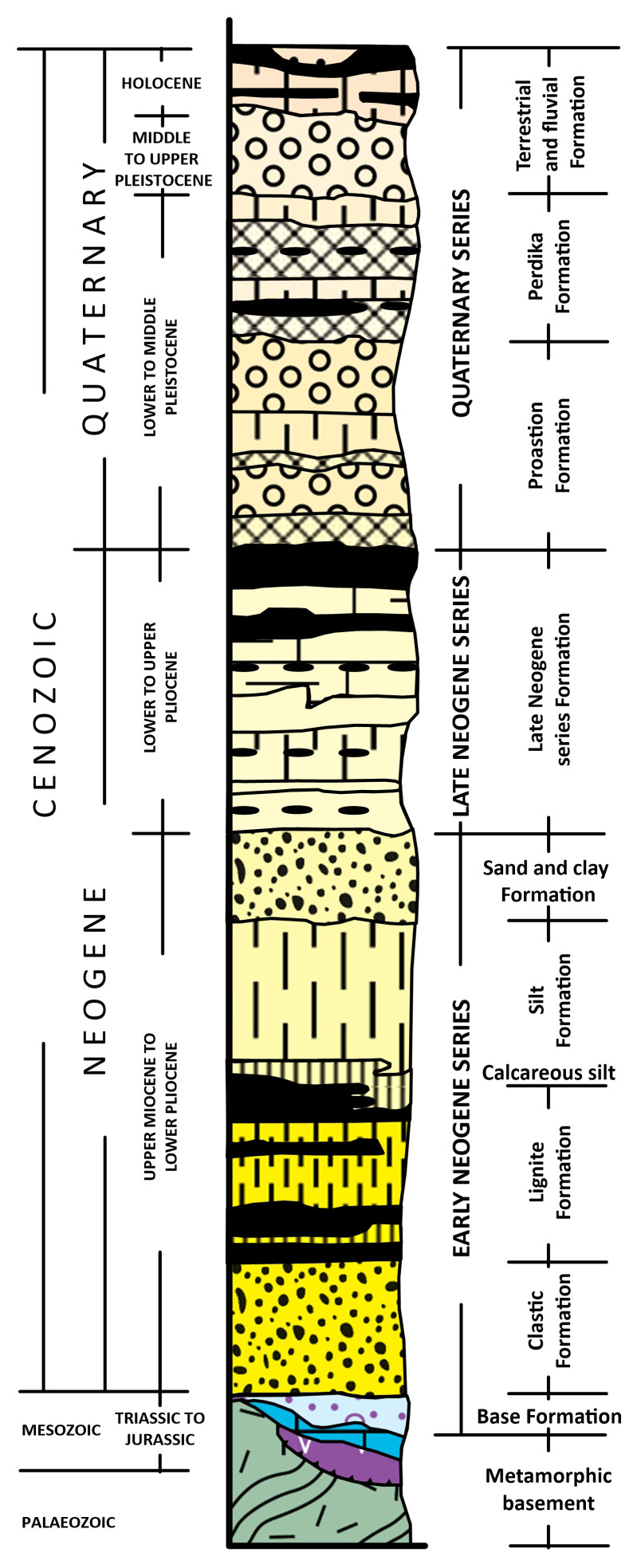
Synthesized stratigraphic column of the Sarantaporos, Ptolemaida-Amynteo -Florina basins, licence: CC-BY 4.0.

The Florina Basin is part of a large NW-SE trending graben system in NW Macedonia, Greece, that formed during the Lower Miocene following the Alpine Orogeny. This graben system (~150 km in length) contains the Florina, Ptolemaida-Amynteo, Kozani-Servia, and Sarandaporo Basins ^66^. The basins were initially formed under a NE-SW extensional regime during the Late Miocene, which was succeeded by an Early Pleistocene NW-SE extension. This resulted in the fragmentation of the originally continuous Florina-Ptolemaida-Servia Basin into three sub-basins. Depicted in the synthesised related stratigraphic column in Figure 4, the basement rocks of the basin system include mainly Triassic to Jurassic crystalline limestones, marbles and dolomites, as well as Palaeozoic to Mesozoic schists, phyllites, gneisses, and granites of the Pelagonian Zone ^66^. Locally, ophiolitic rocks with serpentinites and serpentinized peridotites occur ^62^. As previously discussed, ophiolites present the potential to generate natural hydrogen and can be considered as source rock within the Florina Basin.

Between Late Miocene and Early Pliocene, these basins began to fill with continental, fluvial, lacustrine, and marshy deposits. At the base lies the Base Formation, composed of red to reddish-brown breccias, conglomerates, sands, clays, and mud, varying in thickness and reflecting proximal alluvial-fan and talus origins. The overlying Clastic Formation includes green to green-grey fluvial sands, clays, hard sandstones, and conglomerates, representing a mature fluvial system. Above, the Lignite Formation is characterised by green-grey sands, clays, silts, plant remains, xylite, and beds of xylitic lignite, and hosts the most important lignite seams of the basin system (e.g. Florina, Komnina ^66^). The higher Silt Formation follows, dominated by silts with interbedded sands, clays, and calcareous silts containing organic remains and vivianite. In areas like eastern Florina, a Sand-clay Formation with green sands and clays replaces the fine lacustrine silts ^66^. The Late Neogene Series Formation consists of light grey to grey-green marls, clays, sands, marly limestones, and earthy lignites, containing rich fossil assemblages and significant lignite seams (e.g., Ptolemaida-Amynteo). During the Early–Middle Pleistocene, the Proastion Formation was deposited, comprising fluvial-torrential conglomerates with cross-bedding and minor lignite beds. Sedimentation continued with the development of the Perdika Formation, containing sands, clays, marls, and thin, non-economic lignite beds ^66^. Finally, a Middle Pleistocene Terrestrial Formation marks the latest phase, with conglomerates, red loam, clays, and sands, especially thick in Sarandaporo and parts of Florina basins.

Particularly, in the Florina Basin, these deposits also include lignite-bearing formations rich in xylitic-type lignite, especially along its eastern margins, which can generate natural hydrogen ^22, 67^. During the Late Pliocene, a widespread lacustrine-marshland system was developed and associated sediments were deposited. Marshy and riverine sediments and lignite were deposited during the Lower Pleistocene, followed by fluvial-terrestrial deposition during the Middle Pleistocene. Peat deposition in the Vegoritida and Zazari lakes is indicative of Holocene sedimentation ^66^.

The sedimentary fill of the Florina Basin reaches a total thickness of about 560 m, which is comparable to the 600 m found in the Ptolemais Basin. This suggests that the two sub-basins had a similar geological history up until the early Pleistocene. The basement rocks of the Florina Basin comprise Palaeozoic gneiss, schist and Middle Triassic to Lower Jurassic crystalline limestone, marble and dolomite, which are overlaid by forty-one distinct sedimentary cycles described in three separate formations ^68^.

The Base Formation (~251–548 m) consists mainly of alternating sand, clay, and conglomerate beds, interpreted as stacked alluvial fan sequences that grade upward into fluvial deposits. A change from active tectonism and a high sediment supply to a quieter regime with less accommodation space is indicated by this vertical transition. CO _2_ accumulations have been found in the fine sand layers of these river deposits, suggesting that they could serve as carbon storage or natural gas reservoir units ^69^. The overlying Vevi Formation (~25.1–124 m) records a lacustrine depositional system characterised by 21 distinct sedimentary cycles, where marl, clay and sand dominate. Several sedimentary cycles are capped by lignite seams with a cumulative thickness of up to 6.6 m. Similar deposits are found in the neighbouring Ptolemais Basin, corresponding to Lower Pliocene ^66^.

The Lophon Formation (0–124 m) is the uppermost unit and represents a return to fluviolacustrine sedimentation during the Early Pleistocene ^66^. Lithology is composed of sand, clay, and silt found as beds and marly limestone as lenses. Cross-bedded conglomerates and sands appear in the upper parts of the stratigraphy. Lophon Formation is distinguished by coarser fluvial interbeds interspersed with fine-grained lacustrine clays and silts ^66^. This change indicates a decrease in accommodation space brought on by either increased sediment influx or decreased subsidence, which is probably related to regional elevation and the Quaternary fragmentation of the Florina-Ptolemais-Servia Basins system (Steenbrink *et al.* 2006).

The sedimentary architecture of the Florina Basin supports a viable option for natural hydrogen storage ^70^. The river sand deposits of the Base Formation have been shown to naturally accumulate CO _2_, providing a useful analogy for subsurface storage behaviour. With alternating permeable sands and impermeable layers like clays and lignites, the lithological variability of the basin offers efficient natural trapping mechanisms that hold natural CO _2_ and may also be able to hold natural hydrogen accumulation. Hydrogeochemical analyses show that groundwater quality in regions impacted by natural CO _2_ seepage has not significantly declined ^70^ indicating the existence of low permeable rocks in the vicinity exhibiting good seal capabilities.

The CO _2_ emissions at Florina are ascribed to the regional Quaternary volcanism, and are primarily of crustal origin, including also a restricted mantle input, while NE-SW trending faults serve as fluid migration conduits ^71, 72^. Between 2012 and 2014, the Florina region experienced a significant rise in micro-seismicity, with over 2000 events documented and an Mw 4.1 main shock in February 2013. With certain episodes displaying migration patterns suggestive of pore-fluid pressure diffusion, seismicity seemed to be episodic and clustered. Deep CO _2_ fluids may play a part in fault zone reactivation, as evidenced by shear-wave splitting investigations that showed anisotropy compatible with the local stress field and fault orientations ^73– 75^.

## Localised geological investigation deploying hydrogeology and geochemistry

Previous field investigations conducted as part of the Pilot Strategy Project on CO _2_ storage led to the discovery of good low porosity low permeability seal rocks, but did not yield concrete evidence of an adequate reservoir rock ^42^. To address this gap, water sampling campaigns targeting zones with elevated gas content were carried out as a proxy to help identify rock formations with potential reservoir properties. While the formations sampled are themselves unsuitable for storage due to leakage, they serve as valuable geological analogues. Identifying these leaky formations will facilitate the recognition of similar, properly bounded rock units in the field that possess the capacity to store CO _2_ securely without leakage risk. Collecting data from a) previous research conducted by Daskalopoulou *et al.*, 2018 ^21^ b) observations investigation in the field using anecdotal data, from publicly available media and c) public indications of water springs with the elevated gas yield, the authors considered the local geology and concluded that the springs presented in Table 1 are promising places for an initial investigation.

**Table 1.** Locations of water springs observed and suspected with a high possibility of gas content.

Name	N (WGS84)	E (WGS84)	Χ (Greek Grid, EGSA 87, m)	Y (Greek Grid, EGSA 87, m)	Z (m)
**Katakali**	39° 55' 31,15"	21° 40' 37,5"	301337	4421760	404
**Κivotos**	40° 14' 35,55"	21° 25' 34,62"	280926	4457643	547
**Tropeouhos**	40° 44' 25,45"	21° 26' 23,87"	283693	4512806	695
**Ammohori_1**	40° 46' 39,93"	21° 28' 50,51"	287251	4516854	633
**Ammohori_2**	40° 46' 54,80"	21° 28' 57,69"	287433	4517308	631
**Itea**	40° 50' 1,64"	21° 30' 59,80"	290459	4522988	613
**Marina**	40° 51' 44,29"	21° 29' 34,15"	288543	4526211	595
**Mesohori**	40° 53' 12,92"	21° 29' 16,50"	288208	4528956	589
**Mesocampos**	40° 53' 39,57"	21° 30' 41,36"	290218	4529721	598
**Neos Kafkasos**	40° 54' 14,50"	21° 29' 37,49"	288754	4530841	585

**Figure 5.**
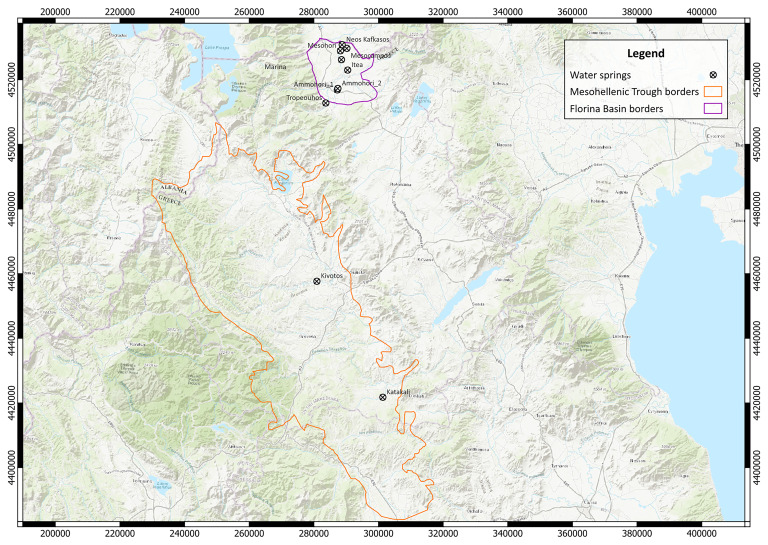
Map with the sample location in EGSA 87 grid system, scale 1:800.000, licence: CC-BY 4.0.

The local geology of each water spring and sampling area is summarised based on published geological maps and field observations. Water springs at Katakali and Kivotos emerge through formations of the Mesohellenic Trough, while spring emanations at Tropeouhos, Ammohori, Itea, Marina, Mesohori, Mesocampos and Neos Kafkasos are found at the Florina Basin ( Figure 5).

At Katakali, the near surface layers include Pliocene to Lower Pleistocene marls, sandstones, sandy marls, clays, and conglomerates of the Karperon Basin ^76^. A small lignite layer within the marls near the Sioutsa river indicates local marsh conditions, while fossil evidence (Planorbis, Neritina, and Radix ovate) suggests freshwater environments. Near basin margins, these sediments transition into unbedded fluvial-torrential deposits. The lower stratigraphy includes:

1) the Miocene (Upper Aquitanian to Tortonian) Tsotyli Fm of the MHT (<2200 m in thickness). It is composed of ophiolitic conglomerates at the base, gradually transitioning into sandstones, marls, and sandy marls. The fossil content in the transition beds includes bryozoans (Retepora gigantea), corals (Siderastrea crenulata, Ceratotrochus duodecim sostatus), gastropods (Haustator magnasporulus, Turritella sp., Gypraea sp.), and bivalves (Chlamys multistriata, Panopea intermedia), indicating a marine depositional environment, and
2) the Middle to Upper Oligocene Eptachori Fm of the MHT, consisting of ophiolitic conglomerates with lateritic lenses at the base and interbedded sandstones and marls above. This formation lies unconformably over the metamorphic basement and contains a rich microfaunal assemblage (Milliolidae, Textulariidae, Cibicides sp., Almaena sp.), pointing to a shallow marine setting during its deposition ^76^.

The stratigraphy at Kivotos sampling site comprises Quaternary alluvial sediments, such as loose sands and clays, which are underlain by Pliocene to Pleistocene fluvial and lacustrine deposits forming terrace sequences composed of loose conglomerates, blue to greenish clays, sands, and friable sandstones ^77^. The upper parts of this unit are characterised by red clays and conglomerates, suggesting alternating oxidising and reducing conditions in a dynamic depositional setting. Lower the Early Miocene (Aquitanian to Burdigalian) Tsotyli Fm of the MHT is found (<500 m in thickness), composed of conglomerates, and intercalated sandstones and clastic limestones, that exhibit rapid lateral facies changes and thinning. The fossil content (lamellibranchs, gastropods, algae and Miogypsina) suggests a shallow marine and marginal marine environment ^77^. The lowermost unit in the area consists of brecciated Middle to Upper Cretaceous limestones with rudists and marly limestones, representing the tectonically deformed remnants of an older carbonate platform that forms the geological basement of the sequence.

The stratigraphy at Tropeouhos, Ammohori, Itea, Marina, Mesohori, Mesocampos and Neos Kafkasos comprises Neogene sands, clays, and conglomerates that grade downward into massive, whitish-brown to whitish-yellow fossiliferous marly limestones, marls, and clays (<100 metres in thickness) ^68, 78^. The presence of thin lignite and xylite layers in the deeper parts indicates episodes of organic-rich deposition in marshy, low-energy environments. Fossil assemblages (Neritina, Theodoxus macedonicus) support a lacustrine to limnotelmatic depositional setting. Beneath these Neogene sediments lies the Pelagonian metamorphic basement of Palaeozoic age, composed predominantly of orthogneiss and paragneiss with intercalations of schists in the form of layers and lenses, and minor occurrences of amphibolites ^68, 78^.

Additionally, at Itea and Mesohori, the local stratigraphy includes Pleistocene conglomerates, sandstones, sands, and red clays, indicating deposition in fluvial to alluvial environments under oxidising conditions (<200 metres in thickness) ^68^. These sediments represent a transitional phase between older Neogene units and more recent Quaternary deposits, reflecting intense weathering, erosion, sediment transport, and soil formation under subaerial conditions during the Pleistocene.

## Materials and Methods

Water samples for gas measurements were collected in accordance with the UK Environment Agency Methods for sampling and analysing methane in groundwater: a review of current research and practice ^79^, as well as Capasso and Inguaggiato, 1998 ^36^ and Inguaggiato and Rizzo, 2004 ^80^. The sample collection method deployed is described below.

**Figure 6.**
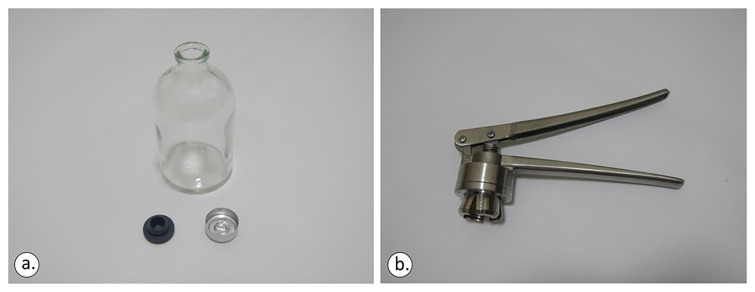
The 100ml crimp-top glass vial, the 20mm rubber closure (lower left) and aluminium cap ( **a**) and the standard stainless-steel crimper ( **b**), licence: CC-BY 4.0.

The sampling bottles were 100/125/250 ml vials crimped using a Teflon septum ( Figure 6). Vial size was determined by the target analysis: 100 ml for carbon isotopes, ≥125 ml for noble gas isotopes. The future user/adopter is advised to pay particular attention to shipping and preservation of samples, as, despite the best efforts of the authors, the first two rounds of samples could not be measured reliably for gases in the laboratory due to the hot conditions of South Europe during spring and summer.

As prerequisite actions, before filling the vials with water sample, the vials were fully filled and rinsed with the same water to be sampled from the spring samples 2 or 3 times to reduce contamination.

Prior to the sampling, the spring water was measured against dissolved O _2_ (mg/L), Conductivity (μS/cm), Temp (°C) and pH. The values of the latter were recorded in field books or digital devices. Physico-chemical conditions of the groundwater (pH, temperature, salinity) can contribute to CO _2_ dissolution or release. Differences in He and CO _2_ solubility and reactivity can determine the recorded variations in the He/CO _2_ ratio ^36^. The collection should come in doubles. In other words, two (2 nr) bottles of 100 ml, or 125 ml should be collected per location or sampling area. The first bottle is used for carbon, oxygen, hydrogen and helium isotopes, whereas the other bottle is used for gas chromatography (GC). Again, the exact size of the vials is dictated by the analysis to be performed. A balance between cost and volume collected should be established. Prior conduct with the laboratory to perform the analysis is highly recommended.

In bodies of water such as lakes, pools, seawater, or drainage galleries, as well as any location facilitating direct sample collection beneath the water's surface, it is imperative to submerge and crimp the bottles. This entails placing the bottles underwater, allowing them to fill completely while ensuring that the caps remain submerged at all times to prevent any contact with the atmosphere to avoid any air contamination during the sampling phase. In addition, when the bottle is underwater, it is easier to tightly apply the rubber closure and the aluminium cap, and ensure that no atmospheric air is still trapped in the collected sample. A standard stainless-steel aftermarket crimper able to cap 8–32 mm aluminium plastic/full aluminium and stainless steel/tight caps was used for sealing the sampling bottles.

Both the rubber closure and aluminium cap ( Figure 6) were applied and the vials were moved sideways to check if any air bubbles were visible to the naked eye. Crimping the bottle underwater might be a difficult task as the aluminium cap tends to float towards the surface.

**Figure 7.**
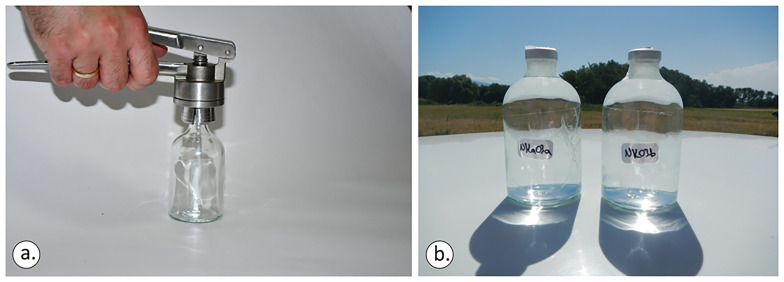
Ensuring that the bottom of the bottle and the mouth of the crimper are levelled before applying force to the crimper’s tongues ( **a**). Practice a lot after reading the crimper’s manual and before commencing the sampling process ( **b**), licence: CC-BY 4.0.

In any sampling action, it was ensured that the bottom of the bottle and the mouth of the crimper were levelled before applying force to the crimper’s tongues ( Figure 7). Otherwise, the aluminium cap will not be sealed perfectly. The sampling staff is advised to check the manual of the crimper and do considerable practice in sealing bottles before actually undertaking the sampling process. When bottles are sealed, it is advisable to check them again for bubbles under daylight and apply sealing tape around the neck of the bottle, covering also only the sides of the aluminium caps ( Figure 7).

For water sampled from taps, a tube of suitable diameter, crafted from either silicone or Teflon, is required. This tube was inserted into the bottles until reaching the bottom, enabling the water to fill the container from bottom to top. Subsequently, the tube is slowly removed, ensuring the vial is entirely filled up to the upper portion of the bottle neck. Extreme care was taken to eliminate any air bubbles during this process, followed by sealing the vial securely to prevent air contamination.

It is recommended that the final procedure be conducted within a cylinder containing an identical sample of water extracted from the tap. This approach ensures the vial is fully submerged upon reaching its capacity, facilitating underwater sealing and crimping with water acting as a barrier to prevent air entrapment.

**Figure 8.**
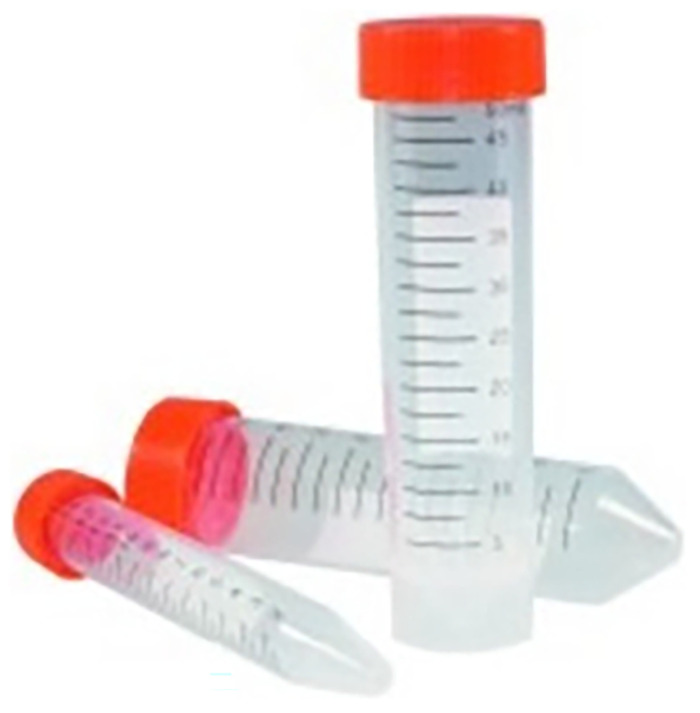
Plastic (Falcon) 50 ml sample filters, license: CC-BY 4.0.

For inductively coupled plasma mass spectrometry (ICP-MS) analysis, employing plastic sterilised (Falcon) 50ml sampler filters ( Figure 8), which have been appropriately acidified beforehand, is deemed sufficient.

After the sampling took place, a visual observation of the existence or nonexistence of bubbles was noted. Samples were appropriately numbered on a pre-agreed sampling system and metadata such as location name, coordinates, time and data of sampling, related geological formation and any other observed data were properly recorded. Recording of sample numbers in each sample bottle is vital to minimise analytical problems during the laboratory investigation. All data collected, recorded and subsequently uploaded in SESAR, the System for Earth and Extraterrestrial Sample Registration. Each sample was allocated an International Geo Sample Number (IGSN) to ensure that they are Findable, Accessible, Interoperable, and Reusable (FAIR) ^81^. The IGSN persistent identifier can link each sample with any analyses performed in any laboratory. Published data can be accessed via hyperlinked journals online. All data can be located via online geological data services or the Zenodo platform.

Four sampling campaigns were distributed across different seasons, with collections in summer 15–19 July 2023, spring 21 – 22 April 2024, late spring/early summer 5–6 June 2024, and late autumn/early winter 29 November–3 December 2024, enabling the assessment of potential seasonal effects such as temperature changes, precipitation patterns, organic matter input, and hydrological dynamics. In each case, the samples were shipped to an external accredited laboratory and specifically to the Laboratory for Stable Isotopes, Istituto Nazionale di Geofisica e Vulcanologia Sezione di Palermo in Italy for geochemical laboratory analysis that followed the below provided protocol.

Samples were analysed for He, H _2_, O _2_, N _2_, CH _4_ and CO _2_ by gas chromatography (Perkin Elmer Clarus500 equipped with a double Carboxen 1000 column system, TCD-FID detectors) using Ar as the gas carrier. Ar was analysed with a Perkin Elmer XL gas chromatograph with MSieve 5A column, TCD detector having He as carrier. Analytical uncertainties are ± 5%. Hydrocarbon analyses were performed with a Shimadzu 14a gas chromatograph equipped with a Flame Ionization Detector (FID) using He as the carrier gas. The analytical error is ≤5%.

Isotope determinations of δ ^18^O/ ^16^O and δD in water samples were performed using the equilibration technique for oxygen and water reduction (hydrogen production using granular Zn) for hydrogen. Measurements were carried out using a FinniganDelta Plus mass spectrometer (Hydrogen) and an automatic preparation system coupled with an AP 2003 IRMS (Oxygen). Analytical precision for each measurement is better than 0.2‰ for δ18O and 1‰ for δD.

Carbon isotope composition of CO _2_ was determined by using a Thermo Delta Plus XP, coupled with a Thermo TRACE Gas Chromatograph (GC) and a Thermo GC/C III interface. The TRACE GC is equipped with a Poraplot Q (25 m × 0.32 mm) column and uses Helium (5.6) as carrier gas at a constant flow of 0.9 cm ^3^ min ^−1^.

Undesired gas species, such as N _2_, O _2_, and CH _4_, are vented to the atmosphere using back-flush of He and a Sige valve. The ^13^C/ ^12^C ratios are reported as δ ^13^C _CO2_ values relative to the V-PDB standard (Vienna Pee Dee Belemnite) ^82^. Carbon isotope ratios were determined by comparing three in-house standards (δ ^13^C ranging from +0.3 ± 0.1‰ to −28.5 ± 0.3‰ vs V-PDB calibrated using a CO _2_ standard (RM8564 ^83^) with known isotopic composition (δ ^13^C = −10.45 ± 0.04‰ vs V-PDB) and two international standards (NBS 18 and NBS 19 ^82^). External precision, computed as 1σ (standard deviation) on ten measurements of the same sample, is 0.1‰. The RM8564, NBS 18 and NBS 19 standards can be obtained from the Terrestrial Environment Radiochemistry Laboratory of the International Atomic Energy Agency ( https://analytical-reference-materials.iaea.org/catalogs, web page accessed on 21/08/2025)

Carbon and Hydrogen isotopes of CH _4_, both in free gases and in dissolved gases, were measured using a Thermo TRACE GC interfaced to a Delta Plus XP gas source mass spectrometer and equipped with a Thermo GC/C III (for Carbon) and with GC/TC peripherals (for Hydrogen). The gas chromatograph was equipped with an Rt-Q Plot column (Restek 30 m × 0.32 mm i.d.) and the oven was held at a constant temperature (50 °C for carbon and 40 °C for Hydrogen). The flow rate of carrier gas (He 5.6 grade) was held at a constant flux of 0.8 cm ^3^ min ^−1^. A split/splitless injector with a split ratio from 10:1 to 80:1 was used for sample introduction, except for diluted samples (CH _4_ concentration lower than 10 mmol/mol) when direct on-column injection was performed.

The inlet system consists of a stainless steel loop with a known volume (50 μl), connected to a two-position six-port Valco® valve. Before the introduction of the sample, a vacuum of 10 ^−2^ mbar measured with an EBRO pressure gauge is ensured by a rotary vane pump. Once CH _4 _was separated from the gas mixture, it was quantitatively converted to CO _2_ by passing through a combustion oven (T = 940 °C) for ^13^C/ ^12^C ratios analysis or to H _2_ by passing it through a reactor set at a temperature of 1440 °C for ^2^H/ ^1^H ratios analysis. Each sample analysis took about 500 s.

The ^13^C/ ^12^C ratios are reported as δ ^13^C-CH _4 _values for the V-P standard and ^2^H/ ^1^H ratios are reported here as δ ^2^H-CH _4_ values with respect to the V-SMOW standard. Carbon isotope ratios were determined by comparing an in-house standard (δ ^13^C = −49.5 ± 0.2‰) calibrated using four CH _4_ standards (Isometric Instruments) with known isotopic composition (δ1 ^3^C ranging from −23.9 ± 0.3‰ to −66.5 ± 0.3‰ vs V-PDB).

Hydrogen isotope ratios were determined by comparing an in-house standard (δ ^13^C = −200 ± 2.0‰) with a CH _4_ standard with known isotopic composition (δ ^2^H = −186.1 ± 3.0‰ vs V-SMOW). External reproducibility, estimated as 1σ (standard deviation) on ten measurements of the same sample, is 0.2‰ and 2.0‰ for carbon and hydrogen isotopes, respectively.

In CO _2_-dominated gases having CH _4_ concentrations lower than 1000 μmol/mol, the analyses of the isotope ratios of methane were carried out in the headspace gas samples collected using pre-evacuated 60 mL glass flasks ﬁlled with 20 mL of a 4 N NaOH solution.

The abundance and isotope composition of He, and the ^4^He/ ^20^Ne ratios, were determined by separately admitting He and Ne into a split flight tube mass spectrometer (Helix SFT). Helium isotope compositions are given as R/RA, where R is the ( ^3^He/ ^4^He) ratio of the sample and RA is the atmospheric ( ^3^He/ ^4^He) ratio (RA = 1.386 × 10 ^−6^). The analytical errors were generally < 1%.

Location names, sampling date and coordinates of all new sampling sites together with raw chemical results can be found as supplementary material at https://doi.org/10.5281/zenodo.16914636 ^84^. 

## Results

**Figure 9.**
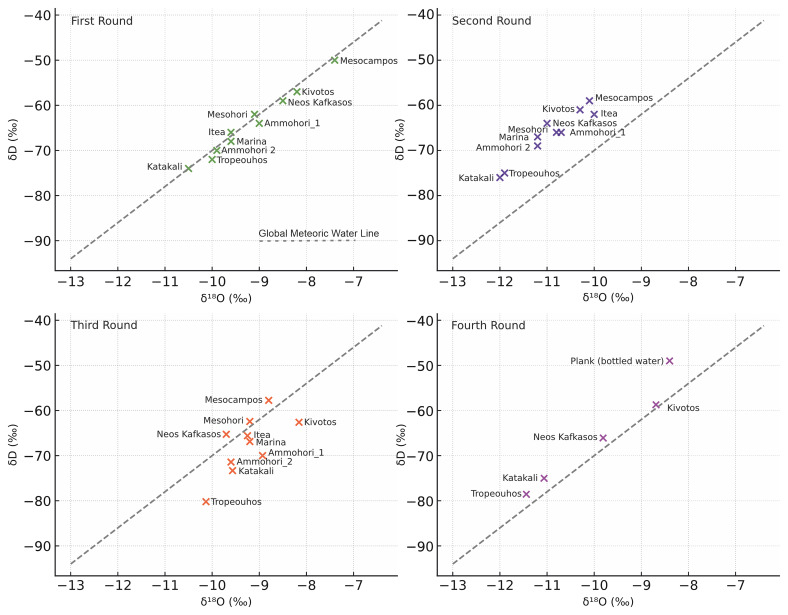
Stable Isotope composition of water samples collected from June 2023 to December 2024 with reference to the Global Meteoric Water Line (GMWL), licence: CC-BY 4.0.

Below are presented the results from all four sampling campaigns, starting with the isotope data analysis ( Table 2– Table 5). As the samples are from groundwater, the Global Meteoric Water line was deployed to analyse the data rather than the Local Meteoric Water line, which relies on precipitation samples only ^85^ ( Figure 9).

**Table 2.** Water samples collected between 15-19/07/2023, laboratory data analysis provided at 06/09/2023, first sample campaign, rounded to two significant figures.

N°	Spring location name	δD _H20_	δ ^18^Ο _Η2Ο_	Dissolved O _2_ (mg L ^-1^)	Conductivity (μS cm ^-1^)	Temp (°C)	pH
**1**	Tropeouhos	-72	-10.0	7.9	-	17.5	6.04
**2**	Mesocampos	-50	-7.4	0.19	1686	16	6.06
**3**	Neos Kafkasos	-59	-8.5	0.76	575	14.1	6.08
**4**	Ammohori 1	-64	-9.0	8.3	920	17.5	6.07
**5**	Itea	-66	-9.6	2.73	1017	16.5	6.03
**6**	Mesohori	-62	-9.1	1.09	478	14	6.03
**7**	Ammohori 2	-70	-9.9	7.1	319	20.5	6.01
**8**	Marina	-68	-9.6	6.3	338	25.6	6.04
**9**	Kivotos	-57	-8.2	0.24	850	15.3	5.4
**10**	Katakali	-74	-10.5	0.25	1447	18	6.0

**Table 3.** Water samples collected between 21-22/04/2023, laboratory data analysis provided at 15/05/2024, second sample campaign.

N°	Spring location name	δD _H20_	δ ^18^Ο _Η2Ο_	Dissolved O2 (mg L ^-1^)	Conductivity (μS cm ^-1^)	Temp (°C)	pH
**1**	**Tropeouhos**	-75.0	-11.9	8.85	1803	14.4	7.5
**2**	**Mesocampos**	-59.0	-10.1	2.59	1984	13.5	5.67
**3**	**Neos Kafkasos**	-64.0	-11.0	1.60	468	14.5	5.42
**4**	**Ammohori 1**	-66.0	-10.7	7.06	971	12	6.49
**5**	**Itea**	-62.0	-10.0	2.91	972	13	5.61
**6**	**Mesohori**	-66.0	-10.8	2.50	450	13	6.5
**7**	**Ammohori 2**	-69.0	-11.2	7.78	318	14.8	5.7
**8**	**Marina**	-67.0	-11.2	8.15	347	14.1	6.29
**9**	**Kivotos**	-61.0	-10.3	1.75	803	14.9	7.25
**10**	**Katakali**	-76.0	-12.0	1.40	1440	16.1	7.5

**Table 4.** Water samples collected between 05-06/06/2024, laboratory data analysis provided at 24/07/2024, third round campaign.

N°	Spring location name	δD _H20_	δ ^18^Ο _Η2Ο_	Dissolved O _2_ (mg L ^-1^)	Conductivity (μS cm ^-1^)	Temp (°C)	pH
**1**	Tropeouhos	-80	-10.1	0.78	1694	20.0	7.04
**2**	Mesocampos	-58	-8.8	1.72	1809	20.3	6.01
**3**	Neos Kafkasos	-65	-9.7	1.05	477	17.0	5.83
**4**	Ammohori 1	-70	-8.9	4.87	968	21.0	6.76
**5**	Itea	-66	-9.3	2.63	712	16.1	5.83
**6**	Mesohori	-62	-9.2	2.27	485	16.0	6.22
**7**	Ammohori 2	-71	-9.6	7.33	301	21.4	6.20
**8**	Marina	-67	-9.2	7.00	329	22.9	6.56
**9**	Kivotos	-63	-8.2	1.05	781	18.8	7.65
**10**	Katakali	-73	-9.6	7.22	1468	18.0	8.48

**Table 5.** Water samples collected between 29/11/2024 – 3/12/2024, laboratory data analysis provided at 06/03/2025, fourth campaign.

N°	Spring location name	δD _H20_	δ ^18^Ο _Η2Ο_	Dissolved O2 (mg L ^-1^)	Conductivity (μS cm ^-1^)	Temp (°C)	pH
**1**	**Katakali**	-11.1	-75	0.26	1505	15.1	8.04
**2**	**Kivotos**	-8.6	-58	0.25	855	14.3	7.75
**3**	**Tropeouhos**	-11.5	-78	0.41	1862	14.2	7.12
**4**	**Neos Kafkasos**	-9.8	-66	1..22	487	14.3	6.03
**5**	**Katakali-2**	-11	-75	0.26	1505	15.1	8.04
**6**	**Kivotos-2**	-8.8	-59	0.25	855	14.3	7.75
**7**	**Tropeouhos-2**	-11.4	-79	0.41	1862	14.2	7.12
**8**	**Plank (bottled water)**	-8.4	-49	-	-	-	-

In the first sample campaign ( Table 2, Summer 2023), most samples plot in close proximity to the Global Meteoric Water Line ^86, 87^, indicating a meteoric origin with little evaporation enrichment ^88^ ( Figure 9). Katakali and Tropeouhos in the first round ( Table 2) are the most depleted in both isotopes, indicating a colder recharge. Mesocampos and Kivotos are relatively enriched, possibly indicating partial evaporation ^87^. Since the elevations of all sampling locations are relatively similar, altitude differences are unlikely to be the primary factor driving the observed isotopic variability. This original investigation did not provide any distinct chemical signature; however, during the Katakali sample collections (#10), there were clear observations of gas bubbles in the water sample. This is due to pressure decreases, causing these dissolved gases to come out of solution and form bubbles.

The second round of data ( Table 3) indicated that Tropeouhos, Mesocampos and Neos Kafkasos had a shift towards depletion of isotopes, which indicates hydrologic change. This can be explained by seasonal input ^89^. The first batch was collected in Summer (June 2023) during a low rainfall season, whereas the second batch was collected in Spring (April 2024) during a high rainfall season. This is consistent with a well-mixed aquifer or steady-state recharge conditions typical of early spring. This sample location above the GMWL ( Figure 9) suggests that precipitation during the winter had enough time to infiltrate deeper into the ground and homogenise with groundwater before sampling ^85, 86, 90, 91^.

The third round of samples collected were notably more depleted in δD and δ ^18^O and with higher deviation from GMWL related to rainfall patterns and evaporation, which is consistent with the June sampling period ( Table 4). Still, most of the samples are in proximity to the GMWL, indicating that rainfall is still the prevailing factor ( Figure 9).

The fourth batch of samples collected mostly in Winter (December) plots above the GMWL, suggesting winter recharge from isotopically lighter sources indicated by the isotope depletion ( Table 5). The dispersion of the samples may reflect heterogeneity in recharge sources, mixing with deeper groundwater or longer water to rock contact time ^85, 90^, Figure 9.

In all sampling campaigns, the pH measured was either slightly alkaline, with values around 7.5 or slightly acidic and values around 5.4. There was only one exception in the third sample campaign, in the Katakali water sample, which registered an alkaline pH of 8.48.

The ICP chemical analysis of the second round presented in Table 6 revealed that all samples had high detectable levels of strontium (Sr) and barium (Ba) in the scale of μg L ^-1^. Concentrations remained well below drinking water guideline thresholds) which, according to the United States Environment Protection Agency, is 2 mg L ^-1^ for Ba and 4 mg L ^-1^ for Sr ( National Primary Drinking Water Regulations | US EPA, web page accessed on 18/07/2025). The Si concentrations varied from 7.7 to 59.6 mg L ^-1^, indicating variable rock silicate interaction ^92, 93^.

**Table 6.** ICP chemical analysis of water samples from Kivotos Ammohori 1, Ammohori 2, Itea, Marina, Mesohori, Neos Kafkasos, Katakali, Tropeouhos, Mesocampos locations in μg L ^-1^.

Spring location	Li	Be	B	Al	Ti	V	Cr	Mn	Fe	Co	Ni	Cu	Zn	As	Se	Br	Rb	Sr	Mo	Cd	Sn	Sb	Cs	Ba	Tl	Pb	Bi	U	Si
**Kivotos**	8	<0.02	269	4	0.10	0.05	0.19	5	7	0.01	<0.1	<0.1	<0.1	0.02	0.11	466	0.97	591	0.14	<0.01	0.01	0.00	0.01	67	<0.01	0.01	0.01	0.18	15000
**Ammohori 1**	3	0.16	41	1455	87	5	5.01	37	2807	1	7.6	6	177	0.44	1.2	128	4.4	660	0.24	<0.01	0.23	0.03	0.25	89	0.03	4.77	0.02	1.99	18000
**Ammohori 2**	13	0.22	15	7	0.20	0.75	0.86	10	53	0.03	9.7	47	65	0.05	0.27	88	0.31	281	0.12	<0.01	0.02	0.02	0.00	24	<0.01	1.22	<0.01	0.08	27000
**Itea**	3	0.03	37	40	0.15	0.92	0.63	18	22	0.12	3.7	9	30	0.31	3.2	47	0.57	260	1.09	<0.01	0.04	0.04	0.00	61	0.01	0.23	<0.01	6.07	8000
**Marina**	8	0.04	15	25	0.63	2	2.64	0.29	16	0.03	4.1	1	0.34	0.66	0.41	41	0.39	208	0.19	<0.01	<0.01	0.01	0.00	38	<0.01	0.01	<0.01	0.52	26000
**Mesohori**	7	0.02	90	146	11	0.71	0.58	682	247	2	8.1	15	76	0.15	0.37	58	0.99	214	0.18	0.02	6.03	0.18	0.02	54	0.01	7.89	0.05	0.21	11000
**Neos ** **Kafkasos**	8	0.04	60	30	0.12	0.79	0.12	436	10	2.03	7.3	1	4.7	0.44	0.80	44	0.94	260	0.31	<0.01	0.01	0.01	<0.01	99	0.01	0.09	<0.01	0.32	14000
**Katakali**	132	<0.02	933	5	0.41	0.19	0.61	8	221	0.40	9.0	<0.1	<0.1	0.13	0.17	185	1.1	544	0.10	<0.01	<0.01	<0.01	0.02	156	<0.01	<0.01	<0.01	0.00	60000
**Tropeouhos**	407	1.03	7160	92	1.8	0.25	0.23	444	1010	0.30	0.2	<0.1	0.9	1.5	0.33	303	39	902	6	<0.01	<0.01	0.01	31.12	11	<0.01	0.20	<0.01	0.09	17000
**Mesocampos**	5	0.03	268	17	0.34	5	0.47	50	57	1	23.6	21	41	1.7	2.5	362	42	970	3	<0.01	0.06	0.08	0.05	183	0.05	0.31	<0.01	1.17	34000
***error %***	*11*	*8.90*	*3*	*10*	*11*	*7*	*2.30*	*3*	*12*	*2.00*	*12.8*	*14*	*2.6*	*5.1*	*14.3*		*2.2*	*2*	*5*	*12*	*8.20*	*6.70*	*1.70*	*4*	*4.80*	*10.10*	*-*	*2.10*	11000

Regarding the rest of the chemical elements identified Tropeouhos and Ammohori 1 had high levels of Fe (1010 and 2807 μg L ^-1^), Mn (37 and 444 μg L ^-1^), and Al (92 and 1455 μg L ^-1^), respectively. Ammohori 1 also showed elevated Ti (86 μg L ^-1^) and Zn (176 μg L ^-1^), while Tropeouhos had significantly high concentrations of Li (406 μg L ^-1^), B (7160 μg L ^-1^), and Br (303 μg L ^-1^).

The Mesohori water sample had notable values of 132 μg L ^-1^ of Al, 682 μg L ^-1^ of Mn and 247 μg L ^-1^ of Fe. The Neos Kafkasos water sample showed Mn values of 436 μg L ^-1^. The Katakali sample had prominent values of 132 μg L ^-1^ of Li, 933 μg L ^-1^ of B, 220 of Fe μg L ^-1^, 185 μg L ^-1^ of Br. Mesokampos had 363 μg L ^-1^ of Br. All other chemical elements detected from the second water sampling campaign were of considerably lower value.

The chemical composition of groundwater samples collected during the second campaign reflects a range of geochemical processes influenced by water–rock interaction and rock differences. The Tropeouhos and Ammohori_1 sample chemical results indicate active water and rock interaction involving iron-bearing minerals that undergo redox processes with Fe ^3+^ (insoluble ferric) → Fe ^2+^ (soluble ferrous) in anoxic environments ^92, 94^. High concentration of Fe can be associated with dissolution of iron bearing minerals or the serpentinization process ^70^. Both processes favour natural hydrogen production ^25, 34, 95^. The same applies to a lesser extent for the Mesohori and Katakali samples. Of particular interest are the Katakali and Tropeouhos samples that had high detectable levels of Li (131 and 406 μg L ^-1^, respectively), indicating deep circulation water interaction with igneous or silicate rocks. This deeper circulation, also supported by isotopic evidence provided in Figure 9, favours the generation and preservation of natural hydrogen conditions ^25^.

The geochemical analysis from the third sampling campaign presented in Table 7 revealed further information related to the gas component of the water samples. The Katakali water sample analysis provided values 4.4 ppm of H _2_ coupled with 3704 ppm of CH _4_, suggesting active geochemical processes related to either organic degradation or serpentinisation. The Kivotos sample showed values of 29 ppm of He, 1.7 ppm of H _2_, and 31,400 ppm of CH _4_. The latter consists of anecdotal public observations suggesting strong methane generation potential and possible mantle-derived gas contribution to gas mix phase ^96^.

**Table 7.** Gas chemical analysis as a result of the third water sampling campaign, n.a denotes not enough sample quantity to perform the chemical analysis for that specific element.

Spring location	He (ppm)	H _2_ (ppm)	O _2_ (%)	N _2_ (%)	CO (ppm)	CH _4_ (ppm)	CO _2_ (%)	Gas volume (CC per 120 CC of sample)	He (×10 ^−4^ CC L ^-1^ STP)	H _2_ (CC L ^-1^ STP)	O _2_ (CC L ^-1^ STP)	N _2_ (CC L ^-1^ STP)	CO (×10 ^-6^ CC L ^-1^ STP)	CH _4_ (×10 ^-6^ CC L ^-1^ STP)	CO _2_ (CC L ^-1^ STP)
**KATAKALI**	n.a	4.4	5.22 ×10 ^-2^	18.14	n.a	3704	0.79	6.4	0.0	0.00	0.04	17.0	<LOQ	362.5	8.90
**ΚIVOTOS**	29	1.7	4.41 ×10 ^-2^	19.87	n.a	31400	1.58	6.4	16.4	0.00	0.02	11.3	<LOQ	1778.4	0.90
**TROPEOUHOS**	1403		3.66 ×10 ^-2^	24.81	n.a	89	2.16	6.4	794.6	0.00	0.02	14.0	0.0	5.0	1.20
**AMMOHORI_1**	n.a		1.65	19.02	0.2	1.2	7.08	6.6	0.0	0.00	0.96	11.1	12.5	0.7	4.10
**AMMOHORI_2**	n.a		5.99	19.88	0.2	1.1	7.87	7.2	0.0	0.00	3.82	12.7	13.9	0.7	5.00
**ITEA**	n.a		1.60 ×10 ^-1^	2.19	n.a	4.1	60.68	14.4	0.0	0.00	0.20	2.8	0.0	52.3	77.30
**MARINA**	n.a		4.35	16.34	0.2	0.8	4.13	6.2	0.0	0.00	2.39	9.0	7.3	0.4	2.30
**MESOHORI**	n.a		3.1 ×10 ^-1^	16.42	n.a	9	3.3	6.8	0.0	0.00	0.19	10.0	0.0	54.2	2.00
**MESOCAMPOS**	n.a		7.01 ×10 ^-2^	2.02	n.a	34	60.22	12.4	0.0	0.00	0.08	2.20	0.0	373.1	66.10
**NEOS KAFKASOS**	6	1.9	1.00 ×10 ^-1^	9.48	2.2	n.a	40.01	10	5.3	0.00	0.09	8.40	779	0.0	35.40

The Tropeouhos sample registered notable He levels (1403 ppm), accompanied by low CH _4_ concentrations at 89 ppm. This could be associated with deep-seated helium degassing coupled with some microbial or thermogenic methane input ^97, 98^.

Samples Ammohori_1 and Ammohori_2 registered elevated CO _2_ concentrations of approximately 7–8%, while methane remained low at around 1 ppm. In addition, the Itea sample contained 60.7% CO _2_ and 4 ppm of CH _4_, suggesting a dominant CO _2_ component in the gas phase. This is consistent with the accumulation of natural CO _2_ in the Florina basin in the Tertiary formation and its leakage through fractures and permeable rocks ^70^. The Mesohori sample registered 9 ppm of CH _4_, while Mesokampos contained 34 ppm of CH _4_ along with approximately 60% CO _2_, pointing to variable organic or volcanic degassing influence ^99^. Finally, the Neos Kafkasos sample exhibited 6 ppm of He, 2 ppm of H _2_, and a substantial 40% CO _2_, indicating multi-gas signatures possibly linked to deep fluid migration through faulted rocks ^71, 99^.

Following the scientifically interesting results of the third campaign, a fourth one commenced with a focus on four promising locations of Katakali, Kivotos, Tropeouhos and Neos Kafkasos. To further elucidate the origin of the gas phase, the geochemical investigation considered the following analysis:

1. He
2. H _2_
3. CH _4_
4. CO _2_
5. Helium isotopes ( ^3^He, ^4^He)
6. Deuterium of hydrogen (δ ^2^H-H _2_)
7. Deuterium of methane (δ ^2^H-CH _4_)
8. Deuterium of water (δ ^2^H-H _2_O)
9. Oxygen-18 of carbon dioxide (δ ^18^O-CO ^2^)
10. Oxygen-18 of water (δ ^18^O - H _2_O)
11. Carbon-13 of methane (δ ^13^C-CH _4_)
12. Carbon-13 of carbon dioxide (δ1 ^8^O-CO _2_)
13. Carbon-13 of DIC (δ ^13^C-DIC)
14. Carbon-14 of DIC ( ^14^C-DIC)

This time, a blind sample system with duplicates was used during the laboratory analysis, together with a sample of water table as a blanket to increase the reliability of the chemical analysis results. Thus, the laboratory received codes only and not the actual locations of the samples. In all cases, the duplicate samples showed almost similar values. The results of the fourth campaign are presented in Table 8.

**Table 8.** Targeted Gas chemical analysis as a result of the fourth water sampling campaign.

Spring location	He (ppm)	H _2_ (ppm)	O _2_ (%)	N _2_ (%)	CH _4_ (ppm)	CO (ppm)	CO _2_ (%)	Gas volume (cc per 120 cc of sample)	He (×10 ^–4^ cc L ^-1^ STP)	H _2_ (cc L ^-1^ STP)	O _2_ (cc L ^-1^ STP)	N _2_ (cc L ^-1^ STP)	CH _4_ (×10 ^-3^ cc L ^-1^ STP)	CO (×10 ^-3^ cc L ^-1^ STP)	CO _2_ (cc L ^-1^ STP)	total Alk	d ^13^C _TDC_	d ^13^C _CH4_	dD _CH4_
**Katakali**	-	-	0.12	4.07	300370		2.33	7.4	0.0	0.0	0.10	4.19	14558.3	0.0	26.90	15.8	13.9	-69.5	-251
**Kivotos**	43	-	0.19	19.08	43000		1.67	6.2	27.9	0.0	0.14	17.63	1833	0.0	19.10	6.3	-14.2	-75.5	-107
**Tropeouhos**	-	-	0.21	22.56	81		2.4	6	0.0	0.0	0.15	20.44	4.0	0.0	27.40	2.00	3.9	-	-
**Neos Kafkasos**	10	-	0.58	8.45	7		49.9	10	9.8	0.0	0.62	10.65	3.3	0.0	587.10	4.2	-1.2	-	-
**Katakali-2**	-	-	0.1	6.08	312300		2.44	7.6	0.0	0.0	0.09	6.37	15440.5	0.0	28.20	15.7	14.4	-69.8	-230
**Kivotos-2**	44	-	0.24	20.04	44100		1.73	5.8	27.0	0.0	0.17	17.81	1903.3	0.0	19.70	6.4	-15.8	-69.5	-92
**Tropeouhos-2**	1.4	-	0.21	22.47	81		2.49	5	0.9	0.0	0.15	20.36	4.04	0.0	28.40	2.1	1.9	-	-
**Plank (bottled water)**	-	-	3.3	19.86	2.7	19.5	0.94	5.6	0.0	0.0	2.26	17.29	0.1	1.8	10.70	4.0	-11.2	-	-

In this final fourth round, no hydrogen gas was registered. However, the Katakali samples showed a mean value of 306335 ppm CH _4 _with δ ^13^CCH _4_ values around –70‰ and δDCH _4_ near –230‰. On the same note, the Kivotos samples registered a mean of 43.5 ppm of He coupled with a mean 43550 ppm CH _4_ with δ ^13^CCH _4_ at –75.5‰. The Tropeouhos samples showed a mean of 81 ppm of CH _4_, in this case, the first sample showed no He values, whereas the second sample 1.4 ppm of He. The Neos Kafkasos, although sampled in duplicates, only one sample survived and was analysed. The sample that survived showed a value of 10 ppm of He and 7 ppm of CH _4_. For the Neos Kafakasos sample, there was no δ ^13^CCH _4_ available. The δ ^13^CTDC was at –1.2‰) and δD was at –66‰).

## Discussion and results interpretation

Most samples were slightly acidic (around pH 5.4), suggesting interaction with silicate-rich lithologies, likely weathered ophiolites, or the influence of organic-rich soils. In contrast, the Katakali sample exhibited a distinctly alkaline pH (8.48), potentially indicating interaction with carbonate rocks (e.g. Triassic–Jurassic limestones) or serpentinized ultramafics undergoing low-temperature alteration, which commonly release hydroxide ions and elevate pH ^10, 25, 34^.

The sedimentary series of the Mesohellenic Trough exhibits distinct geological and mineralogical characteristics that reflect its diverse depositional histories and diagenetic evolution. Sandstones from the Eptachori Fm, are fine-grained and well-bedded, siliceous in composition and display bioturbation features and fossils of plant fragments ^100^. Quartz (<37 wt.%) is the dominant mineral, followed by calcite (<29 wt.%), albite (<13 wt.%), and muscovite (<13 wt.%). Minor mineralogy includes dolomite, chamosite, biotite, titanite, pyrite, crichtonite, actinolite and zircon ^100^. Coupled with carbonate-rich microcrystalline cement, these features indicate deposition in a marine-influenced or lacustrine setting. Larger grain size, a mixed lithic content, and a calcareous composition characterise sandstones from the Pentalofos Fm. Calcite (<41 wt.%), dolomite (<18 wt.%), and quartz (<15 wt.%) with a notable feldspathic component (albite <12 wt.%) characterise mineralogy. Minor constituents include microcline, muscovite, aragonite, and traces of chlorite, titanite, chromite, pyrite, Fe-chromite, actinolite, apatite, andradite and enstatite. These characteristics reflect depositional settings of higher-energy with significant detrital input, suggesting a mixed depositional environment influenced by the erosion of the ophiolitic rocks surrounding the Mesohellenic Trough. Marly sandstones from the Tsotyli Fm show medium bedding, massive tabular structures, and a mixed siliceous-calcareous composition. Calcite (30 wt.%), quartz (29 wt.%), and albite (21 wt.%) are the main minerals, while the fine-grained, well-cemented matrix contains accessory muscovite, chlorite, dolomite and aragonite, and traces of microcline, zircon and jacobsite ^100^. Coupled with a moderate macro-porosity, these aspects suggest deposition in a high-energy environment with significant siliciclastic input.

The samples from Ammohori 1 and Tropeouhos displayed high levels of Fe (up to 2807 µg L ^-1^), Mn (up to 444 µg L ^-1^), and Al (up to 1455 µg L ^-1^), indicating reducing conditions that mobilise these elements from iron and manganese oxides and aluminosilicates. Such elevated concentrations are related to reductive dissolution of Fe/Mn oxides under anoxic conditions ^70, 101^ contribution from ophiolitic or volcanoclastic host rocks ^102, 103^, or possibly weathering of feldspars ^104^ or clay-rich layers typically found in flysch or schist rocks ^70, 105, 106^ typically found in flysch.

The exceptionally high concentrations of Li (up to 406 µg L ^-1^), B (up to 7160 µg L ^-1^), and Br (up to 363 µg L ^-1^) in Tropeouhos, Katakali, and Mesokampos samples suggest interaction with deeper, evolved fluids. These elements are typically enriched in hydrothermal systems ^70^, marine sedimentary or evaporite-hosted aquifers ^107^ or fluids with long residence times and extensive water–rock contact ^103^. Boron and bromine enrichment may also indicate a marine or connate water signature, particularly in the Mesokampos sample, or mixing with residual basinal brines ^107, 108^.

To identify the actual source of the aforementioned discussed elements, an elaboration is provided. In the Florina Basin, the non-lignite intraseam sediments include both marl and sand lithologies, are primarily characterised by a dominance of illite and mica, alongside significant proportions of kaolinite, quartz, ferroan chlorite, and albite feldspar. Carbonate minerals are largely absent, with only traces of dolomite sporadically occurring, while the clay fraction includes smectite and irregular smectite/illite ^109^. Sand samples contain slightly higher amounts of feldspar (<7.2 wt.% albite) and mica (<28.6 wt.%), whereas the marl samples show slightly higher proportions of clay minerals, including illite (37.8 wt.%), kaolinite (17.4 wt.%), and ferroan chlorite (9.3 wt.%) ^109^. These characteristics indicate that deposition occurred in a fluvio-lacustrine environment with variable energy conditions. The dominance of illite, mica, and kaolinite, along with quartz and albite, suggests weathering and erosion of felsic or metamorphic source rocks under humid conditions. Marls being enriched in clay minerals and ferroan chlorite reflect lower-energy, possibly more stagnant depositional settings, while the sands, with higher quartz and feldspar content, point to intermittent higher-energy fluvial input. The scarcity of carbonates indicates limited diagenetic processes and supports deposition in a non-marine, siliciclastic-dominated basin with contributions from both soil-forming and fluvial processes. Furthermore, Gemeni *et al.*, 2015 ^70^ have supported that the origin of Fe, Mn and Br is not related to hydrothermal systems but interaction with deeper igneous rocks ^70^.

While all samples had detectable Sr and Ba, their concentrations were below regulatory thresholds. Their presence is consistent with the dissolution of carbonate and feldspathic minerals. Sr points to contributions from marl, limestone, or plagioclase-rich rocks common ^110^ in the Mesozoic sequences of Western Macedonia, despite the variations suggesting diversified geological histories. Sandstone samples from the central parts of the Mesohellenic Trough are classified mineralogically as feldspathic litharenites ^100^. Similar mineralogical and geochemical characteristics are reported from the southeastern part of the Mesohellenic Trough, where sandstone samples are classified geochemically as graywackes and litharenites ^111^.

The Katakali sample's combination of high pH, elevated Li and B, and relatively low transition metal concentrations points toward interaction with serpentinized ultramafic rocks. These lithologies commonly generate hyperalkaline fluids through serpentinization reactions and are known potential sources of natural hydrogen ^102, 103^.

The third-round water samples from the West Macedonia region present a complex mixture of dissolved gases, reflecting interactions between geological formations, redox conditions, and possibly deep-seated fluid sources.

Tropeouhos (1403 ppm He) and Kivotos (29 ppm He) samples indicate significant helium enrichment, which is not produced in shallow processes. Such high He levels suggest input from a radiogenic source in crystalline basement rocks (U/Th decay) ^112^, or mantle-derived fluids migrating through deep-seated fault systems ^112^. The Neos Kafkasos, even at the 6 ppm He complements this due to the proximity to Tropeouhos.

High CO _2_ concentrations in Itea (60.7%), Mesokampos (~60%), and Neos Kafkasos (~40%), including Ammohori_1 and _2 (~7–8% CO _2_) are related to the Florina basin, where natural CO _2_ volcanic in origin have accumulated in the Miocene fluvial sandstones ^69, 99^. The accumulations migrating via faults have been detected from depths of 296–338 m and 366–372 m below the surface and are well reported ^69^. They also provide CO _2_ analogues for CO _2_ storage, which is also related to the Pilot Strategy Project ^42^.

Katakali (4.4 ppm H _2_), Kivotos (1.7 ppm H _2_), and Neos Kafkasos (2 ppm H _2_) exhibit detectable hydrogen, a rare but significant indicator of water–rock reactions, that relates to a ) particularly serpentinization of ultramafic rocks (e.g., ophiolites), b) radiolysis of water in fractured rocks under the influence of natural radioactivity c) anaerobic corrosion of Fe-bearing minerals, particularly under reducing conditions. These processes are common in tectonically active zones, where fluid pathways are enhanced ^10, 16, 17, 19, 25, 34, 46^.

**Figure 10.**
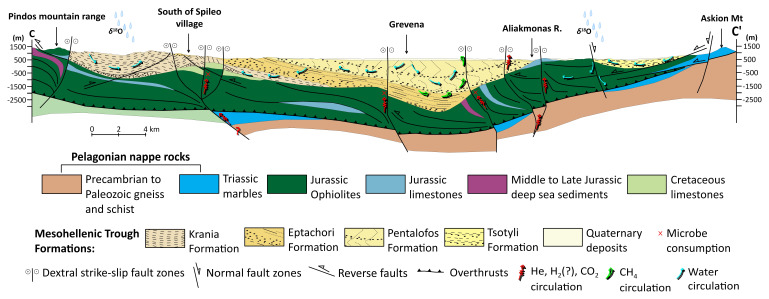
Geological cross section of Mesohellenic Basin with interpretative subsurface gas generation and migration mechanisms, licence: CC-BY 4.0.

In light of the data obtained from round 4 the Katakali samples, the high methane concentrations (>300,000 ppm) in both duplicates and slightly alkaline pH (~8) point toward a deep-seated methane source. The absence of helium and hydrogen, combined with δ ^13^CCH _4_ values around –70‰ and δDCH _4_ near –230‰, strongly supports biogenic methane via microbial CO _2_ reduction ^96, 113^, although further investigation may be needed to properly distinguish between bacterial imprint or diffusive fractionation ^97^. The moderately enriched δD and δ ^18^O of water suggest some evaporation, but the dominant control appears to be long residence time and water–rock interaction with organic-rich strata (possibly lignites or deep carbonate units) ^114^. The interpretation from the data received from the sample reflects a closed-system ^114^ microbially dominated environment with limited gas migration, depicted in Figure 10. Despite high CH _4_, absence of He and H _2_ implies limited serpentinization or mantle influence ^96, 113, 115^. It is noted that Daskalopoulou *et al*. 2018 report traces for helium and hydrogen which was not identified in the Katakali samples during this campaign. This is not necessarily contradictory and it can be for various reasons, including sampling method, sampling preservation and delayed time analysis, including analytical error. However, the high methane concentrations with biotic origin are confirmed by both studies.

On the contrary, in Kivotos samples, the presence of both He (43–44 ppm) and H _2_ (1.7 ppm) with CH _4_ (~43,000 ppm) suggests a hybrid gas system. Helium and hydrogen point to a possible serpentinization signature, typical for ultramafic lithologies (e.g. ophiolites) ^112, 113^. CH _4_ may derive from either microbial or abiotic origin, with low δ ^13^CCH _4_ (–75.5‰) still favouring a microbial pathway ^96, 116^. This may indicate a mixed or transitional methane, either microbial with some thermogenic input or some hydrogen exchange during migration.

Slight enrichment in δD and δ ^18^O is consistent with moderate evaporation or prolonged water–rock interaction. A hybrid gas system dominated by microbial CH _4_ with secondary abiotic inputs, possibly from ultramafic serpentinization or water–rock reactions, can potentially describe the main gas mechanism in this case.

In Tropeouhos, with its exceptionally high helium (up to 1403 ppm) and low CH _4_ (81 ppm), it indicates a strong mantle-derived or radiogenic He component. The low CH _4_ and absence of H _2_ suggest minimal microbial or abiotic CH _4_ production ^31, 96, 113, 115^ and possible degassing along deep fault pathways. Dominated by radiogenic/mantle degassing, possibly along deep fault structures. This site may represent a degassing zone rather than a storage environment.

The Neos Kafkasos with low but detectable He (10 ppm) and H _2_ (2 ppm) combined with CO _2_ at ~50% suggest mantle or deep carbonate reservoir interaction. Although δ ^13^CCH _4_ was not obtained due to the low concentration of CH _4_, the data availability on δ ^13^C _TDC_ (–1.2‰) and δD (–66‰) suggests thermogenic gas mixing ^117^. The reducing conditions and high CO _2_ levels provide evidence for carbonate dissolution, blended together with the volcanic origin of trapped CO _2_^69, 99^. Given the proximity of Mesokampos and Itea, which had elevated CO _2_% levels, it suggests a CO _2_-dominated system, possibly involving carbonate dissolution, or closed-system redox processes. The presence of He and H _2_ supports some deep-origin fluid connectivity.

Kivotos and Kivotos-2 reported δ ^13^CCH _4_ ≈ -75.5 to -69.5‰, δDCH _4_ ≈ -107 to -92‰. These samples have δ ^13^CCH _4_ within the biogenic range, but δDCH _4_ values are much less depleted, potentially indicating mixed or transitional methane, either microbial with some thermogenic input or some hydrogen exchange during migration.

## Conclusion

The geochemical investigation supports the interpretation of multiple water sources or flow paths, including both shallow, oxygenated meteoric water and deeper, more evolved fluids enriched in light elements. Overall, the results point towards a gas migration dominant mechanism which along the gas miratory route chemical changes occur with evidence of large reservoirs still to be found apart from the well known natural CO _2 _reserves. Where He and H _2_ are present (e.g., Kivotos), the system involves both deeper lithological interaction (ultramafics/fracture pathways) and microbial methane generation in overlying sediments or mixed reservoirs.

In such cases, isotopes still point to dominantly biogenic CH _4_ even if the gas migration system involves ophiolitic or deep rocks. Specifically, biogenic methane dominates in confined organic-rich environments (Katakali), while He and H _2_ signals at Kivotos and Tropeouhos suggest deep crustal or mantle-derived fluids, potentially linked to serpentinization or radiogenic decay. Sites like Kivotos are most promising for mixed hydrogen generation, with some abiotic gas potential. Tropeouhos may serve more as a gas migration indicator, while Neos Kafkasos indicates natural CO _2 _degassing rather than accumulation.

Where He and H _2_ are also present (e.g., Kivotos), the system may involve both deeper lithological interaction (ultramafics/fracture pathways) and microbial methane generation in overlying sediments or mixed reservoirs. In such cases, isotopes still indicate biogenic CH _4_ even if the gas migration mechanism involves ophiolitic or deep rocks. On the same note H _2_ is difficult to find in the surface springs due to easy microbial consumption in the water ^118– 123^.

However, with reference to the present chemical analysis from Katakali samples and previous research from Daskalopoulou *et al.*, 2018 ^21^ that reported helium and hydrogen concentration an additional explanation may be that biogenic isotopic carbon signature masks the hydrogen abiotic origin ^124– 127^. For instance microbial communities relying on a geologic supply of H _2_ have been identified in Precambrian cratons where ancient waters trapped in deep fractures that undergo radiolysis ^128^. Given the natural carbon dioxide abundance in the area ^69, 71, 109^ it is possible that H _2_ is consumed in higher depths by microbes ( Equation 3) ^129^ and thus sustain rock supported ecosystems ^130^ to produce methane and thus the hydrogens abiotic origin is hindered.

$$\text{C}{\text{O}}_{2}+4{\text{H}}_{2}\to \text{C}{\text{H}}_{4}+2{\text{H}}_{2}\text{O}\;(\text{Hydrogenotrophic}\;\text{Methanogenesis})\;(Equation 3)$$

In such a case methane isotopes alone can be misleading about the hydrogen origin and additional tracers such as helium, clumped isotopes ^131^, dissolved hydrogen monitoring will need to be taken into account. Microbial SO _4_^2−^ reduction to H _2_S is a process that occurs in anaerobic environments ( Equation 4) in high depths including lacustrine sedimens and groundwater ^132^ that can also alter the hydrogen’s abiotic origin.

$$\text{S}{\text{O}}_{4}^{2-}+4{\text{H}}_{2}\to {\text{H}}_{2}\text{S}+2{\text{H}}_{2}\text{O}\;(\text{Hydrogenotrophic}\;\text{Sulphate}\;\text{Reduction})\;(Equation 4)$$

Thus, sulphur isotopes will need to be investigated to detect an abiotic hydrogen source. Therefore to properly understand the origins of the hydrogen and methane in the area a combined geochemical–microbiological approach is needed. The afforementioned findings and discussion beg for further investigation, particularly dedicated gas flux monitoring, mineralogical sampling, and structural mapping to delineate potential H _2_-prone zones and assess their economic relevance. This preliminary study sheds light on the complex but dynamically evolving systems that dominate the Mesohellenic and Florina basins. Whereas hydrogen and helium exist in economically viable quantities in the area, the question remains to be answered. Still, the current fluid generation and flow mechanisms existing in the Mesohellenic and Florina basin can shed light and provide analogues for exploration and discovery of economic accumulations.

It is well known that hydrogen seepage on the surface is not constant and may change the point of escape over time or flow rate ^133^ while sampling in water comes with its drawbacks ^118– 123^. Thus, future work should focus on data collection from Katakali, Kivotos, Tropeouhos and Neos Kafkasos over a long period of time rather than being spontaneous. Since the data collection has been conducted on water samples rather than in soil, it is suggested that the aforementioned sites be investigated with a handheld GA5000 or similar over a period of six months or a year on a weekly basis. The day of the visit, the operator should collect at least 6 hourly readings of He or Hydrogen. Should the operator register hydrogen readings, water samples with vials should be taken for the following analysis:

1. ICP for a chemical suite analysis
2. He
3. H _2_
4. CH _4_
5. CO _2_
6. Helium isotopes ( ^3^He, ^4^He)
7. Deuterium of hydrogen (δ ^2^H-H _2_)
8. Deuterium of methane (δ ^2^H-CH _4_)
9. Deuterium of water (δ ^2^H-H _2_O)
10. Oxygen-18 of carbon dioxide (δ ^18^O-CO ^2^)
11. Oxygen-18 of water (δ ^18^O - H _2_O)
12. Carbon-13 of methane (δ ^13^C-CH _4_)
13. Carbon-13 of carbon dioxide (δ1 ^8^O-CO _2_)
14. Carbon-13 of DIC (δ ^13^C-DIC)
15. Carbon-14 of DIC ( ^14^C-DIC)
16. Sulphur isotopes (δ ^34^S)
17. Clumped isotopes (¹³CH _3_D, ^12^CH _2_D _2_)

To fully understand the local system, it is necessary to sample precipitation and surface water, in addition to groundwater ^85^. If any of the sites provide satisfactory results, then, provided that the appropriate permits are granted, permanent equipment should be installed in the springs to continuously monitor for hydrogen and helium. Further to this, a full exploration program with geological mapping and targeted geological soil ^22– 124^ and water sampling with intrusive investigation should then take place.

## Ethics and consent

Ethical approval and consent were not required.

## Data Availability

Zenodo: https://doi.org/10.5281/zenodo.16914636 ^84^ This project contains the following underlying data: Water_samples_1stround_100ml Water_samples_2ndround_250ml Water_samples_3rdround_100ml Water_samples_4thround_100ml Field_Dataentry_form_1st round Field_Dataentry_form_2nd round Field_Dataentry_form_3rd round Field_Dataentry_form_4th round Assigned codes_1st-3rd rounds Assigned codes_4th round Geochemical analysis_1st round Geochemical analysis_2nd round Geochemical analysis_3rd round Geochemical analysis_4th round Stratigraphy Data are available under the terms of the Creative Commons Attribution 4.0 International license (CC-BY 4.0).
