# Investigative research for occurrences of hydrogen, helium, methane and carbon dioxide in West Macedonia: linking geological reservoirs to subsurface gas generation and migration

**DOI:** 10.12688/openreseurope.21180.2

**Published:** 2025-12-15

**Authors:** Pavlos Tyrologou, Nikolaos Koukouzas, Nazaré Couto, Christos L. Stergiou, Júlio Carneiro

**Affiliations:** 1 Chemical Process and Energy Resources Institute (CPERI), Centre for Research and Technology Hellas (CERTH), Marousi, Egialias 52, 151 25,, Greece; 2 Center for Environmental and Sustainability Research & CHANGE - Global Change and Sustainability Institute, NOVA School of Science and Technology, NOVA University, Lisbon, Campus de Caparica, 2829-516 Caparica, Portugal; 3 ICT/IIFA, Geosciences Department, Universidade de Évora, Évora, R. Romão Ramalho, 7000-671, Portugal

**Keywords:** helium, methane, natural hydrogen, geological storage, gas migration, isotope, geological reservoirs

## Abstract

**Background:**

Climate change, the need for energy optimisation and higher efficiency have led to the adoption of the Paris Agreement as a response to the urge for action. The European Union has translated the aforementioned into an action framework via the Green Deal and the EU taxonomy regulation. These have initiated a series of research actions under the EU Horizon programme. Part of this research is based on carbon dioxide capture and geological storage, such as the Pilot Strategy, and hydrogen storage, such as the HyStorIES, both Horizon 2020 project. A focused hydrogeochemical survey as part of a larger mapping survey was conducted in West Macedonia to identify a potentially suitable location for geological reservoirs, gas sources and gas migration routes based on previous research. Gases investigated were hydrogen, helium, methane and carbon dioxide. The study involved isotopes to identify the source of gases and thus provide clues for generation and migration routes.

**Methods:**

The investigation presented in this study deployed sequential spring and borehole water sampling for geochemical analysis of trace elements and gas analysis for hydrogen, helium, methane and carbon dioxide to identify and characterise gaseous geological reservoirs. The investigation extended into isotope studies for d 13C TDC, d 13C CH4, dD CH4, δD H20, δ 18Ο Η2Ο.

**Results:**

The analysis provided evidence for the existence of helium, biogenic methane, carbon dioxide and traces of hydrogen that need to be further investigated for validation and better understanding of the gas generation and migration routes.

**Conclusions:**

The data suggests the existence of helium, methane, carbon dioxide and validated trace concentrations of hydrogen from previous studies in the wider area. Isotopic analysis provides strong evidence for biotic generation of methane, whereas helium comes from a deeper source. This preliminary investigation indicates the existence of multiple gas generation and migration mechanisms and paves the way for further research.

## Plain language summary

As our humanity evolves, we realise that our current way of converting energy is not sustainable, it creates geopolitical tensions, competition for resources and results in climate change. The future of the current generation and future ones is at stake. We have already experienced the results of climate change and its direct impacts on the food production chain, as well as water scarcity, manifesting themselves in cost and price increases in developed countries, whereas in underdeveloped and developing countries, the situation is difficult to estimate, but it is even worse. Our research focuses on alternative ways of managing energy and carbon dioxide storage to battle climate change for a just society. In addition, we investigate alternative localised natural sources of energy such as natural hydrogen and methane to support energy independence for prosperous societies.

## Introduction

The relatively recent political change in tackling the climate change issue has been expressed with the Paris Agreement ^1^ on a global scale. The Green Deal ^2^ in the European Union has signaled the way forward for the energy transition and decarbonisation of industry. Carbon capture and storage ^3, 4^ technology has provided an intermediate solution to keep business as usual. Underground hydrogen storage and geological carbon storage have been developed almost in tandem, and the latter has drawn on substantial technical knowledge from the former. As the scientific society strives to find solutions the aforementioned technologies have serendipitously led to discoveries and the conceptualisation of natural hydrogen resources. In Greece, West Macedonia, HyStorIES and PilotSTRATEGY focuses on the deep saline aquifers, porous rock formations filled with brine several kilometres below ground, which promise a large capacity for storing CO _2_ captured from industrial clusters or hydrogen. However, during the investigation, an additional research area related to hydrogen occurrences was identified.

The concept of natural hydrogen, although not new, still remains in its infancy with only a handful of researchers together and some pioneer industries actively pursuing the subject to identify large and constant sources that will be able to support a reliable energy transition ^5– 9^. Based on stochastic modelling earth is estimated to be capable of producing some 5.6 × 10 ^6^ Mt of hydrogen. Most of it is unrecoverable because its accumulation is too deep, too small in volume, dispersed, or too far away offshore. Still, if a small portion of this, such as 10 ^5^ Mt, can be recovered, it would be able to supply the global needs for 200 years with net-zero carbon emissions ^10^. This will provide enough time to achieve nuclear fusion ^11^ and make the next revolutionary step in human civilisation history based on the Kardashev scale ^12^.

Promising results for natural hydrogen have already been provided in Mali ^13^, in Namibia ^14^, in Colombia ^15^, in Australia ^16^, and in the Balkans in Albania and Kosovo ^17^. Daskalopoulou *et al.*, 2018 ^18^, have also reported traces of natural hydrogen in West Macedonia together with helium and methane. The understanding of natural hydrogen generation mechanisms is still under development, but in general include:

1) Contact of water with reducing agents in the mantle ^19– 21^.
2) Reaction of water with ultrabasic rocks, serpentinization ^22, 23^- type reactions involving the reduction of water by iron-rich minerals have generally been regarded as requiring high temperatures (>~200°C) ^24^, although this has been recently challenged with observation at lower temperature conditions (<200°C) ^25, 26^.
3) Reduction of water by iron-rich minerals in banded iron formations ^27– 29^ or biotite-rich granites ^30^.
4) High–thermal maturity organic-rich rocks ^31, 32^.
5) Decomposition of organic matter ^19^ of NH _3_ to N and H _2_ at high temperature (up to 600°C).
6) Natural electrolysis of water ^33^.
7) Natural radiolysis of water ^16, 19, 34^.
8) Mantle degassing ^19, 35^.
9) Biological activity ^22^ via microbes in soils and coal beds.
10) Cataclasis ^19, 20, 36^ during earthquakes.

Hydrogen is highly reactive and susceptible to redox conditions, making long-term preservation during migration challenging ^10^. In addition, microbial communities are capable of utilising and producing hydrogen that exists in the subsurface ^37^. Thus, understanding the mechanisms of hydrogen generation, seepage systems ^38^, residence time in geological reservoirs, biotic and abiotic loss are essential for guiding its exploration and sustainable production. Exploration is currently based on research and development of natural hydrogen conceptual geological models that will serve as geological analogues to identify prospective place for exploitation.

Past preliminary investigations in West Macedonia, Greece, have detected the presence of dissolved helium and traces of hydrogen in groundwater and spring emissions, alongside significant methane and carbon dioxide ^18^. While geochemical and isotopic evidence indicated that the methane is largely biogenic in origin, helium and hydrogen when occur together there are high chances that are derived from abiotic process ^39^.

Based on these past findings, a scientific hypothesis is formulated on the grounds that West Macedonia, Greece, hosts active geological processes based on serpentinisation of ultramafic rocks and radiogenic decay that are capable of generating natural hydrogen and helium. These gases may migrate along fault-controlled pathways and where suitable porous and permeable geological formations and conditions exist, could accumulate in subsurface compartments.

To test the scientific hypothesis the following objectives are investigated:

1. Confirm and map gas occurrences especially hydrogen and helium by targeting sampling in West Macedonia.
2. Determine biotic versus abiotic gas origins using isotope evidence.
3. Investigate gas migration pathways such as faults ^38^ via structural and hydrogeological analysis of the study area.
4. Characterise geological settings and rock reservoirs by assessing the potential of geological formations to generate, host and trap hydrogen and helium.
5. Assess resources potential and energy implications by estimating the natural hydrogen and helium volume available for use.

The geological conceptual model to be used as an analogue for testing the aforementioned hypothesis is as follows. Areas with potential for natural hydrogen exploration/exploitation require a source of sufficient hydrogen generation, a mechanism for vertical and lateral fluid migration, porous rock reservoirs for storage, and low permeability rocks (seals) to prevent leakage and allow for economically viable reserves. An additional component that relates to preservation needs to be considered due to hydrogen’s high reactivity and microbial consumption. Natural hydrogen is generated in high depths, while it is consumed in shallow ones ^40^. Detection of abiotic methane, brucite and carbonates is a useful indicator for the presence of natural hydrogen ^14, 41^. These indicators may be found together or individually in the field with closely related ultrabasic rocks. Helium is also generated by decay of radiogenic nuclides in similar rocks and can be related to natural hydrogen ^40^.

In general, the presence of talc indicates higher silica activity in serpentinising systems, which diverts reactions away from magnetite formation and consequently diminishes the capacity to generate H _2_. In contrast, low silica activity favours H _2_ production ^24^. Indicators of low-silica, H _2_-generating conditions include the formation of abundant magnetite as well as brucite, which both are formed during serpentinisation reactions that release molecular hydrogen ^42– 44^. Serpentinisation often produces hyperalkaline fluids with pH>10. These fluids are rich in calcium Ca ^2+^ and OH ^-^ but depleted in silica and Mg ^2+^ ^45^, reflecting the hydration of olivine and pyroxenes in a deeper and closed system ^44^.

Hyperalkaline springs found in ophiolites are related to serpentinisation ^17^ via the following general chemical Equation 1 where olivine is the source rock of Fe ^2+^ that oxidises to magnetite (Fe ^3+^) ^33^:


$$\text{F}{\text{e}}^{2+}+{\text{H}}_{2}\text{O}\overset{\text{yields}}{\to }\text{F}{\text{e}}_{3}+\frac{1}{2}{\text{H}}_{2}+\text{O}{\text{H}}^{-}\;(Equation 1)$$

These hyperalkaline springs are typically geologically linked with faults, which act as upwelling pathways from deep aquifers ^45^.

In terms of the geological setting of interest, ophiolites that are thrusted and overlain by carbonate rocks, can provide ample quantities of CO _2,_ leading to the following very slow reaction ( Equation 2) but nonetheless important over geological time:


$$\text{C}{\text{O}}_{2}+4{\text{H}}_{2}=\text{C}{\text{H}}_{4}+2{\text{H}}_{2}\;\mathrm{with}\;\text{T}<100{}^{\circ}\text{C}\;{\left(\text{Sabatier}\;\text{reaction}\right)}^{26}\;(Equation 2)$$

whereas thrust and other related faults allow for fluid percolation from deep aquifers below or from rainwater above to enable the above reactions to take place. Reaction described in Equation 2 can be catalysed by magnetite (Fe _3_O _4_), chromite (FeCr _2_O _4_) and awaruite (Ni _3_Fe), at temperatures above 200°C ^44, 46^.

## Supra regional geological context

Before focus on West Macedonia, Greece, a description of the regional Balkan geological setting with relevance to natural hydrogen research is provided for the reader’s benefit. Research conducted by Levy *et al.*, 2023 ^17^ has identified hot spots of hydrogen occurrences in Albania and Kosova with laboratory water sample analysis from natural springs against C and H isotopes of CH _4_ and H _2_. Four springs out of 21 sites provided strong evidence for H _2_ occurrence. These recent finding in Albania, that are further enhanced by the finding of Truche *et al.* 2024 ^47^, given the geological proximity and affinity to North-West Greece, suggests that similar results may appear in Greece in the Mesohellenic basin. The Mirdita ophiolite in Albania presents interest and a potential analogue as it is located in the Albanides mountain belt that is a segment of the Dinarides–Albanides–Hellenides orogen. It is bounded on the east by the Pelagonian block and on the west by the Krasta, Cukali, and Kruja zones. The latter is well-known for its geothermal potential due to thrust faults that allow hot fluids percolation. The easter and particularly western massifs of the Mirdita ophiolite are mantle domes of harzburgite (peridotite composed of olivine and orthopyroxene and small amounts of chromium-rich spinel) capped by mylonitic plagioclase-amphibole peridotite of 400 m thickness ^48^. The northeastern part of the ophiolite complex can reach 14 km in thickness, whereas the west and southeast are estimated to be 2 km thick ^17^. In Kosova, the ophiolites of the West Vardar zone part of the Dinarides are in continuity of Mirdita ophiolite. Of similar interest is the central ophiolite of Kosova that has hydrothermally metamorphosed, leading to the economic precipitation of magnesite ^17^. The subsequent paragraphs provide the Greek regional geological setting.

The Othrys ophiolite complex in central Greece, specifically within the Dinaric–Hellenic ophiolite belt and lies uncomfortably into the Pelagonian carbonate platform. It represents an obduction event where an oceanic lithosphere has been thrust onto continental crust. These are complemented by dunite forming thin layers, a few cm to a few meters thick, intercalated in the harzburgite. A remnant doleritic sheeted-dyke complex is tectonically overlain by peridotites. Whitish rodingite dykes are found in serpentinised harzburgites very close to the Archani spring ^26^. The peridotites are in tectonic contact with an ophiolitic mélange, which in turn tectonically overlies a Late Cretaceous-Tertiary flysch. An active fault zone, along the Leontari-Anavra line, is found north of Ekkara, at the south border of the Thessaly Plain ^26, 44, 49^. Tsikouras *et al*. (2013) ^49^ and Etiope *et al.* (2013) ^44^ investigated 21 ophiolitic springs in the serpentinised Othrys ophiolite. Four hyperalkaline springs (pH 10.7–11.3) with calcium-rich (Ca–OH) waters indicated the presence of methane and hydrogen. These springs, one in Archani and three in Ekkara, are located along creeks and tectonically related to faults that are directly linked to the main regional active fault system. An estimation revealed that collectively the springs produce some 100 kg/year of methane ^44^. The remaining 17 springs, which had pH<8.7 values, did not show any detectable CH _4_ or H _2_.

The water spring isotope chemical analysis of CH _4_ has revealed that the origin of the methane is not biotic. Thus, in hyperalkaline high silica fluids can positively influence the production of H _2_ whereas low silica fluids may result in low H _2_ production. Othrys springs albeit hydrogen positive, still are releasing low amounts of H _2_ compared to other similar places ^26, 44, 49^. This can be attributed to:

(i) normal hydrogen generation due to interaction with low-silica fluids alone, followed by consumption through CO _2_ hydrogenation and/or loss to the surface as water degasses. Note that H _2_ solubility is much less compared to CH _4_, by about an order of magnitude.
(ii) direct methanation processes involving both low- and high-silica fluids ^10, 44, 49^. High-silica activity promotes the formation of talc or other silica-rich phases, which consumes Mg and reduces the production of brucite (Mg(OH) _2_, thereby lowering H _2_ generation.
(iii) Microbial activity that consumes hydrogen.
(iv) Τemperature decrease or modiﬁcations in water–rock ratios ^44^

Daskalopoulou *et al.*, 2018 ^18^ from their research in West Macedonia, Greece, and particularly in Katakali (Mesohellenic Basin) and Mesohori (Florina Basin), idenitified traces of helium and hydrogen with significant values of methane. Katakali provided 4 ppm He, 11 ppm H _2_ and 880.000 ppm CH _4_. The Mesohori sample registered 4 ppm He, 1.5 ppm H _2_ and 479 ppm CH _4_. These values albeit low are indicative and worth investigating to understand the underlying fluid generation and migration.

## Regional geological setting for Mesohellenic and Florina basin, West Macedonia, Greece

**Figure 1.**
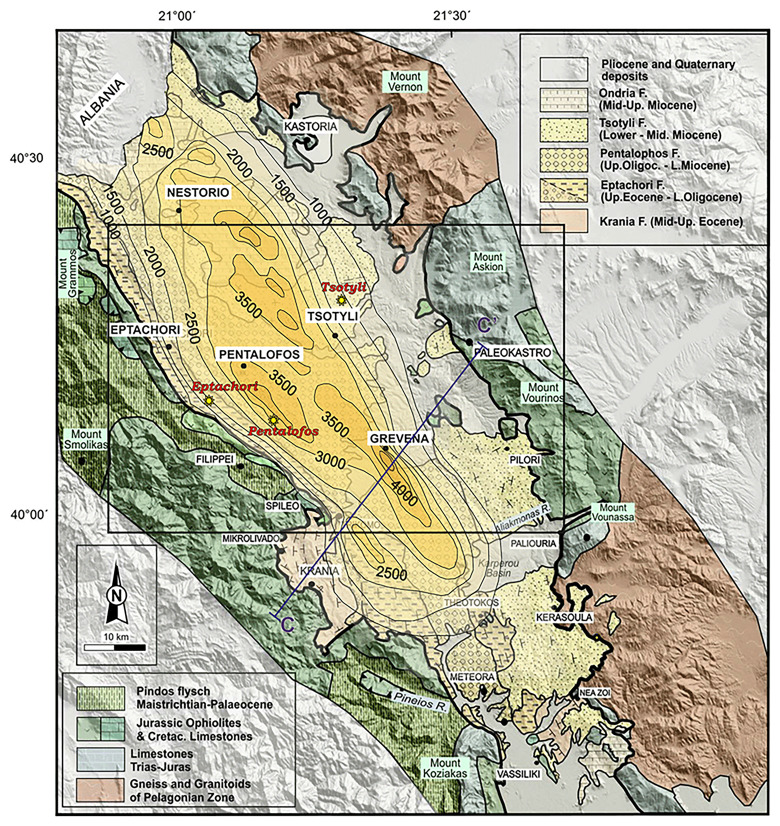
Modified Geological map and isodepths of the basement rocks of the Mesohellenic Basin ^61^, licence: CC-BY 4.0.

Based on the Daskalopoulou *et al.*, 2018 ^18^ findings further research on natural hydrogen was ensued on the Mesohellenic and Florina basin. The geological setting for both basins is given further below. The Mesohellenic Basin (MHB), depicted in Figure 1, extends over 200 km in a NNW-SSE direction, is 20 to 40 km wide and has a thickness of 4500 metres. It is a late-orogenic, molassic-type basin that developed from the Middle Eocene to Middle Miocene in northwestern Greece and southern Albania ^50^. It lies along the suture between the Apulian plate and the Pelagonian nappe pile and marks the boundary between the Internal and External Hellenides ^51– 53^.

**Figure 2.**
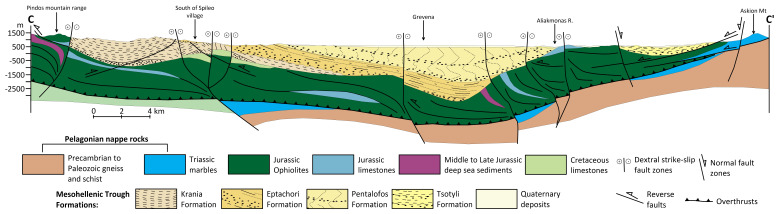
Modified geological cross-section of Mesohellenic Basin ^61^, licence: CC-BY 4.0.

MHB is a favourable area with a potential for hydrogen accumulations associated with the long-distance lateral flow migration that commonly occurs in sedimentary basins. Long-distance lateral migration is defined as migration across map distances of up to 250 km ^40^. Ophiolites ( Figure 2) are abundant in the area, offering promising potential as a source rock for natural hydrogen through serpentinization. The underlying Triassic and Jurassic limestones can contribute significant quantities of CO _2_, mobilised by migratory fluids that percolate vertically and laterally through the rock mass because of the prevailing tectonic setting and associated strain.

The Jurassic oceanic lithosphere was obducted during the closing of the Mesozoic Tethys Ocean, forming the Vourinos and Pindos ophiolites that are exposed along the edges of the MHB ^52, 54^. The Vourinos ophiolite is a well-preserved Penrose-type section made up of pillow lavas, sheeted dikes, a thick ultramafic-mafic cumulate sequence, and mantle peridotites. Hydrothermal activity during seafloor spreading is indicated by features including epidotized dikes, sulfide mineralisation, and jaspers ^55^. A continuous magnetic anomaly beneath the Mesohellenic Trough suggests a common ophiolitic root, supporting a shared tectonic origin ^51, 55^.

The basin formed atop westward-emplaced Tethyan ophiolites following their final emplacement between Middle and Late Eocene during the Alpine orogeny, with subsidence driven by crustal loading and flexure ^50, 56^. The stratigraphy of the MHB is subdivided into seven major lithostratigraphic units ^57– 60^. These include: 1) the Middle-Upper Eocene Krania Formation (Fm) incorporating conglomerates and olistoliths (lower sequence) and turbiditic beds (upper sequence) with a thickness of 1500 m, 2) the Oligocene Eptachorion Fm with silty marls and very-fine sandstone beds overlying thick conglomerates (~1000 m in thickness), 3) the Upper Oligocene-Lower Miocene Taliaros/Tsarnos Fm and the equivalent Pentalofos Fm, showing sequences from sandstones to conglomerates (~2500 m in thickness), 4) the Lower to Middle Miocene Tsotyli Fm with marl-sandstone alternations in the north and conglomerates in the south (~600 m in thickness), and 5) the Ondria Fm and the equivalent Orlias Fm (Lower to Middle Miocene) comprising fossiliferous limestones, marls, and sandstones (≥350 m in thickness). These ΜΗB thick sedimentary strata, as a geological reservoir, offer the benefit of (1) sufficient burial to compact rocks to low porosity and (2) a higher likelihood of one or more potential sealing formations throughout the stratigraphic sequence ^40^. Tyrologou *et al.* 2023 have confirmed with field surveys and laboratory investigation the existence of favourable rock sealing conditions related to the Pentalofos, Eptachori and Tsotyli sedimentary formations ^61^ due to their low porosity and permeability values.

Major tectonic unconformities define the bases of the Krania, Eptachorion, and Tsotyli Formations. Lithologies within these formations include conglomerates of fan-delta and alluvial fan origin, turbiditic sandstones and shales, as well as deltaic, floodplain, and shelf sandstones and siltstones ^57– 59^. These sediments show a general coarsening trend from north to south and were deposited during a progressive shallowing of the basin, with a maximum sedimentary thickness reaching 4.5 km near Grevena ^51, 62^. These sedimentary facies hold potential as porous media capable of economically accumulating hydrogen while being protected from overlain low permeability rocks described previously. Upper Miocene unconformably overlies the molassic sequence to Quaternary deposits.

The Vourinos ophiolitic complex within the MHB ^63^ is the most profound potential source for natural hydrogen. The Theopetra-Theotokos Structure in the southern portion of the basin exposes the basement ophiolitic and carbonate units and runs almost parallel to the main basin axis ^53, 60^. A complex tectonic development influenced by both brittle and semi-ductile deformation occurred in the basin. Along NW-SE to NNW-SSE faults, dextral strike-slip tectonics dominated the Early to Late Oligocene, allowing for lateral basin displacements and subsidence ^64^. The 1995 Grevena-Kozani earthquake sequence ^65, 66^; suggests ongoing reactivation of inherited structures within the MHB.

**Figure 3.**
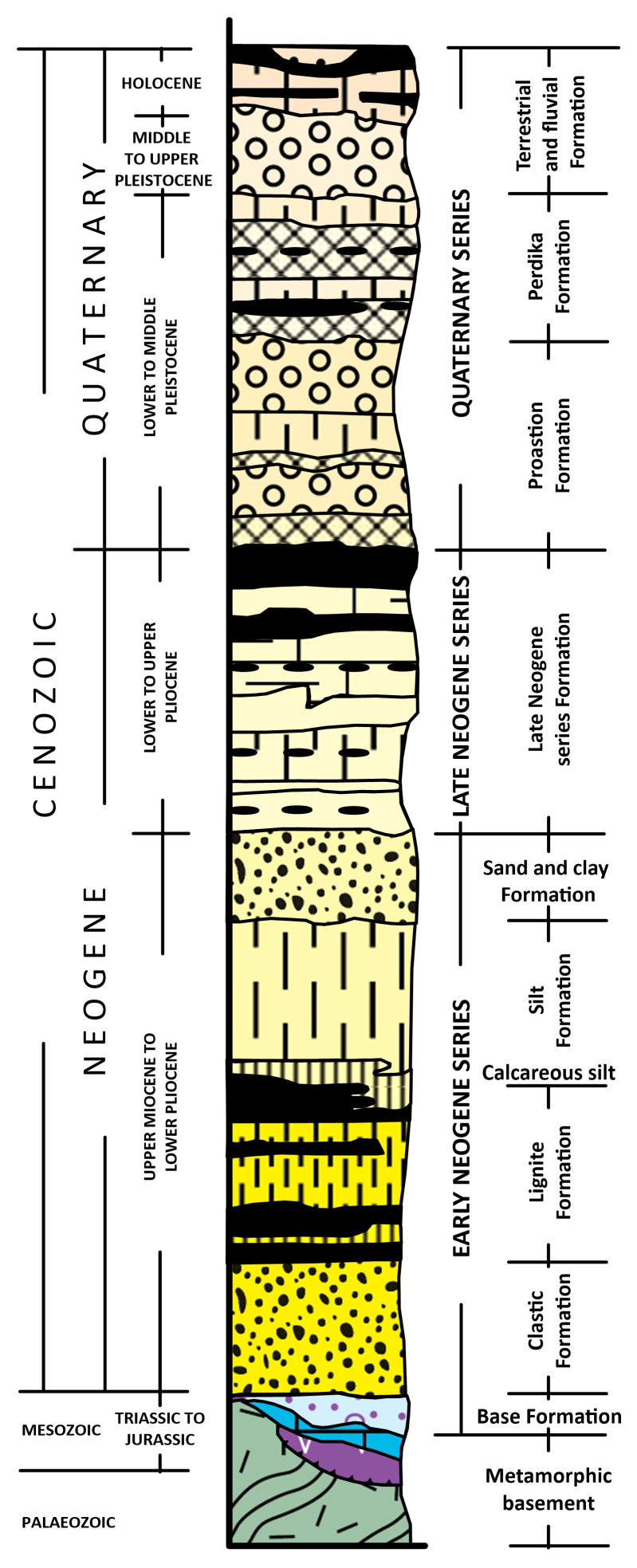
Synthesized stratigraphic column of the Sarantaporos, Ptolemaida-Amynteo -Florina basins, licence: CC-BY 4.0.

The Florina Basin is part of a large NW-SE trending graben system in NW Macedonia, Greece, that formed during the Lower Miocene following the Alpine Orogeny. This graben system (~150 km in length) contains the Florina, Ptolemaida-Amynteo, Kozani-Servia, and Sarandaporo Basins ^67^. Depicted in the synthesised related stratigraphic column in Figure 3, the basement rocks of the basin system include mainly Triassic to Jurassic crystalline limestones, marbles and dolomites, as well as Palaeozoic to Mesozoic schists, phyllites, gneisses, and granites of the Pelagonian Zone ^67^. Locally, ophiolitic rocks with serpentinites and serpentinized peridotites occur ^65^ that present the potential to generate natural hydrogen and can be considered as source rock.

The sedimentary fill of the Florina Basin reaches a total thickness of about 560 m, 21 km width and 17 km length. The basement rocks of the Florina Basin comprise Palaeozoic gneiss, schist and Middle Triassic to Lower Jurassic crystalline limestone, marble and dolomite, which are overlaid by forty-one distinct sedimentary cycles described in three separate formations ^68^.

The Base Formation (Upper Miocene) found first at the bottom (~251–548 m) consists mainly of alternating sand, clay, and conglomerate beds, interpreted as stacked alluvial fan sequences that grade upward into fluvial deposits. A change from active tectonism and a high sediment supply to a quieter regime with less accommodation space is indicated by this vertical transition. CO _2_ accumulations have been found in the fine sand layers of these river deposits, suggesting that they could serve as traps also for natural hydrogen ^69^.

The overlying Vevi Formation (Upper Miocene – Low Pliocene, ~25.1–124 m) records a lacustrine depositional system characterised by 21 distinct sedimentary cycles, where marl, clay and sand dominate. Several sedimentary cycles are capped by lignite seams with a cumulative thickness of up to 6.6 m. These lignite deposits can also generate natural hydrogen ^19, 70^.

The Lophon Formation (0–124 m) is the uppermost unit and represents a return to fluviolacustrine sedimentation during the Early Pleistocene ^67^. Lithology is composed of sand, clay, and silt found as beds and marly limestone as lenses. Cross-bedded conglomerates and sands appear in the upper parts of the stratigraphy. Lophon Formation is distinguished by coarser fluvial interbeds interspersed with fine-grained lacustrine clays and silts ^67^. This change indicates a decrease in accommodation space brought on by either increased sediment influx or decreased subsidence, which is probably related to regional elevation and the Quaternary fragmentation of the Florina-Ptolemais-Servia Basins system (Steenbrink *et al.* 2006).

The sedimentary architecture of the Florina Basin supports the presence of natural gas trapping systems ^71^. The river sand deposits of the Base Formation have been shown to naturally accumulate CO _2_, providing a useful analogy for subsurface storage behaviour. With alternating permeable sands and impermeable layers like clays and lignites, the lithological variability of the basin offers efficient natural trapping mechanisms that hold natural CO _2_ and may also be able to hold natural hydrogen accumulation. Hydrogeochemical analyses show that groundwater quality in regions impacted by natural CO _2_ seepage has not significantly declined ^71^ indicating the existence of low permeable rocks in the vicinity exhibiting good seal capabilities.

Unlike CO _2_, which is soluble and easily immobilised by dissolution and mineral reactions ^72^, H _2_ is far more mobile and weakly soluble ^24, 73^. Any natural H _2_ detected at the surface should not be interpreted as seal failure but rather as evidence of active deep generative zones with focused leakage pathways. Thus, in this case, the CO _2_ preservation demonstrates sealing capacity, whereas H _2_ seepage may reflect deeper accumulation.

The CO _2_ emissions at Florina are ascribed to the regional Quaternary volcanism, and are primarily of crustal origin, including also a restricted mantle input, while NE-SW trending faults serve as fluid migration conduits ^74, 75^. Between 2012 and 2014, the Florina region experienced a significant rise in micro-seismicity, with over 2000 events documented and an Mw 4.1 main shock in February 2013. With certain episodes displaying migration patterns suggestive of pore-fluid pressure diffusion, seismicity seemed to be episodic and clustered. Deep CO _2_ fluids may play a part in fault zone reactivation, as evidenced by shear-wave splitting investigations that showed anisotropy compatible with the local stress field and fault orientations ^76– 78^.

## Local geology setting of promising sites for natural hydrogen

Based on collected data from a) previous research conducted by Daskalopoulou *et al.*, 2018 ^18^ b) observations investigation in the field using anecdotal data, c) from publicly available media of water springs with the elevated gas yield, the authors considered the local geology and concluded that the springs presented in Table 1 are promising places for an initial investigation related to natural hydrogen.

**Table 1.** Locations of water springs observed and suspected with a high possibility of natural hydrogen.

Name	N (WGS84)	E (WGS84)	Χ (Greek Grid, EGSA 87, m)	Y (Greek Grid, EGSA 87, m)	Z (m)
**Katakali**	39° 55' 31,15"	21° 40' 37,5"	301337	4421760	404
**Κivotos**	40° 14' 35,55"	21° 25' 34,62"	280926	4457643	547
**Tropeouhos**	40° 44' 25,45"	21° 26' 23,87"	283693	4512806	695
**Ammohori_1**	40° 46' 39,93"	21° 28' 50,51"	287251	4516854	633
**Ammohori_2**	40° 46' 54,80"	21° 28' 57,69"	287433	4517308	631
**Itea**	40° 50' 1,64"	21° 30' 59,80"	290459	4522988	613
**Marina**	40° 51' 44,29"	21° 29' 34,15"	288543	4526211	595
**Mesohori**	40° 53' 12,92"	21° 29' 16,50"	288208	4528956	589
**Mesocampos**	40° 53' 39,57"	21° 30' 41,36"	290218	4529721	598
**Neos Kafkasos**	40° 54' 14,50"	21° 29' 37,49"	288754	4530841	585

**Figure 4.**
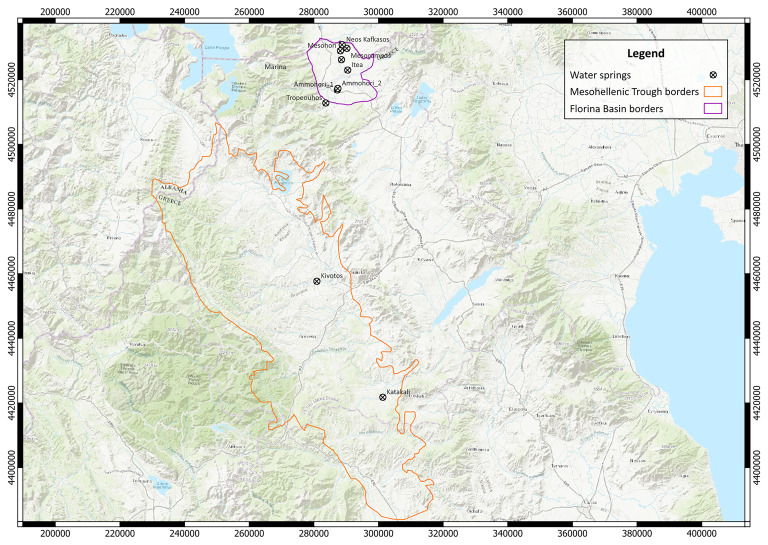
Map with the sample location in EGSA 87 grid system, scale 1:800.000, licence: CC-BY 4.0.

To shed light on potential gas generation and migration mechanisms, the local geology of each water spring and sampling area is summarised based on published geological maps and field observations. Water springs at Katakali and Kivotos emerge through formations of the Mesohellenic Trough, while spring emanations at Tropeouhos, Ammohori, Itea, Marina, Mesohori, Mesocampos and Neos Kafkasos are found at the Florina Basin ( Figure 4).

At Katakali, the near surface layers include Pliocene to Lower Pleistocene marls, sandstones, sandy marls, clays, and conglomerates of the Karperon Basin ^79^. A small lignite layer within the marls near the Sioutsa river indicates local marsh conditions. Near basin margins, these sediments transition into unbedded fluvial-torrential deposits. The lower stratigraphy includes:

1) the Miocene (Upper Aquitanian to Tortonian) Tsotyli Fm of the MHB (<2200 m in thickness). It is composed of ophiolitic conglomerates at the base, gradually transitioning into sandstones, marls, and sandy marls,
2) the Middle to Upper Oligocene Eptachori Fm of the MHB, consisting of ophiolitic conglomerates with lateritic lenses at the base and interbedded sandstones and marls above. This formation lies unconformably over the metamorphic basement ^79^.

The stratigraphy at Kivotos sampling site comprises Quaternary alluvial sediments, such as loose sands and clays, which are underlain by Pliocene to Pleistocene fluvial and lacustrine deposits forming terrace sequences composed of loose conglomerates, blue to greenish clays, sands, and friable sandstones ^80^. The upper parts of this unit are characterised by red clays and conglomerates, suggesting alternating oxidising and reducing conditions in a dynamic depositional setting. The Tsotyli Fm (Aquitanian to Burdigalian) with <500 m in thickness is composed of conglomerates, and intercalated sandstones and clastic limestones, that exhibit rapid lateral facies changes and thinning ^80^. The lowermost unit in the area consists of brecciated Middle to Upper Cretaceous limestones with rudists and marly limestones.

The stratigraphy at Tropeouhos, Ammohori, Itea, Marina, Mesohori, Mesocampos and Neos Kafkasos comprises Neogene sands, clays, and conglomerates that grade downward into massive, whitish-brown to whitish-yellow fossiliferous marly limestones, marls, and clays (<100 metres in thickness) ^68, 81^. The presence of thin lignite and xylite layers in the deeper parts indicates episodes of organic-rich deposition in marshy, low-energy environments. Beneath these Neogene sediments lies the Pelagonian metamorphic basement of Palaeozoic age, composed predominantly of orthogneiss and paragneiss with intercalations of schists in the form of layers and lenses, and minor occurrences of amphibolites ^68, 81^.

Additionally, at Itea and Mesohori, the local stratigraphy includes Pleistocene conglomerates, sandstones, sands, and red clays, indicating deposition in fluvial to alluvial environments under oxidising conditions (<200 metres in thickness) ^68^.

## Materials and methods

Water samples for gas measurements were collected in accordance with the UK Environment Agency Methods for sampling and analysing methane in groundwater: a review of current research and practice ^82^, as well as Capasso and Inguaggiato, 1998 ^37^ and Inguaggiato and Rizzo, 2004 ^83^. The sample collection method deployed is described below.

**Figure 5.**
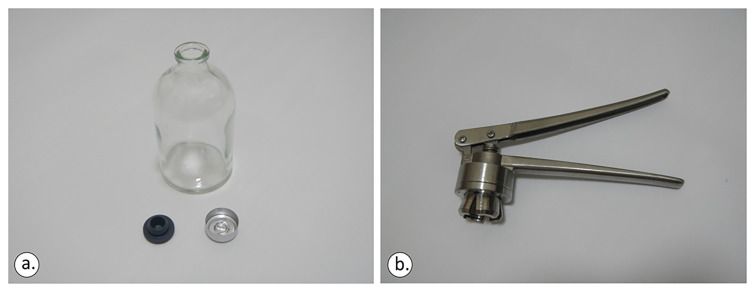
The 100ml crimp-top glass vial, the 20mm rubber closure (lower left) and aluminium cap **(a)** and the standard stainless-steel crimper **(b)**, licence: CC-BY 4.0.

The sampling bottles were 100/125/250 ml vials crimped using a Teflon septum ( Figure 5). Vial size was determined by the target analysis: 100 ml for carbon isotopes, ≥125 ml for noble gas isotopes.

As prerequisite actions, before filling the vials with water sample, the vials were fully filled and rinsed with the same water to be sampled from the spring samples 2 or 3 times to reduce contamination.

Prior to the sampling, the spring water was measured against dissolved O _2_ (mg/L), Conductivity (μS/cm), Temp (°C) and pH. Physico-chemical conditions of the groundwater (pH, temperature, salinity) can contribute to CO _2_ dissolution or release. Differences in He and CO _2_ solubility and reactivity can determine the recorded variations in the He/CO _2_ ratio ^37^. The collection should come in two (2 nr) bottles of 100 mL, or 125 mL per location or sampling area. The first bottle is used for carbon, oxygen, hydrogen and helium isotopes, whereas the other bottle is used for gas chromatography (GC). The exact size of the vials is dictated by the analysis to be performed. Prior conduct with the laboratory to perform the analysis is highly recommended.

In bodies of water such as lakes, pools, seawater, or drainage galleries, as well as any location facilitating direct sample collection beneath the water's surface, it is imperative to submerge and crimp the bottles. This entails placing the bottles underwater, allowing them to fill completely while ensuring that the caps remain submerged at all times to prevent any contact with the atmosphere to avoid any air contamination during the sampling phase. A standard stainless-steel crimper able to cap 8–32 mm aluminium plastic/full aluminium and stainless steel/tight caps was used for sealing the sampling bottles.

**Figure 6.**
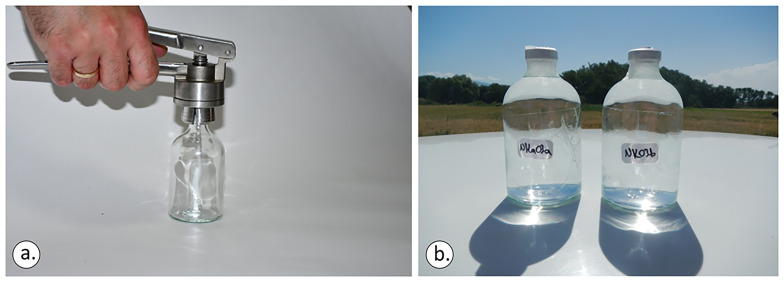
Ensuring that the bottom of the bottle and the mouth of the crimper are levelled before applying force to the crimper’s tongues ( **a**). Sampling vials should be fully filled ( **b**), licence: CC-BY 4.0.

Both the rubber closure and aluminium cap ( Figure 5) were applied and the vials were moved sideways to check if any air bubbles were visible to the naked eye. In any sampling action, it was ensured that the bottom of the bottle and the mouth of the crimper were levelled before applying force to the crimper’s tongues ( Figure 6). Otherwise, the aluminium cap will not be sealed perfectly.

For water sampled from taps, a tube of suitable diameter, crafted from either silicone or Teflon, is required. This tube was inserted into the bottles until reaching the bottom, enabling the water to fill the container from bottom to top. Subsequently, the tube is slowly removed, ensuring the vial is entirely filled up to the upper portion of the bottle neck. Extreme care was taken to eliminate any air bubbles during this process, followed by sealing the vial securely to prevent air contamination. It is recommended that the final procedure be conducted within a cylinder containing an identical sample of water extracted from the tap. This approach ensures the vial is fully submerged upon reaching its capacity, facilitating underwater sealing and crimping with water acting as a barrier to prevent air entrapment.

**Figure 7.**
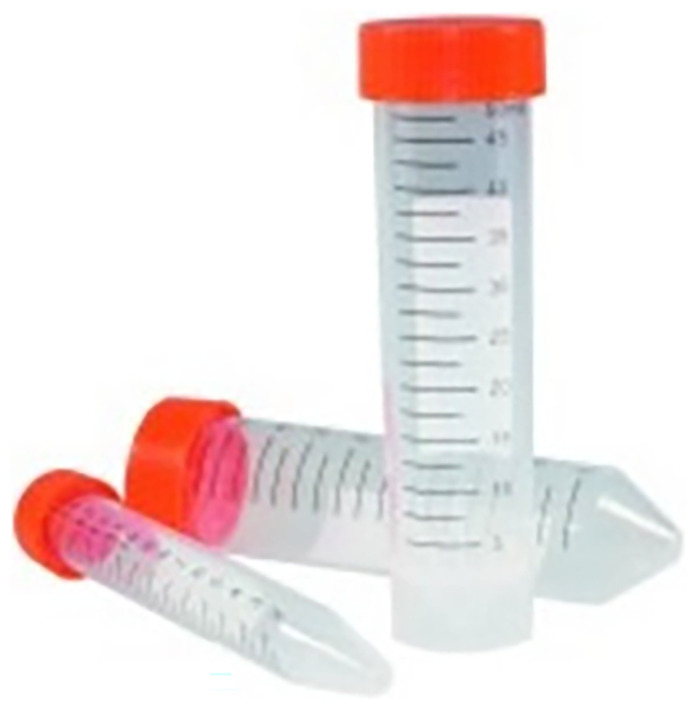
Plastic (Falcon) 50 ml sample filters, license: CC-BY 4.0.

For inductively coupled plasma mass spectrometry (ICP-MS) analysis, employing plastic sterilised (Falcon) 50ml sampler filters ( Figure 7), which have been appropriately acidified beforehand, is deemed sufficient.

After the sampling took place, a visual observation of the existence or nonexistence of bubbles was noted. Samples were appropriately numbered on a pre-agreed sampling system and metadata such as location name, coordinates, time and data of sampling, related geological formation and any other observed data were properly recorded. Recording sample numbers in each sample bottle is vital to minimise analytical problems during the laboratory investigation. All data collected, recorded and subsequently uploaded in SESAR, the System for Earth and Extraterrestrial Sample Registration. Each sample was allocated an International Geo Sample Number (IGSN) to ensure that they are Findable, Accessible, Interoperable, and Reusable (FAIR) ^84^. The IGSN persistent identifier can link each sample with any analyses performed in any laboratory. Published data can be accessed via hyperlinked journals online. All data can be located via online geological data services or the Zenodo platform.

Four sampling campaigns were distributed across different seasons, with collections in summer 15–19 July 2023, spring 21 – 22 April 2024, late spring/early summer 5–6 June 2024, and late autumn/early winter 29 November–3 December 2024, enabling the assessment of potential seasonal effects such as temperature changes, precipitation patterns, organic matter input, and hydrological dynamics. In each case, the samples were shipped to an external accredited laboratory and specifically to the Laboratory for Stable Isotopes, Istituto Nazionale di Geofisica e Vulcanologia Sezione di Palermo in Italy for geochemical laboratory analysis that followed the below provided protocol

Samples were analysed for He, H _2_, O _2_, N _2_, CH _4_ and CO _2_ by gas chromatography (Perkin Elmer Clarus500 equipped with a double Carboxen 1000 packed column system, TCD-FID detectors) using Ar as the gas carrier. TCD detection limit for O _2_, N _2_, and CO is 10–100 μmol/mol (ppm _v_) and for H _2_ and He is 1–10 μmol/mol (ppm _v_). For FID detection limit for CH _4_ is 0.1–1.0 μmol/mol (ppm _v_). Ar was analysed with a Perkin Elmer XL gas chromatograph with MSieve 5A column, TCD detector having He as carrier. Detection limit for Ar is 10–100 ppm and analytical uncertainties are ± 5%. Hydrocarbon analyses were performed with a Shimadzu 14a gas chromatograph equipped with a Flame Ionization Detector (FID) using He as the carrier gas. The analytical error is ≤5% with a minimum detectable quantity of MDQ ≤ 3×10 ^-12^ gC/sec.

Isotope determinations of δ ^18^O _H₂O_/ ^16^O and δD _H₂O_ in water samples were performed using the equilibration technique for oxygen and water reduction (hydrogen production using granular Zn) for hydrogen. Measurements were carried out using a FinniganDelta Plus mass spectrometer (Hydrogen) and an automatic preparation system coupled with an AP 2003 IRMS (Oxygen). The δ ^18^O _H₂O_/ ^16^O and δD _H₂O_ ratios are reported relative to the V-SMOW (Vienna Standard Mean Ocean Water) scale. Isotope ratios are determined by comparing three in-house water standards that have been previously calibrated using IAEA international reference standards such as VSMOW2 and SLAP2 (Standard Light Antarctic Precipitation 2) ^85, 86^. Analytical precision computed as 1σ on ten measurements of the same sample, for each measurement is better than 0.2‰ for δ ^18^O _H₂O_ and 2‰ for δD _H₂O_.

Carbon isotope composition of CO _2_ was determined by using a Thermo Delta Plus XP, coupled with a Thermo TRACE Gas Chromatograph (GC) and a Thermo GC/C III interface. The TRACE GC is equipped with a Poraplot Q (25 m × 0.32 mm) column and uses Helium (5.6) as carrier gas at a constant flow of 0.9 cm ^3/^min. Undesired gas species, such as N _2_, and CH _4_, are vented to the atmosphere using back-flush of He and a Sige valve. The ^13^C/ ^12^C ratios are reported as δ¹³C _CO₂_ values relative to the V-PDB standard (Vienna Pee Dee Belemnite) ^87^. Carbon isotope ratios were determined by comparing three in-house standards δ¹³C _CO₂_ ranging from +0.3 ± 0.1‰ to −28.5 ± 0.3‰ vs V-PDB calibrated using a CO _2_ standard (RM8564 ^88^ ) with known isotopic composition (δ ^13^C _CO₂_ = −10.45 ± 0.04‰ vs V-PDB) and two international standards (NBS 18 and NBS 19 ^87^ ). External precision, computed as 1σ (standard deviation) on ten measurements of the same sample, is 0.1‰. The RM8564, NBS 18 and NBS 19 standards can be obtained from the Terrestrial Environment Radiochemistry Laboratory of the International Atomic Energy Agency ( https://analytical-reference-materials.iaea.org/catalogs, web page accessed on 21/08/2025)

Carbon (δ¹³C _CH₄_) and hydrogen(δD _CH₄_) isotopes of CH _4_, both in free gases and in dissolved gases, were measured using a Thermo TRACE GC interfaced to a Delta Plus XP gas source mass spectrometer and equipped with a Thermo GC/C III (for Carbon) and with GC/TC peripherals (for Hydrogen). The gas chromatograph was equipped with an Rt-Q Plot column (Restek 30 m × 0.32 mm i.d.) and the oven was held at a constant temperature (50 °C for carbon and 40 °C for Hydrogen). The flow rate of carrier gas (He 5.6 grade) was held at a constant flux of 0.8 cm ^3/^min. A split/splitless injector with a split ratio from 10:1 to 80:1 was used for sample introduction, except for diluted samples (CH _4_ concentration lower than 10 mmol/mol) when direct on-column injection was performed.

The inlet system consists of a stainless steel loop with a known volume (50 μl), connected to a two-position six-port Valco® valve. Before the introduction of the sample, a vacuum of 10 ^−2^ mbar measured with an EBRO pressure gauge is ensured by a rotary vane pump. Once CH _4_ was separated from the gas mixture, it was quantitatively converted to CO _2_ by passing through a combustion oven (T = 940 °C) for ^13^C/ ^12^C ratios analysis or to H _2_ by passing it through a reactor set at a temperature of 1440 °C for ^2^H/ ^1^H ratios analysis. Each sample analysis took about 500 s.

The ^13^C/ ^12^C ratios are reported as δ ^13^C _CH₄_ values for the V-P standard and ^2^H/ ^1^H ratios are reported here as δD _CH₄_ values with respect to the V-SMOW standard. Carbon isotope ratios were determined by comparing an in-house standard (δ¹³C _CH₄_ = −49.5 ± 0.2‰) calibrated using four CH _4_ standards (Isometric Instruments) with known isotopic composition (δ¹³C _CH_ ranging from −23.9 ± 0.3‰ to −66.5 ± 0.3‰ vs V-PDB).

Hydrogen isotope ratios were determined by comparing an in-house standard (δ¹³C _CH₄_ = −200 ± 2.0‰) with a CH _4_ standard with known isotopic composition δD _CH₄_ = −186.1 ± 3.0‰ vs V-SMOW). External reproducibility, estimated as 1σ (standard deviation) on ten measurements of the same sample, is 0.2‰ and 2.0‰ for carbon and hydrogen isotopes, respectively. The measured δD _CH₄_ values were normalised using the standards to ensure they were accurately reported on the V-SMOW scale. The estimated external accuracy (or overall measurement uncertainty) for the final reported δD _CH₄_ values is better than ± 2.5‰.

In CO _2_-dominated gases having CH _4_ concentrations lower than 1000 μmol/mol, the analyses of the isotope ratios of methane were carried out in the headspace gas samples collected using pre-evacuated 60 mL glass flasks ﬁlled with 20 mL of a 4 N NaOH solution.

The abundance and isotope composition of He, and the ^4^He/ ^20^Ne ratios, were determined by separately admitting He and Ne into a split flight tube mass spectrometer (Helix SFT). Helium isotope compositions are given as R/RA, where R is the ( ^3^He/ ^4^He) ratio of the sample and RA is the atmospheric ( ^3^He/ ^4^He) ratio (RA = 1.386 × 10 ^−6^). The analytical errors were generally < 1%.

Location names, sampling date and coordinates of all new sampling sites together with raw chemical results can be found as supplementary material at https://doi.org/10.5281/zenodo.16914636 ^89^.

## Results

**Figure 8.**
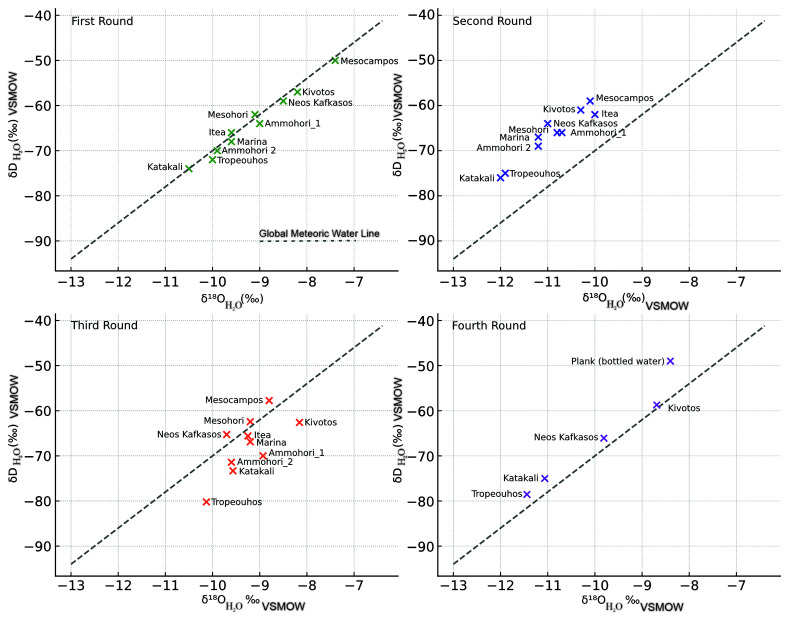
Stable Isotope composition of water samples collected from June 2023 to December 2024 with reference to the Global Meteoric Water Line (GMWL), licence: CC-BY 4.0.

Below are presented the results from all four sampling campaigns, starting with the isotope data analysis ( Table 2– Table 5). As the samples are from groundwater, the Global Meteoric Water line was deployed to analyse the data rather than the Local Meteoric Water line, which relies on precipitation samples only ^90^ ( Figure 8).

**Table 2.** Water samples collected between 15-19/07/2023, laboratory data analysis provided at 06/09/2023, first sample campaign, rounded to two significant figures.

N°	Spring location name	δD _H2O_	δ ^18^Ο _Η2Ο_	Dissolved O _2_ (mg/L)	Conductivity (μS/cm)	Temp (°C)	pH
**1**	Tropeouhos	-72	-10.0	7.9	-	17.5	6.04
**2**	Mesocampos	-50	-7.4	0.19	1686	16	6.06
**3**	Neos Kafkasos	-59	-8.5	0.76	575	14.1	6.08
**4**	Ammohori 1	-64	-9.0	8.3	920	17.5	6.07
**5**	Itea	-66	-9.6	2.73	1017	16.5	6.03
**6**	Mesohori	-62	-9.1	1.09	478	14	6.03
**7**	Ammohori 2	-70	-9.9	7.1	319	20.5	6.01
**8**	Marina	-68	-9.6	6.3	338	25.6	6.04
**9**	Kivotos	-57	-8.2	0.24	850	15.3	5.4
**10**	Katakali	-74	-10.5	0.25	1447	18	6.0

**Table 3.** Water samples collected between 21-22/04/2023, laboratory data analysis provided at 15/05/2024, second sample campaign.

N°	Spring location name	δD _H2O_	δ ^18^Ο _Η2Ο_	Dissolved O _2_ (mg/L)	Conductivity (μS/cm)	Temp (°C)	pH
**1**	**Tropeouhos**	-75.0	-11.9	8.85	1803	14.4	7.5
**2**	**Mesocampos**	-59.0	-10.1	2.59	1984	13.5	5.67
**3**	**Neos Kafkasos**	-64.0	-11.0	1.60	468	14.5	5.42
**4**	**Ammohori 1**	-66.0	-10.7	7.06	971	12	6.49
**5**	**Itea**	-62.0	-10.0	2.91	972	13	5.61
**6**	**Mesohori**	-66.0	-10.8	2.50	450	13	6.5
**7**	**Ammohori 2**	-69.0	-11.2	7.78	318	14.8	5.7
**8**	**Marina**	-67.0	-11.2	8.15	347	14.1	6.29
**9**	**Kivotos**	-61.0	-10.3	1.75	803	14.9	7.25
**10**	**Katakali**	-76.0	-12.0	1.40	1440	16.1	7.5

**Table 4.** Water samples collected between 05-06/06/2024, laboratory data analysis provided at 24/07/2024, third round campaign.

N°	Spring location name	δD _H2O_	δ ^18^Ο _Η2Ο_	Dissolved O _2_ (mg/L)	Conductivity (μS/cm)	Temp (°C)	pH
**1**	Tropeouhos	-80	-10.1	0.78	1694	20.0	7.04
**2**	Mesocampos	-58	-8.8	1.72	1809	20.3	6.01
**3**	Neos Kafkasos	-65	-9.7	1.05	477	17.0	5.83
**4**	Ammohori 1	-70	-8.9	4.87	968	21.0	6.76
**5**	Itea	-66	-9.3	2.63	712	16.1	5.83
**6**	Mesohori	-62	-9.2	2.27	485	16.0	6.22
**7**	Ammohori 2	-71	-9.6	7.33	301	21.4	6.20
**8**	Marina	-67	-9.2	7.00	329	22.9	6.56
**9**	Kivotos	-63	-8.2	1.05	781	18.8	7.65
**10**	Katakali	-73	-9.6	7.22	1468	18.0	8.48

**Table 5.** Water samples collected between 29/11/2024 – 3/12/2024. Laboratory data analysis provided at 06/03/2025, fourth campaign.

N°	Spring location name	δD _H2O_	δ ^18^Ο _Η2Ο_	Dissolved O2 (mg/L)	Conductivity (μS/cm)	Temp (°C)	pH
**1**	**Katakali**	-11.1	-75	0.26	1505	15.1	8.04
**2**	**Kivotos**	-8.6	-58	0.25	855	14.3	7.75
**3**	**Tropeouhos**	-11.5	-78	0.41	1862	14.2	7.12
**4**	**Neos Kafkasos**	-9.8	-66	1.22	487	14.3	6.03
**5**	**Katakali-2**	-11	-75	0.26	1505	15.1	8.04
**6**	**Kivotos-2**	-8.8	-59	0.25	855	14.3	7.75
**7**	**Tropeouhos-2**	-11.4	-79	0.41	1862	14.2	7.12
**8**	**Plank (bottled water)**	-8.4	-49	-	-	-	-

 In all sampling campaigns, the pH measured was either slightly alkaline, with values around 7.5 or slightly acidic and values around 5.4. There was only one exception in the third sample campaign, in the Katakali water sample, which registered an alkaline pH of 8.48.

The ICP chemical analysis of the second round presented in Table 6 revealed that all samples had high detectable levels of strontium (Sr) and barium (Ba) in the scale of μg/L. The Si concentrations varied from 7.7 to 59.6 mg/L, indicating variable rock silicate interaction ^91, 92^.

**Table 6.** ICP chemical analysis of water samples from Kivotos Ammohori 1, Ammohori 2, Itea, Marina, Mesohori, Neos Kafkasos, Katakali, Tropeouhos, Mesocampos locations. All concentrations are reported in μg/L.

Spring location	Li	Be	B	Al	Ti	V	Cr	Mn	Fe	Co	Ni	Cu	Zn	As	Se	Br	Rb	Sr	Mo	Cd	Sn	Sb	Cs	Ba	Tl	Pb	Bi	U	Si
**Kivotos**	8	<0.02	269	4	0.10	0.05	0.19	5	7	0.01	<0.1	<0.1	<0.1	0.02	0.11	466	0.97	591	0.14	<0.01	0.01	0.00	0.01	67	<0.01	0.01	0.01	0.18	15000
**Ammohori 1**	3	0.16	41	1455	87	5	5.01	37	2807	1	7.6	6	177	0.44	1.2	128	4.4	660	0.24	<0.01	0.23	0.03	0.25	89	0.03	4.77	0.02	1.99	18000
**Ammohori 2**	13	0.22	15	7	0.20	0.75	0.86	10	53	0.03	9.7	47	65	0.05	0.27	88	0.31	281	0.12	<0.01	0.02	0.02	0.00	24	<0.01	1.22	<0.01	0.08	27000
**Itea**	3	0.03	37	40	0.15	0.92	0.63	18	22	0.12	3.7	9	30	0.31	3.2	47	0.57	260	1.09	<0.01	0.04	0.04	0.00	61	0.01	0.23	<0.01	6.07	8000
**Marina**	8	0.04	15	25	0.63	2	2.64	0.29	16	0.03	4.1	1	0.34	0.66	0.41	41	0.39	208	0.19	<0.01	<0.01	0.01	0.00	38	<0.01	0.01	<0.01	0.52	26000
**Mesohori**	7	0.02	90	146	11	0.71	0.58	682	247	2	8.1	15	76	0.15	0.37	58	0.99	214	0.18	0.02	6.03	0.18	0.02	54	0.01	7.89	0.05	0.21	11000
**Neos Kafkasos**	8	0.04	60	30	0.12	0.79	0.12	436	10	2.03	7.3	1	4.7	0.44	0.80	44	0.94	260	0.31	<0.01	0.01	0.01	<0.01	99	0.01	0.09	<0.01	0.32	14000
**Katakali**	132	<0.02	933	5	0.41	0.19	0.61	8	221	0.40	9.0	<0.1	<0.1	0.13	0.17	185	1.1	544	0.10	<0.01	<0.01	<0.01	0.02	156	<0.01	<0.01	<0.01	0.00	60000
**Tropeouhos**	407	1.03	7160	92	1.8	0.25	0.23	444	1010	0.30	0.2	<0.1	0.9	1.5	0.33	303	39	902	6	<0.01	<0.01	0.01	31.12	11	<0.01	0.20	<0.01	0.09	17000
**Mesocampos**	5	0.03	268	17	0.34	5	0.47	50	57	1	23.6	21	41	1.7	2.5	362	42	970	3	<0.01	0.06	0.08	0.05	183	0.05	0.31	<0.01	1.17	34000
***error %***	*11*	*8.90*	*3*	*10*	*11*	*7*	*2.30*	*3*	*12*	*2.00*	*12.8*	*14*	*2.6*	*5.1*	*14.3*		*2.2*	*2*	*5*	*12*	*8.20*	*6.70*	*1.70*	*4*	*4.80*	*10.10*	*-*	*2.10*	11000

Regarding the rest of the chemical elements identified Tropeouhos and Ammohori 1 had high levels of Fe (1010 and 2807 μg/L), Mn (37 and 444 μg/L), and Al (92 and 1455 μg/L), respectively. Ammohori 1 also showed elevated Ti (86 μg/L) and Zn (176 μg/L), while Tropeouhos had significantly high concentrations of Li (406 μg/L), B (7160 μg/L), and Br (303 μg/L).

The Mesohori water sample had notable values of 132 μg/L of Al, 682 μg/L of Mn and 247 μg/L of Fe. The Neos Kafkasos water sample showed Mn values of 436 μg/L. The Katakali sample had prominent values of 132 μg/L of Li, 933 μg/L of B, 220 of Fe μg/L, 185 μg/L of Br. Mesokampos had 363 μg/L of Br. All other chemical elements detected from the second water sampling campaign were of considerably lower value.

The geochemical analysis from the third sampling campaign presented in Table 7 revealed further information related to the gas component of the water samples. The Katakali water sample analysis provided values 4.4 ppm of H _2_ coupled with 3704 ppm of CH _4_, suggesting active geochemical processes related to either organic degradation or serpentinisation. The Kivotos sample showed values of 29 ppm of He, 1.7 ppm of H _2_, and 31,400 ppm of CH _4_. The latter consists of anecdotal public observations suggesting strong methane generation potential and possible mantle-derived gas contribution to gas mix phase ^43^. In both Katakali and Kivotos samples, H _2_ was detected at concentrations near the analytical detection limit, confirming its presence but introducing uncertainty in the reported values.

**Table 7.** Gas chemical analysis as a result of the third water sampling campaign, n.a denotes not enough sample quantity to perform the chemical analysis for that specific element.

Spring location	He (ppm)	H _2_ (ppm)	O _2_ (%)	N _2_ (%)	CO (ppm)	CH _4_ (ppm)	CO _2_ (%)	Gas volume (cc/120 cc)	He (×10 ^−4^ cc/L STP)	H _2_ (cc/L STP)	O _2_ (cc/L STP)	N _2_ (cc/LSTP)	CO (×10 ^-6^ cc/L STP)	CH _2_ (×10 ^-6^ cc/L STP)	CO _2_ (cc/L STP)
**KATAKALI**	n.a	4.4	5.22 ×10 ^-2^	18.14	n.a	3704	0.79	6.4	0.0	0.00	0.04	17.0	<LOQ	362.5	8.90
**ΚIVOTOS**	29	1.7	4.41 ×10 ^-2^	19.87	n.a	31400	1.58	6.4	16.4	0.00	0.02	11.3	<LOQ	1778.4	0.90
**TROPEOUHOS**	1403		3.66 ×10 ^-2^	24.81	n.a	89	2.16	6.4	794.6	0.00	0.02	14.0	0.0	5.0	1.20
**AMMOHORI_1**	n.a		1.65	19.02	0.2	1.2	7.08	6.6	0.0	0.00	0.96	11.1	12.5	0.7	4.10
**AMMOHORI_2**	n.a		5.99	19.88	0.2	1.1	7.87	7.2	0.0	0.00	3.82	12.7	13.9	0.7	5.00
**ITEA**	n.a		1.60 ×10 ^-1^	2.19	n.a	4.1	60.68	14.4	0.0	0.00	0.20	2.8	0.0	52.3	77.30
**MARINA**	n.a		4.35	16.34	0.2	0.8	4.13	6.2	0.0	0.00	2.39	9.0	7.3	0.4	2.30
**MESOHORI**	n.a		3.1 ×10 ^-1^	16.42	n.a	9	3.3	6.8	0.0	0.00	0.19	10.0	0.0	54.2	2.00
**MESOCAMPOS**	n.a		7.01 ×10 ^-2^	2.02	n.a	34	60.22	12.4	0.0	0.00	0.08	2.20	0.0	373.1	66.10
**NEOS KAFKASOS**	6	1.9	1.00 ×10 ^-1^	9.48	2.2	n.a	40.01	10	5.3	0.00	0.09	8.40	779	0.0	35.40

The Tropeouhos sample registered notable He levels (1403 ppm), accompanied by low CH _4_ concentrations at 89 ppm. This could be associated with deep-seated helium degassing coupled with some microbial or thermogenic methane input ^93, 94^.

Samples Ammohori_1 and Ammohori_2 registered elevated CO _2_ concentrations of approximately 7–8%, while methane remained low at around 1 ppm. In addition, the Itea sample contained 60.7% CO _2_ and 4 ppm of CH _4_, suggesting a dominant CO _2_ component in the gas phase. This is consistent with the accumulation of natural CO _2_ in the Florina basin in the Tertiary formation and its leakage through fractures and permeable rocks ^71^. The Mesohori sample registered 9 ppm of CH _4_, while Mesokampos contained 34 ppm of CH _4_ along with approximately 60% CO _2_, pointing to variable organic or volcanic degassing influence ^95^. Finally, the Neos Kafkasos sample exhibited 6 ppm of He, 2 ppm of H _2_, and a substantial 40% CO _2_, indicating multi-gas signatures possibly linked to deep fluid migration through faulted rocks ^74, 95^.

Following the results of the third campaign, a fourth one commenced with a focus on four promising locations of Katakali, Kivotos, Tropeouhos and Neos Kafkasos. To further elucidate the origin of the gas phase, the geochemical investigation considered the following analysis:

1. He
2. H _2_
3. CH _4_
4. CO _2_
5. Helium isotopes ( ^3^He, ^4^He)
6. Deuterium of hydrogen (δ ^2^D _H₂_)
7. Deuterium of methane (δ ^2^H _CH₄_)
8. Deuterium of water (δ ^2^H _H₂O_)
9. Oxygen-18 of carbon dioxide (δ ^18^O _CO₂_)
10. Oxygen-18 of water (δ ^18^O _H₂O_)
11. Carbon-13 of methane (δ ^13^C _CH₄_)
12. Carbon-13 of carbon dioxide (δ1 ^8^O _CO₂_)
13. Carbon-13 of DIC (δ ^13^C _DIC_)
14. Carbon-14 of DIC ( ^14^C _DIC_)

This time, a blind sample system with duplicates and blanks was used during the laboratory analysis. The duplicates were two field-independent samples collected as close as possible to the same point in space and time per site. Blanks were simple water table samples included in the analytical batches. All samples, including blanks, were labelled with a coding system unknown to the laboratory analysts (blind sampling system). None of the blind samples were analytical blanks or matrix spikes. The purpose of the duplicate samples was to serve as a quality control measure for analytical consistency, to ensure operator objectivity, and to assess spatial or micro-scale variability within the same hydrogeological system. The water table blanks were included to monitor potential contamination during sample preparation and analysis. They contained no analytes and was processed alongside the regular samples to ensure quality control and identify any background interference.

The Katakali and Katakali-2 samples, collected independently from the same spring, showed good agreement in δ¹³C _CH₄_ values (–69.5‰ vs –69.8‰), which falls within expected analytical precision (±0.2‰). However, the δ²H _CH₄_ values (–251‰ vs –230‰) differed by approximately 21‰, which may reflect small-scale natural heterogeneity at the sampling site or isotopic fractionation during gas release. Similarly, the Kivotos and Kivotos-2 samples showed a 6.0‰ difference in δ¹³C _CH₄_ (–75.5‰ vs –69.5‰) and a 15.0‰ difference in δ²H _CH₄_ (–107‰ vs –92‰), again suggesting site-specific isotopic variability rather than analytical inconsistency.

The duplicate samples Tropeouhos and Tropeouhos-2 demonstrated satisfactory analytical reproducibility in δ¹⁸O _H₂O_ (–9.2‰ vs –9.1‰, a 0.1‰ difference) and δD _H₂O_ (–62‰ vs –60‰, a 2.0‰ difference), further supporting the consistency of the dataset. Chemical concentrations showed strong consistency; Fe (2807 µg/L vs 2750 µg/L), Mn (444 µg/L vs 439 µg/L), Al (1455 µg/L vs 1418 µg/L), and Li (406 µg/L vs 397 µg/L), with negligible deviation between duplicates. On the same note, elevated B (7160 µg/L vs 7095 µg/L), Br (363 µg/L vs 357 µg/L), and Ba (121 µg/L vs 119 µg/L) values confirmed both geochemical signatures and instrumental consistency. For the Neos Kafkasos samples it was not possible to analyse its duplicate.

In all cases, the duplicate samples showed almost similar values. The results of the fourth campaign are presented in Table 8. It should be noted that although all listed chemical elements were analysed, some were either not detected or present in quantities insufficient for further measurements.

**Table 8.** Targeted Gas chemical analysis as a result of the fourth water sampling campaign.

Spring location	He (ppm)	H _2_ (ppm)	O _2_ (%)	N _2_ (%)	CH _4_ (ppm)	CO (ppm)	CO _2_ (%)	Gas volume (cc/120 cc )	He (×10 ^–4^ cc/L STP)	H _2_ (cc/L STP)	O _2_ (cc/L STP)	N _2_ (cc/L STP)	CH _4_ (×10 ^-3^ cc/L STP)	CO (×10 ^-3^ cc/L STP)	CO _2_ (cc/L STP)	total Alk	δ¹³ C- _TDC_ (‰)	δ ^13^C- _CH4_ (‰)	δ D _CH4_ (‰)
**Katakali**	-	-	0.12	4.07	300370		2.33	7.4	0.0	0.0	0.10	4.19	14558.3	0.0	26.90	15.8	13.9	-69.5	-251
**Kivotos**	43	-	0.19	19.08	43000		1.67	6.2	27.9	0.0	0.14	17.63	1833	0.0	19.10	6.3	-14.2	-75.5	-107
**Tropeouhos**	-	-	0.21	22.56	81		2.4	6	0.0	0.0	0.15	20.44	4.0	0.0	27.40	2.00	3.9	-	-
**Neos Kafkasos**	10	-	0.58	8.45	7		49.9	10	9.8	0.0	0.62	10.65	3.3	0.0	587.10	4.2	-1.2	-	-
**Katakali-2**	-	-	0.1	6.08	312300		2.44	7.6	0.0	0.0	0.09	6.37	15440.5	0.0	28.20	15.7	14.4	-69.8	-230
**Kivotos-2**	44	-	0.24	20.04	44100		1.73	5.8	27.0	0.0	0.17	17.81	1903.3	0.0	19.70	6.4	-15.8	-69.5	-92
**Tropeouhos-2**	1.4	-	0.21	22.47	81		2.49	5	0.9	0.0	0.15	20.36	4.04	0.0	28.40	2.1	1.9	-	-
**Plank (bottled water)**	-	-	3.3	19.86	2.7	19.5	0.94	5.6	0.0	0.0	2.26	17.29	0.1	1.8	10.70	4.0	-11.2	-	-

**Figure 9.**
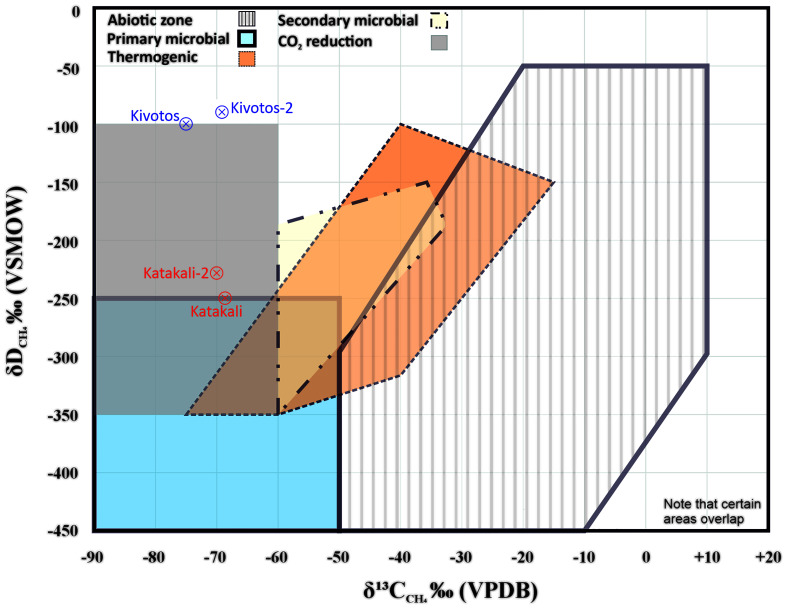
Methane genetic diagram for Kivotos and Katakali samples based on the Milkov and Etiope ^96^, reproduced with CCC permission from Elsevier, modified samples addition and simplified accordingly.

In this final fourth round, no hydrogen gas was registered. However, the Katakali samples showed a mean value of 306335 ppm CH _4_ with δ ^13^C _CH₄_ values around –70‰ and δD _CH₄_ near –230‰. On the same note, the Kivotos samples registered a mean of 43.5 ppm of He coupled with a mean 43550 ppm CH _4_ with δ ^13^C _CH₄_ at –75.5‰ and and δD _CH₄_ near -100‰. Both family of samples (Katakali 1&2 and Kivotos 1&2) are plotted in methane genetic diagram ( Figure 9) based on δ ^13^C _CH₄_ versus δD _CH₄_ to provide an interpretation based on the integrated geological data and geochemical analysis ^96^. The Tropeouhos samples showed a mean of 81 ppm of CH _4_, in this case, the first sample showed no He values, whereas the second sample 1.4 ppm of He. The Neos Kafkasos, although sampled in duplicates, only one sample survived and was analysed. The sample that survived showed a value of 10 ppm of He and 7 ppm of CH _4_. For the Neos Kafakasos sample, there was no δ ^13^C _CH₄_ available. The δ ^13^C _TDC_ was at –1.2‰ and δD _H₂O_ was at –66‰.

An exploratory Spearman rank correlation has been implemented between δ¹³C _CH₄_ and δD _CH₄_ for the four samples with complete isotopic data. The correlation coefficient was low (ρ ≈ 0.10), indicating no monotonic relationship between the two isotopic parameters.

## Discussion and results interpretation

The discussion text is structured on the scientific hypothesis presented in the introduction section against the five objectives formulated for its investigation.

## Hydrochemistry and isotopic evidence supporting gas occurrences

To confirm the presence and occurrences of gas-bearing waters, related to the first objective of the scientific hypothesis presented in the introduction, isotopic and hydrochemical results from all sampling campaigns are considered below.

In the third and fourth sampling campaigns, gas-bearing waters were identified, with helium, hydrogen, methane, and carbon dioxide detected in multiple springs ( Table 7, Table 8). Their presence provides the first line of evidence for active subsurface gas generation and accumulation.

However, several of the hydrogen concentrations measured in this research are in proximity to the lower end of the equipment’s detection range and therefore require careful interpretation. On the same note, the repeated detection of H _2_ at the same sites across independent sampling campaigns (Katakali, Kivotos, Neos Kafkasos), including those conducted by Daskalopoulou *et al.*, 2018 ^18^, and Daskalopoulou, 2017 ^97^, combined with its systematic absence in others, argues against random analytical noise. The spatial consistency of H _2_ with mapped ultramafic lithologies and fault-controlled migration pathways further supports a geological signal rather than an instrumental artefact. In contrast, helium concentrations, particularly the high values recorded at Tropeouhos (up to 1403 ppm) are well above detection limits, reproducible across campaigns, and therefore are considered analytically robust. The combined occurrence of low-level H _2_ with independent geochemical indicators (e.g., alkaline pH, elevated Li and B, reducing Fe–Mn conditions) provides further confidence that even near-detection-limit H _2_ reflects real subsurface processes.

Isotopic composition of δD _H₂O_ and δ¹⁸O _H₂O_ of groundwater provides insights into the origins of the detected gases by shedding light on the recharge conditions, groundwater residence time and mixing with deeper fluids ^83, 90^. Seasonal recharge variations alter hydraulic gradients, pore pressures and redox conditions. These changes can influence the transport and dilution of gases generated at depth,facilitate the upward migration of deep CO₂ and He rich fluids ^98^ and regulate Fe–Mn redox cycling ^99^,

In the first sample campaign ( Table 2, Summer 2023), most samples plot in proximity to the Global Meteoric Water Line ^100, 101^, indicating a meteoric origin with little evaporation enrichment ^102^ ( Figure 8). Katakali and Tropeouhos in the first round ( Table 2) are the most depleted in both isotopes, indicating a colder recharge. Mesocampos and Kivotos are relatively enriched, possibly indicating partial evaporation ^101^. Since the elevations of all sampling locations are relatively similar, altitude differences are unlikely to be the primary factor driving the observed isotopic variability. This original investigation did not provide a distinct chemical signature; however, during the Katakali sample collection, gas bubbles were observed in the water sample. This is due to a decrease in pressure, which causes these dissolved gases to come out of solution and form bubbles.

The second round of data ( Table 3) indicated that Tropeouhos, Mesocampos and Neos Kafkasos had a shift towards isotope depletion, indicating hydrologic change. This can be explained by seasonal input ^103^. The first batch was collected in Summer (June 2023) during a low rainfall season, whereas the second batch was collected in Spring (April 2024) during a high rainfall season. This is consistent with a well-mixed aquifer or steady-state recharge conditions typical of early spring. This sample location above the GMWL ( Figure 8) suggests that precipitation during the winter had sufficient time to infiltrate deeper into the ground and homogenise with groundwater before sampling ^90, 100, 104, 105^.

The third round of samples collected were notably more depleted in δD _H₂O_ and δ ^18^O _H₂O_ and showed higher deviation from GMWL related to rainfall patterns and evaporation, which is consistent with the June sampling period ( Table 4). Still, most of the samples are in proximity to the GMWL, indicating that rainfall is still the prevailing factor ( Figure 8).

The fourth batch of samples collected mostly in Winter (December) plots above the GMWL, suggesting winter recharge from isotopically lighter sources indicated by the isotope depletion ( Table 5). The dispersion of the samples may reflect heterogeneity in recharge sources, mixing with deeper groundwater or longer water to rock contact time ^90, 104^, Figure 8.

The chemical composition of groundwater samples collected during the second campaign reflects a range of geochemical processes influenced by water–rock interaction and rock differences ( Table 6). The Tropeouhos and Ammohori_1 sample chemical results indicate active water and rock interaction involving iron-bearing minerals that undergo redox processes with Fe ^3+^ (insoluble ferric) → Fe ^2+^ (soluble ferrous) in anoxic environments ^91, 106^. High concentration of Fe can be associated with dissolution of iron bearing minerals or the serpentinization process ^71^. Both processes favour natural hydrogen production ^22, 33, 107^. The same applies to a lesser extent for the Mesohori and Katakali samples.

High CO _2_ concentrations in Itea (60.7%), Mesokampos (~60%), and Neos Kafkasos (~40%), including Ammohori_1 and _2 (~7–8% CO _2_) are related to the Florina basin, where natural CO _2_ of volcanic origin has accumulated in the Miocene fluvial sandstones ^69, 95^. The accumulations migrating via faults have been detected from depths of 296–338 m and 366–372 m below the surface and are well reported ^69^. They also provide CO _2_ analogues for CO _2_ storage, which is also related to the Pilot Strategy Project ^61^.

## Origins of gases: biotic, abiotic and radiogenic signatures

To evaluate whether the detected gases have biotic or abiotic origins, as addressed by the second objective of the scientific hypothesis, pH conditions, isotopic signatures, and dissolved element concentrations were examined. These parameters provide diagnostic fingerprints that allow one to distinguish between microbial gas production, water–rock reactions that generate abiotic gases, and crustal or mantle sources that contribute radiogenic signatures.

The pH values of all samples ranged from 5.4 to 8.48 ( Table 2– Table 5). Most samples were slightly acidic (around pH 5.4), suggesting interaction with silicate-rich lithologies, likely weathered ophiolites, or the influence of organic-rich soils. In contrast, the Katakali sample exhibited a distinctly alkaline pH (8.48), potentially indicating interaction with carbonate rocks (e.g. Triassic–Jurassic limestones) or with serpentinized ultramafic rocks undergoing low-temperature alteration, which commonly release hydroxide ions and elevate pH ^7, 22, 33^.

Hydrogen concentrations provide further insight into abiotic gas-generating processes. In the third sampling campaign, detectable H₂ was identified at Katakali (4.4 ppm), Kivotos (1.7 ppm), and Neos Kafkasos (2 ppm). Although H _2_ values are close to instrument detection limits, their repeated occurrence at the same sites, except for Katakali, suggests genuine geological signals rather than analytical artefacts. Hydrogen is an indicator of water–rock reactions, typically associated with a) serpentinization of ultramafic rocks (e.g., ophiolites), b) radiolysis of water in fractured rocks under the influence of natural radioactivity and c) anaerobic corrosion of Fe-bearing minerals, particularly under reducing conditions. These processes are common in tectonically active zones, where fluid pathways are enhanced ^7, 13, 14, 16, 22, 33, 40^ and discussed further in the next section.

The Katakali sample's combination of high pH, elevated Li and B ( Table 6), and relatively low transition metal concentrations points toward interaction with serpentinized ultramafic rocks. These lithologies commonly generate hyperalkaline fluids through serpentinization reactions and are known potential sources of natural hydrogen ^108, 109^. However, methane pattern at Katakali diverges from this abiotic H₂ pattern and suggests a different origin.

Methane patterns show a contrasting behaviour. In the third sampling round, Katakali contained 3,704 ppm CH₄, whereas the fourth sampling campaign revealed high concentrations (>300,000 ppm) in both duplicates, along with a slightly alkaline pH (~8). These values suggest methane generation under anoxic ^73^, closed-system conditions, consistent with deep microbial environments ^110^ rather than shallow aquifers, which are constantly replenished. This interpretation is supported by the isotopic composition of δD _H₂O_ and δ ^18^O _H₂O_ from the fourth round, which indicates mixing with deeper, older groundwater. The absence of helium and hydrogen, combined with δ ^13^C _CH₄_ values around –70‰ and δD _CH₄_ near –230‰, strongly supports biogenic methane via microbial CO _2_ reduction ^43, 111^ ( Figure 9). A secondary, less likely mechanism may involve abiotic H₂ generated by serpentinization that is subsequently oxidised while CO₂ is reduced to methane under oxic conditions by cyanobacteria ^73^ during migration through mixed groundwater zones. Further investigation may be needed to properly distinguish between bacterial imprint and diffusive fractionation ^93^. For reference, Daskalopoulou *et al*. 2018 ^18^, previously detected 4 ppm He and 11 ppm H _2_ at Katakali, suggesting spatial or temporal variability in gas inputs.

In contrast to Katakali’s microbial methane signature, helium distributions reveal a strong radiogenic component at other sites. Tropeouhos (1403 ppm He) and Kivotos (29 ppm He) samples show significant helium enrichment, which is not produced in shallow processes. Such high He levels suggest input from a radiogenic source in crystalline basement rocks (U/Th decay) ^112^, mixing with mantle-derived fluids migrating through deep-seated fault systems ^112^. Even the low but measurable helium at Neos Kafkasos (6 ppm) aligns with this interpretation and reflects its structural proximity to Tropeouhos, indicating shared deep fluid pathways.

While gas compositions provide direct evidence of source processes, dissolved element concentrations complement these data by revealing the redox and lithological conditions that influence gas generation and migration.Samples from Ammohori 1 and Tropeouhos displayed high levels of Fe (up to 2807 μg/L), Mn (up to 444 μg/L), and Al (up to 1455 μg/L), indicating reducing conditions that mobilise these elements from iron and manganese oxides and aluminosilicates. Such elevated concentrations are related to reductive dissolution of Fe/Mn oxides under anoxic conditions ^71, 113^, contribution from ophiolitic or volcanoclastic host rocks ^108, 109^ and weathering of feldspars ^114^ and/or clay-rich layers typically found in flysch or schist rocks ^71, 115, 116^.

## Migration pathways and fluid circulation

The third objective of the scientific hypothesis presented in the introduction is to understand how gases migrate and accumulate in the study area. This is achieved by integrating trace-element geochemistry, stable isotopes, and gas compositions to infer the depth, direction, and mechanisms of subsurface fluid flow. The presence of Li, B, Br, noble gases and reducing conditions provide critical constraints on circulation depth, residence time, lithological interactions, and the degree of confinement of the system ^43, 109, 117^.

The Katakali and Tropeouhos samples from the third sampling campaign show elevated concentrations of Li, 131 and 406 μg/L, respectively, indicating deep water circulation interacting with igneous or silicate rocks ^43^. This interpretation is consistent with the isotopic compositions shown in Figure 8 for the third-round section, depicting evolved signatures characteristic of prolonged water–rock interaction. Such conditions also favour the preservation of natural hydrogen generated by serpentinization or radiolysis ^22^.

Tropeouhos, Katakali, and Mesokampos also show high values of B (up to 7160 μg/L), and Br (up to 363 μg/L). These elements are typically enriched in hydrothermal systems ^71^, marine sedimentary or evaporite-hosted aquifers ^118^ or fluids with long residence times and extensive water–rock contact ^109^. In Mesokampos, concurrent B–Br enrichment and high CO₂ (~60%) are consistent with a marine or connate water signature, suggesting mixing with residual basinal brines or deep-seated formation waters ^118, 119^. Slight enrichment in δD and δ ^18^O supports a history of evaporation and prolonged water–rock interaction rather than modern meteoric recharge. All the aforementioned information points toward deep fluid circulation and long water- rock interaction.

**Figure 10.**
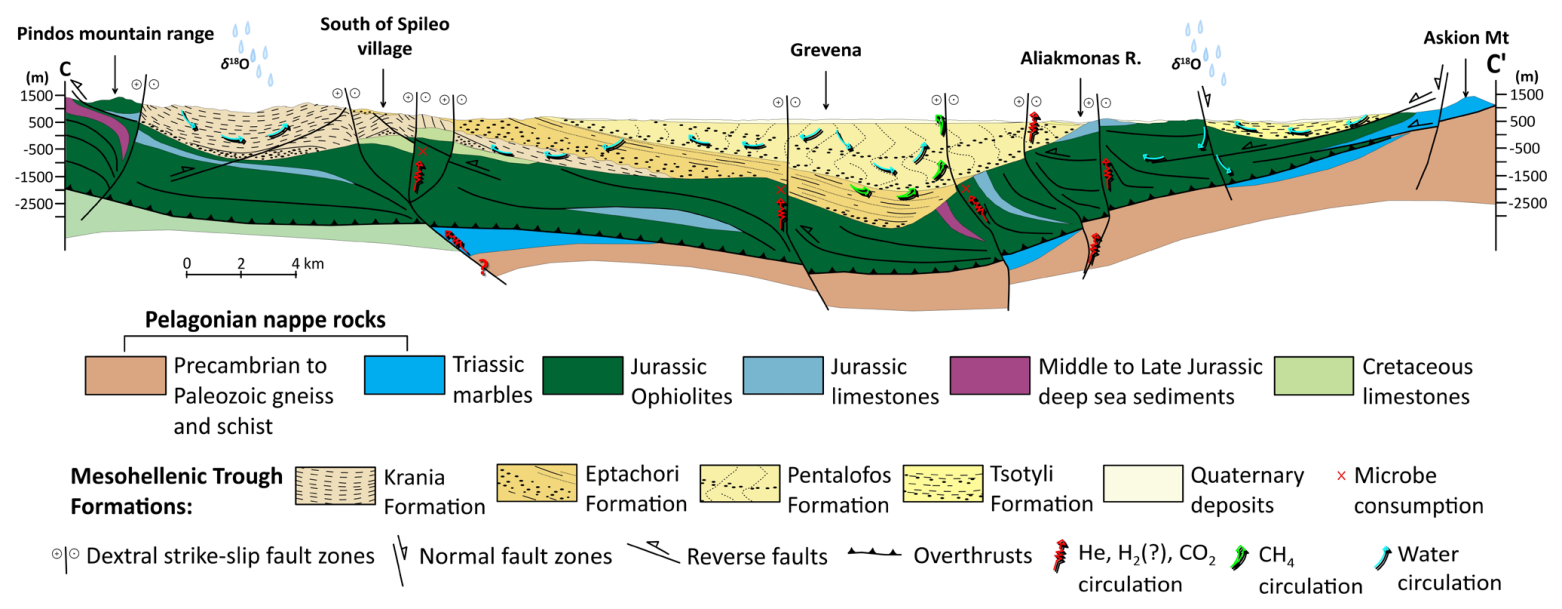
Geological cross section of Mesohellenic Basin with interpretative subsurface gas generation and migration mechanisms, licence: CC-BY 4.0.

At Katakali, moderately enriched δD _H₂O_ and δ ^18^O _H₂O_ values indicate some evaporation, but the dominant control appears to be long residence time and water–rock interaction with organic-rich strata (possibly lignites or deep carbonate strata). The interpretation from the data received from the sample reflects a closed-system ^120^ microbially dominated environment with limited gas migration, depicted in Figure 10.

Despite high CH₄ (>300,000 ppm), the absence of He and H₂ implies limited upward migration of mantle-derived or serpentinization-related gases ^43, 111, 121^. This contrasts with Daskalopoulou *et al*. (2018) ^18^, who reported minor amounts of He and H₂. Such variation is not contradictory and may reflect differences in sampling procedure, sample preservation, analytical uncertainty, or transient pore-water flushing ^122, 123^. However, both studies converge on identifying high methane concentrations that point towards a biotic origin. This study indicates a closed-system microbial gas generation and limited vertical migration for the Katakali site.

Kivotos presents a mixed gas system in which He (29–44 ppm) and H _2_ (1.7 ppm) co-occur with substantial methane (~43,000 ppm). Helium and hydrogen indicate abiotic sources, such as serpentinisation of ultramafic rocks or radiolysis in fractured basement lithologies ^111, 112^. CH _4_ may derive from either microbial or abiotic origin the low δ¹³C _CH₄_ values (–75.5‰ to -69.5‰,) remain firmly within the microbial range ^43, 124^, whereas δD _CH₄_ values (–107 to –92‰) are less depleted than typical biogenic methane ( Figure 9). These characteristics suggest transitional or mixed methane, possibly microbial CH₄ modified by thermogenic influence or by hydrogen exchange during migration. Slight δD _H₂O_ and δ ^18^O _H₂O_ enrichment supports an intermediate residence time and moderate water–rock interaction ( Figure 8, fourth round). Kivotos, therefore, may represent a hybrid gas system where microbial CH₄ coexists and possibly interacts with deeper, abiotic fluid inputs.

Tropeouhos is dominated by high helium values (up to 1403 ppm), low CH _4_ (81 ppm), with no detectable H _2_. This signature is characteristic of radiogenic He from U–Th decay in basement rocks or mantle-derived He transported along deep-seated fault structures ^19– 21^. The low CH _4_ and absence of H _2_ suggest minimal microbial or abiotic CH _4_ generation ^30, 43, 111, 121^. Tropeouhos possibly represents an active degassing zone, where He migrates vertically along tectonic conduits ^38^ rather than accumulating in a confined reservoir.

The Neos Kafkasos with low but detectable He (10 ppm) and H _2_ (2 ppm) combined with CO _2_ at ~50% suggest (a) interaction with deep carbonate reservoirs undergoing dissolution, (b) magmatic CO₂ inputs associated with known volcanic activity in the Florina basin ^95^ and c) mildly reducing conditions that promote CO₂ accumulation ^67, 69, 76– 78^. Although δ¹³C _CH₄_ was not obtained due to the low concentration of CH _4_, the available data on δ¹³C _TDC_ (–1.2‰) and δD _H₂O_ (–65‰) suggest thermogenic or mixed gas influence ^125^.

Based on the proximity of Mesokampos and Itea, both with elevated CO _2_% values, a regional CO _2_ dominated system is suggested, with deep-origin CO _2_ migrating upward along structural pathways. He and H _2_ values indicate connectivity to deeper sources.

## Trapping mechanisms and localized reservoir behaviour

The fourth objective of the scientific hypothesis is to evaluate how geological formations within the Mesohellenic Basin generate, host, and trap hydrogen and helium. In this study, the objective is approached by integrating mineralogical compositions, sedimentary facies, petrophysical properties, and redox behaviour to assess both (a) the potential for hydrogen formation through serpentinisation, radiolysis, or Fe-bearing mineral alteration, and He through U–Th decay and (b) the stratigraphic and structural conditions that permit gas accumulation and preservation.

The Mesohellenic Basin’s tectonic inheritance from Tethyan ophiolite obduction, its thick multilayered siliciclastic stratigraphy, and its extremely low-permeability greywacke and marl successions have the potential for gas generation, retention, and long-term preservation of geogenic gases. The ophiolitic basement ( Figure 2) provides the mineralogical combination for abiotic H _2_ and radiogenic He production; the basin sediments and caprocks provide confinement; and structural reactivation governs migration.

All samples contained detectable Sr and Ba, consistent with the dissolution of carbonates and feldspathic minerals across the Mesohellenic Trough. Sr indicates interaction with marls, limestones, plagioclase-rich sandstone units ^126^, and mixed litharenites. The third-round water samples from West Macedonia show a complex mixture of dissolved gases, reflecting interactions between geological formations, redox conditions, and possibly deep-seated fluid sources.

The mineralogical framework of the basin is dominated by the Eptachori, Pentalofos, and Tsotyli Formations, deposited in a high-energy environment with significant siliciclastic input. Their petrophysical and structural properties exert strong control on gas migration and confinement. Unhealed discontinuities within these formations can act locally as fluid migration pathways, whereas major faults provide a significant migration mechanism.

Eptachori Formation greywackes are fine-grained, strongly cemented, and composed of quartz, calcite, albite, muscovite and accessory titanite, biotite, actinolite and zircon ^127^. Their Fe–Mg silicates and U–Th–bearing minerals enable radiolytic H₂ and radiogenic He generation, while extremely low permeability (<0.01 mD) and low porosity (~7.4%) ^61^ favour long-term gas retention.

Pentalofos Formation sandstones and greywackes contain higher-energy, mixed lithic detritus, including chromite, chlorite, pyrite and actinolite, derived from the underlying ophiolitic and metamorphic massifs ^127^. With porosities of 4.9–10.8% but similarly low permeability (<0.01 mD) ^61^, these units offer only limited reservoir potential, although their mineralogy may support some localised serpentinisation-derived H _2_ and radiogenic He formation.

Tsotyli Formation marly sandstones and conglomerates exhibit moderate porosity (~6%), extremely low permeability, and strong mechanical strength, functioning as regional sealing units ^61^ that inhibit vertical migration.

The non-lignite interbeds of the Florina Basin (marl and sand) consist primarily of illite, mica, kaolinite, quartz, ferroan chlorite and albite, with negligible carbonates ^127, 128^. These fluvio-lacustrine sediments form a redox-active matrix capable of buffering Eh, promoting Fe–Mn mobilisation, and supporting microbial methanogenesis or hydrogen consumption. Their silicate-dominated composition enhances water–rock reactions and contributes Sr, Ba, Fe and Mn to groundwater, consistent with the hydrochemical signatures observed at Tropeouhos and Ammohori. Gemeni *et al.*, 2015 ^71^ demonstrate that Fe–Mn–Br enrichment in this region derives from interaction with deeper igneous and ophiolitic substrates rather than hydrothermal activity, reinforcing the role of the ophiolitic basement and potential its derived detritus in H _2_ and He generation.

As discussed in the previous sections, elevated values of Li (up to 406 μg/L), B (up to 7160 μg/L), and Br (up to 363 μg/L) in Katakali, Tropeouhos, and Mesokampos indicate deep fluid residence, prolonged circulation through silicate and evaporitic strata, and potential mixing with connate brines. Stable isotopes (δD _H₂O_, δ ^18^O _H₂O_) show displacement from modern meteoric signatures toward more evolved compositions, confirming long residence times, deeper infiltration and significant water–rock interaction ^19– 21, 30, 43, 67, 69, 76– 78, 111, 121^. The geochemical data support the interpretation that the area hosts mixed deep–shallow circulation systems, where ultramafic, carbonate, and organic-rich lithologies influence gas composition.

Petrophysical data from across the basin show very low permeability (<0.01 mD) in all formations, ensuring strong confinement; low porosity (4–10%), limiting storage capacity but enhancing residence times for helium; high clay-bound water fractions (87–97%), reducing effective pore space and increasing sealing efficiency, high mechanical strength, particularly in Eptachori and Tsotyli, supporting their behaviour as regional caprocks ^61^.

It is suggested that the Mesohellenic Basin behaves as a multilayered, strongly confined system. In this geological setting helium accumulates preferentially in low-permeability horizons near U–Th-bearing minerals (e.g., ophiolitoc basement, Eptachori, Pentalofos) and migrates along deep reactivated faults (Tropeouhos), producing high He anomalies. Hydrogen is generated via serpentinisation of ultramafic detritus (ophiolite-derived), radiolysis of U–Th minerals, or Fe-bearing mineral alteration. It is preserved only where reducing, low-permeability compartments exist (e.g., occasional signatures at Kivotos), but is absent where microbial or redox consumption dominates (Katakali). Methane is preserved in closed or semi-closed microbially dominated pockets (e.g., Katakali) and it is scarce in structurally open or fault-connected systems (Tropeouhos).

## Geological and exploration implications

The fifth objective of the scientific hypothesis is to assess resource potential and energy implications by estimating the natural hydrogen volume available for use. The geochemical investigation has identified in Kivotos and Neos Kafkasos detectable H _2_, He, evolved δD–δ¹⁸O signatures and B–Br enrichment. These data indicate that deep fluid circulation interacts with ultramafic substrates, confirming the existence of generative processes and active migration pathways. However, the absence of He and the limited presence of H _2_ at Katakali, despite serpentinisation-compatible geochemistry, highlight that if hydrogen is generated, it is susceptible to redox consumption in microbially dominated or organic-rich settings. Thus, resource-grade accumulations are unlikely in reducing compartments but may be preserved in more oxidised or structurally open domains where hydrogen escape is constrained by stratigraphy rather than biology.

From an energy perspective, the basin configuration is favourable for small- to medium-scale accumulations concentrated along fault-proximal compartments that overlie the ophiolitic basement Figure 10. These areas show the most substantial overlap between generative lithologies, evidence of deep fluid ascent (He anomalies), and trapping conditions capable of preserving geogenic gases across geological timescales. While the current data do not permit formal volumetric estimation, the combination of source potential, seal integrity, long residence times, and structural compartmentalisation suggests that high-resolution structural imaging and targeted hydrogeochemical surveys could delineate zones with recoverable natural hydrogen.

## Conclusion

The West Macedonia, Greece, presents the complex basement geology with felsic rocks ideal for radiolysis, ophiolites for serpentinisation and pervasive crustal faulting for degassing. The geochemical investigation supports the interpretation of multiple water sources or flow paths, including both shallow, oxygenated meteoric water and deeper, more evolved fluids enriched in light elements. These findings fulfil the first objective of the scientific hypothesis by demonstrating that the lithological and tectonic framework required for geogenic H _2_ and He production is present in the region. They also partially satisfy the second objective by showing that both biotic and abiotic processes may contribute to gas generation, even though direct evidence for sustained hydrogen production remains limited due to low measured concentrations and potential microbial consumption.

Overall, the results point toward a migration-dominated system in which gases undergo chemical modification along their ascent, with no confirmed large accumulations to date aside from the known natural CO _2_ reserves. This constrains the fifth objective of the scientific hypothesis, indicating that while a generative geological setting exists, present data do not yet support volumetric estimates of recoverable natural hydrogen.

In such cases, isotopes still point to dominantly biogenic CH _4_ even if the gas migration system involves ophiolitic or deep rocks. Specifically, biogenic methane dominates in confined organic-rich environments (Katakali), while He and H _2_ signals at Kivotos and Tropeouhos suggest deep crustal or mantle-derived fluids, potentially linked to serpentinization or radiogenic decay. Sites like Kivotos are most promising for mixed hydrogen generation, with some abiotic gas potential. Tropeouhos may serve more as a gas migration indicator, while Neos Kafkasos indicates natural CO _2_ degassing rather than accumulation.

Where He and H _2_ were detected (e.g., Kivotos), the system likely involves both deeper lithological interaction (ultramafics/fracture pathways) and microbial methane generation in overlying sediments ^117^ or mixed geological reservoirs ^73^. In such cases, isotopes still indicate biogenic CH _4_ even if the gas migration mechanism involves ophiolitic or deep rocks. On the same note H _2_ is difficult to find in the surface springs due to easy microbial consumption in the water ^129– 134^ and rapid oxidation along shallow circulation pathways. These limitations directly affect the evaluation of reservoirs and trapping behaviour which falls under the investigation of the third objective.

However, the present chemical analysis from Katakali samples reinforicing previous research from Daskalopoulou *et al.*, 2018 ^18^ that also reported helium and hydrogen occurrences. A pausible explanation may be that abiotic H _2_ could be generated at depth but microbially consumed before reaching the sampling point ^135^. Thus, biogenic isotopic carbon signature masks the hydrogen abiotic origin ^136– 139^. The presence of helium indicates deep crustal or mantle fluid origins ^73^. For instance, microbial communities relying on a geologic supply of H _2_ have been identified in Precambrian cratons where ancient waters trapped in deep fractures that undergo radiolysis ^140^. Given the natural carbon dioxide abundance in the area ^69, 74, 128^ it is possible that H _2_ is consumed in higher depths by microbes ( Equation 3) ^141^ and thus sustain rock supported ecosystems ^142^ to produce methane. In this way the abiotic hydrogen isotopic fingerprint may be suppressed.


$$\text{C}{\text{O}}_{2}+4{\text{H}}_{2}\to \text{C}{\text{H}}_{4}+2{\text{H}}_{2}\text{O}\;(\text{Hydrogenotrophic}\;\text{Methanogenesis})\;(Equation 3)$$

In such a case methane isotopes alone can be misleading about the hydrogen origin ^73^ and additional tracers such as helium, clumped isotopes ^143^, dissolved hydrogen monitoring and sulphur isotopes will need to be taken into account. For instance, δ¹³C _CH₄_ can be composition insensitive to mixing with microbial gases ^38^ while microbial SO _4_^2−^ reduction to H _2_S is a process that occurs in anaerobic environments ( Equation 4) in high depths including lacustrine sediments and groundwater ^144^ that can also alter the hydrogen’s abiotic origin.


$$\text{S}{\text{O}}_{4}^{2-}+4{\text{H}}_{2}\to {\text{H}}_{2}\text{S}+2{\text{H}}_{2}\text{O}\;(\text{Hydrogenotrophic}\;\text{Sulphate}\;\text{Reduction})\;(Equation 4)$$

Thus, sulphur isotopes must be investigated to detect an abiotic hydrogen source ^73^. Therefore to properly understand the origins of the hydrogen and methane in the area a combined geochemical–microbiological approach is needed. This directly aligns with the fourth, which is partially fulfilled by demonstrating the role of mixed biotic–abiotic processes but requires further evidence to fully resolve gas origins.

The afforementioned findings and discussion demonstrate the need for further investigation, particularly dedicated gas flux monitoring, mineralogical sampling, and structural mapping to delineate potential H _2_-prone zones and assess their economic relevance. This preliminary study sheds light on the complex but dynamically evolving systems that dominate the Mesohellenic and Florina basins. Whereas hydrogen and helium may exist in economically viable quantities in the area, the question remains to be answered. Still, the current fluid generation and flow mechanisms existing in the Mesohellenic and Florina basin can shed light and provide analogues for exploration and discovery of economic accumulations.

It is well known that hydrogen seepage on the surface is not constant and may change the point of escape over time or flow rate ^145^ while sampling in water comes with its drawbacks ^129– 134^. Thus, future work should focus on data collection from Katakali, Kivotos, Tropeouhos and Neos Kafkasos over a long period of time rather than being spontaneous. Since the data collection has been conducted on water samples rather than in soil, it is suggested that the aforementioned sites be investigated with a handheld GA5000 or similar over a period of six months or a year on a weekly basis. The day of the visit, the operator should collect at least 6 hourly readings of hydrogen. Should the operator register hydrogen readings, water samples with vials should be taken for the following analysis:

1. ICP for a chemical suite analysis
2. He
3. H _2_
4. CH _4_
5. CO _2_
6. Helium isotopes ( ^3^He, ^4^He) ^146^
7. Deuterium of hydrogen (δ ^2^H _H₂_)
8. Deuterium of methane (δ ^2^H _CH₄_)
9. Deuterium of water (δ ^2^H _H₂O_)
10. Oxygen-18 of carbon dioxide (δ ^18^O _CO₂_)
11. Oxygen-18 of water (δ ^18^O _H₂O_
12. Carbon-13 of methane (δ¹³C _CH₄_)
13. Carbon-13 of carbon dioxide (δ ^13^O _CO₂_)
14. Carbon-13 of DIC (δ ^13^C _DIC_)
15. Carbon-14 of DIC ( ^14^C _DIC_)
16. Sulphur isotopes (δ ^34^S)
17. Clumped isotopes (Δ¹³CH _3_D, Δ ^12^CH _2_D _2_) ^73, 146^

Microbial sequencing to evaluate consumption pathways and metabolic signatures.

To fully understand the local system, it is necessary to sample precipitation and surface water, in addition to groundwater ^90^. If any of the sites provide satisfactory results, then, provided that the appropriate permits are granted, permanent equipment should be installed in the springs to continuously monitor for hydrogen and helium. Further to this, a full exploration program with geological mapping and targeted geological soil ^19– 22, 24– 28, 30– 33, 35, 37, 40, 41, 43, 44, 46, 48– 71, 74– 84, 87– 95, 100– 109, 111– 116, 118– 121, 124– 134, 136, 147– 153^ and water sampling with intrusive investigation should then take place as a prerequisite for addressing the fifth objective, which remains only partially fulfilled due to insufficient data to estimate natural hydrogen volumes.

## Ethics and consent

Ethical approval and consent were not required.

## Data Availability

Zenodo: https://doi.org/10.5281/zenodo.16914636 ^89^ This project contains the following underlying data: Water_samples_1stround_100ml Water_samples_2ndround_250ml Water_samples_3rdround_100ml Water_samples_4thround_100ml Field_Dataentry_form_1st round Field_Dataentry_form_2nd round Field_Dataentry_form_3rd round Field_Dataentry_form_4th round Assigned codes_1st-3rd rounds Assigned codes_4th round Geochemical analysis_1st round Geochemical analysis_2nd round Geochemical analysis_3rd round Geochemical analysis_4th round Stratigraphy Data are available under the terms of the Creative Commons Attribution 4.0 International license (CC-BY 4.0).
