# Supplementary material for: Investigative research for occurrences of hydrogen, helium, methane and carbon dioxide in West Macedonia: linking geological reservoirs to subsurface gas generation and migration
Source: Open Res Eur. 2026 Apr 13;5:274. Originally published 2025 Sep 5. [Version 3] doi: 10.12688/openreseurope.21180.3 (PMC12963835; doi:10.12688/openreseurope.21180.3)
Supplement: Peer review history

# Reviewer report: Reviewer response for version 3

**DOI:** 10.21956/openreseurope.25270.r72208

**Published:** 2026-04-16

**Authors:** John Begg

**Affiliations:** 1 National Observatory of Athens, Institute of Geodynamics, Athens, Greece

I am satisfied that the authors have addressed most of my comments adequately. Paper approved.

Is the study design appropriate and does the work have academic merit?

Yes

Is the work clearly and accurately presented and does it cite the current literature?

Partly

If applicable, is the statistical analysis and its interpretation appropriate?

Partly

Are all the source data underlying the results available to ensure full reproducibility?

Yes

Are the conclusions drawn adequately supported by the results?

Partly

Are sufficient details of methods and analysis provided to allow replication by others?

Yes

Reviewer Expertise:

NA

I confirm that I have read this submission and believe that I have an appropriate level of expertise to confirm that it is of an acceptable scientific standard.

# Reviewer report: Reviewer response for version 2

**DOI:** 10.21956/openreseurope.23969.r69397

**Published:** 2026-03-10

**Authors:** Rebecca Tyne

**Affiliations:** 1 The University of Manchester, Manchester, England, UK

This paper uses dissolved gas and gas measurements (primarily H2, He and CO2) in springs across West Macedonia and combine this with the lithological and structural setting to identify different gas sources and migration within the region. The authors have generated an impressive dataset of gas chemistry, ions and methane isotopes across different springs and also through time, which will benefit the whole community. Despite this, I think that the paper could benefit with some further work on its structure and clarity in order to make more impactful conclusions, which I think are currently a bit lost (See below for more details).

Major comments

 You have structured your discussion to assess each spring individually- while good I find it is lacking a bit of cohesion on the bigger picture. Which makes it hard to see overall trends (in space and time in the region) and draw conclusions especially relating to linking to the geology. It would be great to structure the discussion in a way that the bigger picture and trends are also consideration.

 Supra regional and regional context could be shortened in geological context and moved to sup- currently distracts a bit form message and the area context more relevant.

Moderate comments

- In the abstract, the conclusion section is incredibly vague. For example, “the preliminary investigation indicates the existence of multiple gas generation and migration mechanisms”, such as what?
- Water isotopes, suggest you see seasonable variability; however, ions and noble gases suggest that there has been significant water rock reaction and a longer residence time. I’m a bit confused by this as it seems contraindicatory.

Minor comments

  

- Check for consistent super- and sub- scripting, as well as the use of d not just d.
- Easily replicate methods, but Figure 7 of sampling containers not necessary.

Introduction:

- Page 4 para 1, line 13 -  West Macedonia should come before Greece
- Page 4, para 3, bullet 1 – I assume you mean in contact with mantle fluids within the crust? I think it's probably better just to say reducing agents, especially because the references used for the mantle don’t actually back up the statement you have made (Bendall et al., talks about mantle degassing as a source, Lefuerve is focused on mantle derived rocks being serpentinised and Mao is focused on the mantle core boundary- and any H2 is the curst from this would be degassing mantle (bullet 8).
- Page 4, paragraph 6 (last paragraph) – I find this paragraph difficult to follow.
- Page 5, paragraph 2 - Hydrogen formation doesn’t always have to happen at high depths, for example in Oman there is significant H2 formation at 100-1250m depth (Templeton et al., 2024 *refer to reference 3)*
- Page 5, paragraph 2 - why is abiotic methane, brucite and carbonate indicators for the presence of natural hydrogen? (For example, one could argue that the methane could actually be an indicator that the any natural H2 that was present has been converted to CH4).
- Page 5, paragraph 2 - I don’t follow the last sentence of this paragraph.
- Page 5 paragraph 6- Could you be more explicit here? For example, “In this study, the ophiolite has been thrusted on top of carbonates, which act as a source of CO2 to the system, which can subsequently reaction with any H2 produced (e.g. equation 2). Although this reaction is slow in its abiotic form, it can still be significant over geological time. **I would also like to note this reaction can be significantly quicker if it is biotically driven, see Gray et al., 2009 (refer to reference 1), Tyne et al., 2021(refer to reference 2)

*Supra regional geological context*

- Page 5, para 7 line 19 – I think you mean eastern not easter?
- Page 6, para 2 – you seem to have slipped into results here talking about the origin of methane from its isotopic composition?
- Page 6, para 3 – dissolved CO2 can still migrate when dissolved so it is technically not immobilised. Not sure what you mean in the next sentence of this paragraph.  

Results

- Table 7- more significant figures are needed to show results the He and H2 results.
- Table 7 – what are the errors on all these data?

Discussion

- Table 8 – can you go to more sig fig again so you can see actual values not just 0? Also make sig figs consistent between the tables.
- From the tables there quite a significant chang in the gas composition between campaign 3 and 4, it would be good if this could be further explained in the text.

Discussion origin of gases:

- Page 21, para 3 – have you thought about looking at the He/H2 ratio to determine if it is form radiolysis?
- Page 21, para 4 - why does microbial methane mean there is no abiotic H2?
- Page 21, para 6 -  what other sites?

Discussion, migration pathways:

- It would be worth redrafting this section to make your point clearer as I find it a bit hard to follow.
- Page 22, para 3 – why does the absence of He and H2 imply limited upward migration of mantle gases? The amount of He is the mantle is low compared to that generate in the crust and already in the water, so while mantle He could substantially change the 3He/4He ratio (which does not seem to have been measured) in the sample, the actual concentration of He is not likely to substantially change. H2 may also be migration from below and then being converted to methane or methane could be forming at depth then migrating up? In addition, He could have been lost due to the type of sampling container used

Is the study design appropriate and does the work have academic merit?

Yes

Is the work clearly and accurately presented and does it cite the current literature?

Partly

If applicable, is the statistical analysis and its interpretation appropriate?

I cannot comment. A qualified statistician is required.

Are all the source data underlying the results available to ensure full reproducibility?

Yes

Are the conclusions drawn adequately supported by the results?

Yes

Are sufficient details of methods and analysis provided to allow replication by others?

Yes

Reviewer Expertise:

Isotope tracing of CO2, He and H2

I confirm that I have read this submission and believe that I have an appropriate level of expertise to confirm that it is of an acceptable scientific standard, however I have significant reservations, as outlined above.

## References

[1] : Biogenic methane production in formation waters from a large gas field in the North Sea. *Extremophiles* .2009;13(3) : 10.1007/s00792-009-0237-3 511-519 10.1007/s00792-009-0237-3 19305943

[2] : Rapid microbial methanogenesis during CO2 storage in hydrocarbon reservoirs. *Nature* .2021;600(7890) : 10.1038/s41586-021-04153-3 670-674 10.1038/s41586-021-04153-3 PMC869537334937895

[3] : Low-temperature hydrogen production and consumption in partially-hydrated peridotites in Oman: implications for stimulated geological hydrogen production. *Frontiers in Geochemistry* .2024;2: 10.3389/fgeoc.2024.1366268 10.3389/fgeoc.2024.1366268

# Reviewer report: Reviewer response for version 2

**DOI:** 10.21956/openreseurope.23969.r69217

**Published:** 2026-03-05

**Authors:** Biying Chen

**Affiliations:** 1 University of Edinburgh, Grant Institute, Edinburgh, Scotland, UK

This study provides a comprehensive geochemical analysis of water and dissolved gases in West Macedonia, aiming to identify potential geological reservoirs, gas sources, and migration routes, with a specific focus on helium and hydrogen. While it is evident that the authors have put significant effort into revising the manuscript and improving its structural flow, several fundamental concerns remain regarding the core contribution of this study.

 The primary issue relates to the focus on natural hydrogen exploration. Given that only trace levels of hydrogen were detected in the study areas, the rationale for selecting this region as a target for hydrogen resource evaluation remains unclear. The authors suggest in their response that the study focuses on processes rather than volumes in the revised version. However, the hydrogen generation mechanisms discussed do not appear to move significantly beyond established general knowledge. Furthermore, the proposed generation mechanisms appear to be inferred indirectly from trace elements and CH4, CO2, and He data. Consequently, the constraints on hydrogen generation remain poorly defined. The authors should more clearly articulate the novel contribution of this work and how it advances the existing literature.

 Minor comments

 There appears to be a discrepancy in the interpretation of the water sources for the third round of sampling. The authors first attribute the signature to rainfall and evaporation. However, the discussion of trace element content later shifts the explanation to water-rock interaction.

 The authors suggest that high Fe concentrations in the Tropeouhos and Ammohori_1 samples may be linked to mineral dissolution or serpentinisation, which are processes that theoretically facilitate H2 production. However, no measurable H2 was detected in these specific samples.

 The manuscript attributes the high CO2 content in the samples related to volcanic CO2 in the Florina Basin. Please provide further evidence or geochemical justification to support this link.

 The term "TDC" and "DIC" are not defined.

 The authors conclude that the elevated helium levels originate from a mixture of radiogenic and mantle sources. However, no helium isotope data has been provided. In the absence of this data, it is difficult to definitively determine the origin.

Is the study design appropriate and does the work have academic merit?

Partly

Is the work clearly and accurately presented and does it cite the current literature?

Yes

If applicable, is the statistical analysis and its interpretation appropriate?

Partly

Are all the source data underlying the results available to ensure full reproducibility?

Partly

Are the conclusions drawn adequately supported by the results?

Partly

Are sufficient details of methods and analysis provided to allow replication by others?

Yes

Reviewer Expertise:

natural gas geochemistry

I confirm that I have read this submission and believe that I have an appropriate level of expertise to confirm that it is of an acceptable scientific standard, however I have significant reservations, as outlined above.

# Reviewer report: Reviewer response for version 2

**DOI:** 10.21956/openreseurope.23969.r69398

**Published:** 2026-03-02

**Authors:** John Begg

**Affiliations:** 1 National Observatory of Athens, Institute of Geodynamics, Athens, Greece

Review of the manuscript “Investigative research for occurrences of hydrogen, helium, methane and carbon dioxide in West Macedonia: linking geological reservoirs to subsurface gas generation and migration” by Tyrologou, P.; Koukouzas, N.; Couto, N.; Stergiou, C.L.; Carneiro, J. in Open Research Europe.

 This manuscript presents a valuable contribution to initial investigations into the prospectivity of natural hydrogen (and other gaseous species) in Western Macedonia, northern Greece. The paper incorporates discussion on the geology of the region with respect to identifying potential sources of natural hydrogen generation, fluid migration pathways, reservoir potential and possible biotic modification of gas species. Thorough geochemical analysis of gas and water samples from ten localities represents the scientific core of the paper with enhanced value provided by repeated sampling to assess seasonal/temporal variation. This reviewer commends the authors for their use of a multi-disciplinary toolkit to investigate gas and fluid origins and storage potential.

 Overall, this manuscript provides a significant contribution to the geochemical understanding of the occurrence of natural hydrogen, helium, methane and carbon dioxide in West Macedonia and is worthy of indexing subject to the (minor) revisions proposed below.

1. Abstract and PLS: I believe that both the Abstract and the Plain Language Summary can be improved by appropriately representing the scientific contribution of the paper. Currently, they both incorporate substantial sections that amount to political justifications for the work undertaken, an unnecessary distraction. I would prefer to see summaries of the scientific approach, data presented and conclusions. Further, I am not familiar with the style of presenting headings within an Abstract. Is this something that the Open Research Europe manuscripts accept?

2. Organization and length of introductory sections: The five stated objectives in the Introduction may have been ambitious, resulting in a lengthy manuscript. For example, the geological description of the region and area of interest occupies five and a half pages (over three sections). I believe that more focused summaries of potential source rocks, storage and cap rocks and their structure would help prepare readers adequately for what follows. Especially the last of the three introductory geological sections, under the heading “Local geology setting of promising sites for natural hydrogen”, adds little to the work at this stage, as the sampling and chemistry has not yet been discussed. Instead, this section should be relocated towards the end of the paper (Discussion?), once the authors have demonstrated prospectivity. Further, Figure 4 is rather empty and would benefit from some geological context (including faults; see comments below). 

3. Active faulting: While the section on geology is disproportionately long, there is no discussion on the active faulting and fold structures that may contribute to gas and fluid migration, and also to storage structures. In fact, a 2025 database, which was probably not available to the authors at the time of writing, provides this information and I encourage the author to consult it (Begg et al. 2025. Scientific Data.). The reason is that both Katakali and Kivotos sites lie close to NE-SW-orientated faults that have been active during the Quaternary (Begg et al. 2025). Similarly, a suite of active faults oriented NE-SW and a more limited set of NW-SE-oriented active faults are present in the Florina area (see Begg et al. 2025). Interestingly, it is unrealistic in my opinion that the authors of this manuscript favour strike-slip faulting in their figures 2 & 10, as neither geomorphic indicators (Begg et al. 2025) nor GNSS analysis (Chousianitis et al. 2024) or earthquake moment tensors (Konstantinou et al. 2017) support such mechanisms. Instead, the dominant faulting style in western Macedonia is normal faulting, which may result in the opening of fluid/gas migration pathways along active faults within the MHB.

4. Standardization of Tables: The geochemical data presented in Tables 2-8 are a primary output of this work and I congratulate the authors on this contribution. However, I was a little confused by the lack of continuity between the tables. In Table 1 the first column is spring names while the following columns locate them. In Tables 2-5, the first column is a number, but the numbers and names and the sequence of the springs sampled varies between these tables. This creates some confusion for the reader (and future users of the data). Then, the following tables revert to the spring names in the first column, but also incorporate the additional complexity of adding a second samples for some sites. Without carefully reading the text, readers could be forgiven for believing that these are different springs. Further complicating the data is that it appears that two Ammohori springs are sampled, Ammohori 1 and Ammohori 2. If springs are given consistent numbers and names, it will be intuitively clear to readers that the suffixes -1 and -2 refer to the sample numbers. Before the manuscript is accepted for indexing, the authors should make some effort to standardize their tables and nomenclature.

5. Results: I found this section to be rather disjointed. The first sentence states that isotope data will be discussed first and there is reference to Figure 8, but no presentation of the results. The results of ICP analyses that follows refers to Table 6; the text states that ICP analyses here are of water from the second round of sampling, but this is not stated in the caption. The presentation of water chemistry doesn’t appear to have much coherent description and there are embedded elements of interpretation within this discussion. In the Discussion and results interpretation section, there are some important observations that warrant inclusion within the Abstract, including that detected variations in water and gas chemistry probably reflect enrichment of resident groundwater by precipitation.

 6. Migration pathways and faults: the authors discussion regarding migration pathways and fluid circulation is useful but founded on geochemical analysis without reference to disruption of circulation related to active faulting. While this is understandable in the context of the data they have assembled, it leaves something of a question mark regarding the impact of active faulting on the system. I strongly recommend the authors discuss migration pathways also with respect to the active faulting in the region (Begg et al. 2025). Changes in gas and water chemistry at sample sites are interpreted as indicative of variability attributable to precipitation. However, some discussion of whether this is entirely attributable to shallow mixing of groundwater with rainwater influx, or whether the meteoric water infiltrates deeper parts of the basin, would benefit the manuscript.

Is the study design appropriate and does the work have academic merit?

Yes

Is the work clearly and accurately presented and does it cite the current literature?

Partly

If applicable, is the statistical analysis and its interpretation appropriate?

Partly

Are all the source data underlying the results available to ensure full reproducibility?

Yes

Are the conclusions drawn adequately supported by the results?

Partly

Are sufficient details of methods and analysis provided to allow replication by others?

Yes

Reviewer Expertise:

geological mapping, tectonics, active faulting, hydrogeology, natural hydrogen

I confirm that I have read this submission and believe that I have an appropriate level of expertise to confirm that it is of an acceptable scientific standard, however I have significant reservations, as outlined above.

## References

[1] : AFG - Active Faults Greece: a comprehensive geomorphology-based 1:25,000 fault database. *Scientific Data* .2025;12(1) : 10.1038/s41597-025-06283-z 10.1038/s41597-025-06283-z 41271816 PMC12639124

[2] : The Upper Crustal Deformation Field of Greece Inferred From GPS Data and Its Correlation With Earthquake Occurrence. *Journal of Geophysical Research: Solid Earth* .2024;129(4) : 10.1029/2023JB028004 10.1029/2023JB028004

[3] : Present‐day crustal stress field in Greece inferred from regional‐scale damped inversion of earthquake focal mechanisms. *Journal of Geophysical Research: Solid Earth* .2017;122(1) : 10.1002/2016JB013272 506-523 10.1002/2016JB013272

# Response

**Published:** 2026-03-03

**Authors:** Pavlos Tyrologou

**Affiliations:** Geo-RΕsources, EΝergy and environmental management, CERTH, Athens, Greece

*Response to reviewers* *Review of the manuscript “Investigative research for occurrences of hydrogen, helium, methane and carbon dioxide in West Macedonia: linking geological reservoirs to subsurface gas generation and migration” by Tyrologou, P.; Koukouzas, N.; Couto, N.; Stergiou, C.L.; Carneiro, J. in Open Research Europe.*

**This manuscript presents a valuable contribution to initial investigations into the prospectivity of natural hydrogen (and other gaseous species) in Western Macedonia, northern Greece. The paper incorporates discussion on the geology of the region with respect to identifying potential sources of natural hydrogen generation, fluid migration pathways, reservoir potential and possible biotic modification of gas species. Thorough geochemical analysis of gas and water samples from ten localities represents the scientific core of the paper with enhanced value provided by repeated sampling to assess seasonal/temporal variation. This reviewer commends the authors for their use of a multi-disciplinary toolkit to investigate gas and fluid origins and storage potential.**

**Overall, this manuscript provides a significant contribution to the geochemical understanding of the occurrence of natural hydrogen, helium, methane and carbon dioxide in West Macedonia and is worthy of indexing subject to the (minor) revisions proposed below.** **Response:** We thank the reviewer for the time invested to support our scientific research **1. Abstract and PLS: I believe that both the Abstract and the Plain Language Summary can be improved by appropriately representing the scientific contribution of the paper. Currently, they both incorporate substantial sections that amount to political justifications for the work undertaken, an unnecessary distraction. I would prefer to see summaries of the scientific approach, data presented and conclusions. Further, I am not familiar with the style of presenting headings within an Abstract. Is this something that the Open Research Europe manuscripts accept?** **Response:** We thank the reviewer for the support, dedicated time and this thoughtful comment. We fully agree that the primary function of both the abstract and the plain language summary is to clearly present the scientific approach, data, and conclusions of the study. At the same time, we considered it important to situate the work within its broader scientific and societal relevance. Our intention was not to provide political justification, but to clarify the scientific timeliness and contextual significance of the research question. We have revised the abstract to ensure that the description of methodology, analytical results, and principal findings are more prominent. Regarding the structured headings and the plain language summary format, these follow the Open Research Europe author guidelines and are consistent with the journal’s editorial requirements. **2. Organization and length of introductory sections: The five stated objectives in the Introduction may have been ambitious, resulting in a lengthy manuscript. For example, the geological description of the region and area of interest occupies five and a half pages (over three sections). I believe that more focused summaries of potential source rocks, storage and cap rocks and their structure would help prepare readers adequately for what follows. Especially the last of the three introductory geological sections, under the heading “Local geology setting of promising sites for natural hydrogen”, adds little to the work at this stage, as the sampling and chemistry has not yet been discussed. Instead, this section should be relocated towards the end of the paper (Discussion?), once the authors have demonstrated prospectivity. Further, Figure 4 is rather empty and would benefit from some geological context (including faults; see comments below).**

**Response:** Thank you for your valuable suggestions. The structure of the manuscript was adjusted in response to earlier peer review comments and reflects the way to establish the geological framework prior to presenting the geochemical results. We, thus, consider the stratigraphic, structural, and lithological context essential for understanding the sampling strategy, gas occurrence, and the interpretation of potential migration and trapping mechanisms. As such, the section “ *Local geology setting of promising sites for natural hydrogen*” is positioned within the introductory framework to provide the necessary geological basis for the subsequent analytical results. Figure 4 has been enhanced to include additional geological context and structural features, including faults, as suggested.

**3. Active faulting: While the section on geology is disproportionately long, there is no discussion on the active faulting and fold structures that may contribute to gas and fluid migration, and also to storage structures. In fact, a 2025 database, which was probably not available to the authors at the time of writing, provides this information and I encourage the author to consult it (Begg et al. 2025. Scientific Data.). The reason is that both Katakali and Kivotos sites lie close to NE-SW-orientated faults that have been active during the Quaternary (Begg et al. 2025). Similarly, a suite of active faults oriented NE-SW and a more limited set of NW-SE-oriented active faults are present in the Florina area (see Begg et al. 2025). Interestingly, it is unrealistic in my opinion that the authors of this manuscript favour strike-slip faulting in their figures 2 & 10, as neither geomorphic indicators (Begg et al. 2025) nor GNSS analysis (Chousianitis et al. 2024) or earthquake moment tensors (Konstantinou et al. 2017) support such mechanisms. Instead, the dominant faulting style in western Macedonia is normal faulting, which may result in the opening of fluid/gas migration pathways along active faults within the MHB.** **Response: ** We thank the reviewer for this insightful and constructive comment regarding the present-day tectonic regime of western Macedonia and its implications for fluid and gas migration. We agree that the dominant active faulting style in the region is normal faulting and that this should be clearly reflected in our interpretation. In response, we have revised the geological section to incorporate recent publications on active faulting and crustal deformation, (Begg et al., 2025), regional stress inversion results (Konstantinou et al., 2017), and GPS-derived strain rate analyses (Chousianitis et al., 2024). A new paragraph has been added at the end of the geological chapter explicitly stating that active and probably active faults in western Macedonia and the Florina Basin are dominantly normal, with NE–SW-oriented structures controlling Quaternary sedimentation and deformation. Importantly, we clarify in the Discussion that the fault geometries illustrated in Figures 2 and 10 do not aim to represent exclusively the present-day active tectonic regime, but primarily the inherited structural architecture formed during the Miocene opening and evolution of the Mesohellenic Basin. We now explicitly state that some of these pre-existing basin-bounding and intra-basin faults (as documented by publications studying the geometery of the MSB) may undergo selective reactivation under the current extensional stress field, predominantly in normal-slip mode, depending on their orientation relative to the present-day stress axes. This distinction separates the inherited tectonic framework from the Quaternary deformation regime and avoids implying an active regional strike-slip system. In addition, Figures 2 and 10 have been slightly modified and their captions revised to clarify that the depicted structures represent the inherited basin architecture and not necessarily the current kinematic regime. Together, these revisions align the manuscript with the geomorphic, seismological, and geodetic evidence cited by the reviewer, while preserving the structural controls relevant to subsurface gas generation and migration.  

**4. Standardization of Tables: The geochemical data presented in Tables 2-8 are a primary output of this work and I congratulate the authors on this contribution. However, I was a little confused by the lack of continuity between the tables. In Table 1 the first column is spring names while the following columns locate them. In Tables 2-5, the first column is a number, but the numbers and names and the sequence of the springs sampled varies between these tables. This creates some confusion for the reader (and future users of the data). Then, the following tables revert to the spring names in the first column, but also incorporate the additional complexity of adding a second samples for some sites. Without carefully reading the text, readers could be forgiven for believing that these are different springs. Further complicating the data is that it appears that two Ammohori springs are sampled, Ammohori 1 and Ammohori 2. If springs are given consistent numbers and names, it will be intuitively clear to readers that the suffixes -1 and -2 refer to the sample numbers. Before the manuscript is accepted for indexing, the authors should make some effort to standardize their tables and nomenclature.**

**Response: ** We thank the reviewer for this constructive comment and for recognising the importance of clarity and consistency in the presentation of the geochemical dataset. In response, we have thoroughly revised Tables 1–8 to standardize nomenclature and structure throughout the manuscript. All spring names are now presented uniformly in lowercase and bold font across all tables to ensure visual consistency and facilitate cross-referencing. In addition, we have assigned fixed identification codes (S1–S10) to the ten distinct springs, and these codes are used consistently across Tables 1–5. In Table 1, the listings of the springs were revised to be the same as in Tables 1 to 5. In Tables 6 and 7, the same S1–S10 labels have been added alongside the spring names to maintain continuity between datasets and sampling campaigns. To further eliminate ambiguity between distinct springs (e.g., Ammohori 1 and Ammohori 2) and duplicate samples collected from the same location, we have clarified in the captions of Tables 5 and 8 that “Samples marked with suffix (-2) represent duplicates”. We believe these revisions resolve the continuity issues identified by the reviewer and significantly improve the clarity, traceability, and usability of the dataset for readers and future users.

**5. Results: I found this section to be rather disjointed. The first sentence states that isotope data will be discussed first and there is reference to Figure 8, but no presentation of the results.** **Response: **We thank the reviewer for the comment. A paragraph has been added for text coherence. **The results of ICP analyses that follows refers to Table 6; the text states that ICP analyses here are of water from the second round of sampling, but this is not stated in the caption.** **Response:** Corrected **The presentation of water chemistry doesn’t appear to have much coherent description and there are embedded elements of interpretation within this discussion.** **Response:** Thank you for your comment. In the results section, we intentionally provide limited interpretative context alongside the presentation of water chemistry data to facilitate text coherence and continuity. Given the multidimensional nature of hydrogeochemical datasets, purely numerical reporting without brief explanatory linkage may obscure the relevance of observed spatial variability and elemental associations. Thus, the interpretative remarks included are restricted to immediate data description. Broader interpretation and synthesis are reserved for the Discussion section. **In the Discussion and results interpretation section, there are some important observations that warrant inclusion within the Abstract, including that detected variations in water and gas chemistry probably reflect enrichment of resident groundwater by precipitation.**

**Response:** Thank you for your comment. The abstract has being modified according to the reviewer’s suggestion.

**6. Migration pathways and faults: the authors discussion regarding migration pathways and fluid circulation is useful but founded on geochemical analysis without reference to disruption of circulation related to active faulting. While this is understandable in the context of the data they have assembled, it leaves something of a question mark regarding the impact of active faulting on the system. I strongly recommend the authors discuss migration pathways also with respect to the active faulting in the region (Begg et al. 2025). Changes in gas and water chemistry at sample sites are interpreted as indicative of variability attributable to precipitation. However, some discussion of whether this is entirely attributable to shallow mixing of groundwater with rainwater influx, or whether the meteoric water infiltrates deeper parts of the basin, would benefit the manuscript.** **Response:** We thank the reviewer for this valuable comment. We agree that the role of active faulting in controlling fluid circulation and gas migration pathways should be more explicitly addressed. In response, we have expanded the Discussion section (within *Hydrochemistry and isotopic evidence supporting gas occurrences*) to incorporate the influence of Quaternary normal faulting in western Macedonia, following Begg et al. (2025), Konstantinou et al. (2017), and Chousianitis et al. (2024). The revised text clarifies that active normal faults may enhance vertical permeability through fractured fault zones, thereby facilitating structurally controlled fluid circulation. We also refined our interpretation of hydrochemical variability to acknowledge that changes in gas and water chemistry may not be solely attributable to shallow mixing with meteoric water, but could also reflect deeper meteoric infiltration along active fault zones and inherited basin structures. This addition strengthens the linkage between geochemical observations and the regional active tectonic framework, without overextending beyond the constraints of the available data.

# Reviewer report: Reviewer response for version 2

**DOI:** 10.21956/openreseurope.23969.r66097

**Published:** 2025-12-30

**Authors:** Princewill M. Ikpeka

**Affiliations:** 1 Brunel University London, London, England, UK

No further comments.

Is the study design appropriate and does the work have academic merit?

Partly

Is the work clearly and accurately presented and does it cite the current literature?

Yes

If applicable, is the statistical analysis and its interpretation appropriate?

No

Are all the source data underlying the results available to ensure full reproducibility?

Yes

Are the conclusions drawn adequately supported by the results?

Partly

Are sufficient details of methods and analysis provided to allow replication by others?

Partly

Reviewer Expertise:

Hydrogen Production and Storage

I confirm that I have read this submission and believe that I have an appropriate level of expertise to confirm that it is of an acceptable scientific standard.

# Reviewer report: Reviewer response for version 1

**DOI:** 10.21956/openreseurope.22913.r60881

**Published:** 2025-11-03

**Authors:** Princewill M. Ikpeka

**Affiliations:** 1 Brunel University London, London, England, UK

**Overview**

 The paper is interesting, and the subject area is indeed very important to the energy storage/production discussion. However, I do not recommend indexing in its current form. It needs substantial revision in the following areas:

 - The introduction is quite long, which makes the research aim unclear. After reading through, I recommend synthesizing the sections: "Current state of the art of natural hydrogen" and "Previous research related to West Macedonia and neighbouring areas" into "Introduction" section rather than separate sections.

 - There is an issue with data reliability. The first two sampling campaigns did not yield reliable gas measurements due to sample preservation issues. This means that only the third and fourth campaigns provided usable gas data, which **greatly limits the evidence** available. 

 - The central hypothesis of the paper is not convincingly supported by the data presented. Measured hydrogen concentrations were extremely low, **only on the order of a few parts per million (ppm)** in the third campaign, and in the final (fourth) campaign **no hydrogen at all was detected**. For example, one water sample showed just 4.4 ppm H₂ (with thousands of ppm methane in comparison), and another had ~1.7 ppm H₂ – essentially trace levels. In the last round, the authors state “no hydrogen gas was registered”. Such minimal or absent H₂ results make it difficult to support the hypothesis of significant **natural hydrogen generation** in the area.

 - The authors propose that hydrogen might be getting consumed by microbes before detection (see Discussion), but without direct evidence (e.g. microbial analyses or consistent H₂ findings), this remains largely speculative. The data **do not provide clear proof** of natural hydrogen in these reservoirs, so the main premise of the study is left on uncertain footing.

 - In addition, the paper is largely exploratory and observational, and as such it does not employ statistical analyses like hypothesis testing, regression modelling, or p-value significance tests. This means the conclusions rely on expert judgment calls not on quantitative analysis.

Is the study design appropriate and does the work have academic merit?

Partly

Is the work clearly and accurately presented and does it cite the current literature?

Yes

If applicable, is the statistical analysis and its interpretation appropriate?

No

Are all the source data underlying the results available to ensure full reproducibility?

Yes

Are the conclusions drawn adequately supported by the results?

Partly

Are sufficient details of methods and analysis provided to allow replication by others?

Partly

Reviewer Expertise:

Hydrogen Production and Storage

I confirm that I have read this submission and believe that I have an appropriate level of expertise to confirm that it is of an acceptable scientific standard, however I have significant reservations, as outlined above.

# Response

**Published:** 2025-12-05

**Authors:** Pavlos Tyrologou

**Affiliations:** Geo-RΕsources, EΝergy and environmental management, CERTH, Athens, Greece

**Response to report 2** **Overview**

**The paper is interesting, and the subject area is indeed very important to the energy storage/production discussion. However, I do not recommend indexing in its current form. It needs substantial revision in the following areas:**

**Response: **Many thanks for your time, support and comments which are dully noted. We have now restructured and revised the manuscript. We have explicitly clarified that preservation issues affected the integrity of gas analyses from these campaigns. While part of the first sampling campaign was affected by preservation-related degassing, the subsequent datasets were collected under controlled conditions and show internally consistent geochemical patterns. Interpretations rely on multiple independent indicators (aqueous chemistry, geological context, spatial trends) that remain robust even considering potential underestimation in early gas values.  We ensure transparency regarding data quality and avoid overinterpretation. This revision strengthens the scientific credibility of the manuscript and aligns the study with accepted geochemical sampling standards.

**- The introduction is quite long, which makes the research aim unclear. After reading through, I recommend synthesizing the sections: "Current state of the art of natural hydrogen" and "Previous research related to West Macedonia and neighbouring areas" into "Introduction" section rather than separate sections.**

**Response: **Thank you for your suggestions. The authors acknowledge the reviewers comment and have shortened the introduction while also merged the subsections as it was pointed out

**- There is an issue with data reliability. The first two sampling campaigns did not yield reliable gas measurements due to sample preservation issues. This means that only the third and fourth campaigns provided usable gas data, which greatly limits the evidence available.**

**Response: **We thank the reviewer for this observation. We fully acknowledge that the first two sampling campaigns were affected by preservation issues. This was stated for transparency reasons but also to draw the attention to any future research on sample preservation and its importance. In the original and revised manuscript, we state that only the third and fourth campaigns yielded reliable helium measurement and confirmed hydrogen presence. Although this reduces the total number of usable gas datapoints, the third and fourth campaigns were conducted under controlled preservation conditions and show internally consistent compositions. Moreover, the geological context, aqueous geochemistry and spatial patterns provide independent lines of evidence that support the evaluation of subsurface processes and gas migration behaviour. The conclusions are therefore based on conservative, well-constrained data and remain scientifically robust within the defined scope of an exploratory study. The manuscript takes all these into account. While the available evidence is therefore more restricted, the dataset remains sufficient to evaluate geological processes and gas migration behaviour within the scope of an exploratory study.  

**- The central hypothesis of the paper is not convincingly supported by the data presented. Measured hydrogen concentrations were extremely low, only on the order of a few parts per million (ppm) in the third campaign, and in the final (fourth) campaign no hydrogen at all was detected. For example, one water sample showed just 4.4 ppm H₂ (with thousands of ppm methane in comparison), and another had ~1.7 ppm H₂ – essentially trace levels. In the last round, the authors state “no hydrogen gas was registered”. Such minimal or absent H₂ results make it difficult to support the hypothesis of significant natural hydrogen generation in the area.**  

**Response: **We acknowledge the reviewer’s point that measured hydrogen concentrations were low, ranging only from 1.7 to 4.4 ppm in the third campaign and falling below detection in the fourth campaign. Indeed, our revised text emphasises an evaluation of the geological potential for hydrogen generation and confinement within the Mesohellenic Basin. Thus, the revised manuscript distinguishes between (a) hydrogen generation mechanisms that may operate within the basin due to its mineralogical and tectonic characteristics, and (b) the detectable accumulation of hydrogen in measurable quantities. This distinction is now made explicit in the Introduction, Objectives, and Discussion. The low measured concentrations are acknowledged transparently. Our interpretation focuses instead on geological capacity, using measured trace concentrations to infer processes rather than volumes. This is consistent with the early exploratory nature of natural hydrogen research. Kindly also note that the research considers not only natural hydrogen but also helium which has been detected with analytical accuracy and confidence.

**- The authors propose that hydrogen might be getting consumed by microbes before detection (see Discussion), but without direct evidence (e.g. microbial analyses or consistent H₂ findings), this remains largely speculative.**

**Response: **We thank the reviewer for highlighting the need for careful interpretation regarding microbial consumption of hydrogen. In the revised manuscript, we present microbial consumption only as a possible explanation, supported by geochemical indicators such as reducing redox conditions, elevated Fe–Mn concentrations, and the presence of organic-rich strata in the Florina Basin. The text emphasises that this is a hypothesis grounded in analogous studies, not a confirmed mechanism for the sites investigated here. We state that future work involving microbial community characterisation would be required to confirm or refute this interpretation.

**The data do not provide clear proof of natural hydrogen in these reservoirs, so the main premise of the study is left on uncertain footing.**

**Response: **We appreciate the reviewer’s concern and agree that the measured hydrogen concentrations are low and do not constitute proof of substantial natural hydrogen accumulations. In the revised manuscript, we now explicitly state that the evidence reflects incipient or low-level generative processes rather than resource-grade hydrogen reservoirs. Our interpretation focuses on identifying geological conditions capable of producing hydrogen, such as serpentinisation, radiolysis, and Fe-bearing mineral alteration, and assessing whether these processes are consistent with the geochemical signals observed.  rather than asserting the presence of economically significant accumulations. Although hydrogen concentrations are modest, they form one component of a broader, multi-line evidence framework that includes geological context, mineralogical controls and aqueous geochemistry. The central theme of the study is to mostly evaluate geological potential. We have revised the text accordingly to further clarify the affroementioned.

**- In addition, the paper is largely exploratory and observational, and as such it does not employ statistical analyses like hypothesis testing, regression modelling, or p-value significance tests. This means the conclusions rely on expert judgment calls not on quantitative analysis.**

**Response: **Thank you again for this constructive comment. We agree that the study is primarily exploratory; however, we emphasise that all analytical procedures followed rigorous QA/QC protocols, including certified reference materials, duplicate measurements, instrument calibration with internationally traceable standards, and documented external reproducibility (0.2‰ for δ¹³C _CH₄_ and 2‰ for δD _CH₄_). These controls ensured that the geochemical patterns we report are robust even in the absence of formal hypothesis-testing statistics. Regarding statistical treatment, the dataset structure, characterised by small sample numbers per site and non-replicated measurements for several parameters, does not meet the assumptions for regression modelling or significance testing. Instead, we applied the only statistically defensible quantitative test available: an exploratory Spearman rank correlation between δ¹³C-CH₄ and δD-CH₄ for the four samples with complete isotopic data. The correlation coefficient was low (ρ ≈ 0.10), indicating no monotonic relationship between the two isotopic parameters. This outcome is consistent with our interpretation that the samples fall within a narrow biogenic isotopic range, with limited variability relative to analytical uncertainty. Importantly, the absence of strong statistical structure does not weaken the geological interpretation. Multiple independent lines of evidence, reproducible gas occurrences, stable isotope systematics, He-rich signatures, water chemistry and mapped fault–ultramafic contacts, converge toward a coherent conceptual model of subsurface gas generation. In this context, the repeated detection of hydrogen across campaigns suggests a genuine geological signal, even though concentrations lie near detection limits. The presence of ultramafic lithologies, reducing conditions, radiogenic helium and fault-controlled migration pathways is compatible with low-flux abiotic H₂ generation. Microbial consumption is also plausible and could explain the subdued surface values. As with the statistical limitations discussed above, confirming natural hydrogen systems requires expanded temporal and spatial monitoring, which we highlight as a clear direction for future work. This integrative method is standard practice in subsurface gas prospecting and is well suited to the reconnaissance phase of a developing geochemical system. Regarding hydrogen, the evidence is similarly robust within the constraints of an exploratory dataset. H₂ was detected repeatedly across several sites and campaigns, and its occurrence is spatially coherent with ultramafic lithologies, radiogenic helium anomalies and reducing subsurface conditions, factors that collectively support the plausibility of abiotic H₂ generation. At the same time, the low concentrations measured at the surface are consistent with microbial consumption, which is known to efficiently attenuate H₂ fluxes in shallow environments. Thus, while the existing dataset does not yet permit definitive source attribution, the combination of geochemical, isotopic and geological indicators strongly suggests that hydrogen generation processes may be active in West Macedonia. We trust that even without formal inferential statistics, the study provides a coherent, multi-line body of evidence:

1. High analytical precision and strict QA/QC confirm data reliability.
2. Temporal duplicate consistency supports the robustness of isotopic interpretations.
3. Isotope systematics (CH₄, He, water isotopes) align with recognised genetic pathways.
4. Geological context independently supports abiotic H₂ generation potential.

Future work will expand the dataset sufficiently to enable statistical modelling of spatial clustering, mixing processes and source–pathway relationships. At this early stage, however, the combination of geochemistry, isotopes and structural geology provides valid, defensible conclusions appropriate for an exploratory study.

# Reviewer report: Reviewer response for version 1

**DOI:** 10.21956/openreseurope.22913.r60886

**Published:** 2025-10-11

**Authors:** Nicolas Lefeuvre, Niko Adjie

**Affiliations:** 1 LFCR, Unversity of Pau and Pays de l'Adou, Pau, France; 2 Unversity of Pau and Pays de l'Adour, Pau, France

Review of Tyrologou et al., Manuscript

 General Assessment

 The manuscript presents an integrated study on gaseous systems (H₂, He, CH₄, and CO₂) in Western Macedonia, combining structural geology, sedimentology, and geochemistry. This multidisciplinary approach is a major strength of the paper, as it provides valuable insights into the mechanisms controlling gas generation, migration, and trapping. The authors provide an extensive new geochemical dataset (gas chromatography, inductively coupled plasma, and isotope analyses) from both water and gas samples collected at multiple hydrothermal sources, which constitutes a significant contribution to the understanding of natural hydrogen systems.

 However, despite the richness of the data, the manuscript lacks clarity and focus. The research question and logical thread of the paper are often difficult to follow due to structural issues, redundancies, and digressions into socio-economic and political topics that are not directly relevant to a scientific article. The paper would benefit from a thorough reorganization following a classical structure (Introduction, Geological Context, Methods, Results, Discussion, and Conclusion). The clarity, conciseness, and scientific rigor of each section should be enhanced to help the reader clearly understand the problem, the methodology, and the conclusions.

 Major Comments

 1. Introduction

 The Introduction is overly long and fragmented, which prevents a clear understanding of the main research question. It should be condensed into a coherent scientific narrative that succinctly states the problem, its significance, and the context of natural hydrogen. Political and environmental aspects should be summarized in two or three lines at most.

 The manuscript would benefit from a clearer definition of its scientific novelty. While it compiles valuable field and isotopic data, the research question remains vague. The authors should explicitly state their hypothesis and objectives at the end of the Introduction to help readers understand the study’s purpose.

 Some statements are unclear or inaccurate; for example, 'Natural hydrogen mechanisms are still under development' should be rephrased as 'The understanding of natural hydrogen generation mechanisms is still under investigation.' The section 'Current State of the Art' lacks a detailed discussion of hydrogen production mechanisms and diverges into unrelated topics. It should focus on generation processes relevant to the study area and cite up-to-date references.

 2. Geological Context

 The geological description is detailed and thorough, but overly extensive. Many details are not directly relevant to the research question and tend to obscure the main message. The section should focus on key aspects such as source rocks, basin thickness, structural controls (faults) on gas migration, and potential reservoir rocks.

 The authors should merge 'Previous research related to West Macedonia and neighbouring areas' with 'Geological setting' to avoid repetition. Maintain a logical flow—start with regional geology, then focus on specific study zones relevant to the presented data. Clarify whether the study concerns West Macedonia or Greece, as this point remains ambiguous.

 Figures 1–5 include excessive descriptive geological information. Simplifying these or adding a conceptual schematic linking tectonic setting, source rocks (ophiolites, lignites, carbonates), and migration pathways would greatly improve readability.

 3. Materials and Methods

 The methodology is comprehensive but overly detailed. Only essential information should be provided—sample types, volumes, sampling conditions, and analytical tools. Avoid unnecessary procedural details such as ‘The values of the latter were recorded in field books or digital devices.’ Figures 6–8 showing sampling steps could be moved to Supplementary Material.

 Simplify over-detailed methodological descriptions and remove non-scientific instructions (e.g., 'practice a lot before commencing the sampling process,' 'check under daylight for bubbles'). Provide details relevant to the analytical precision, such as GC column type, detection limits, and isotopic accuracy.

 4. Results

 The Results section is rich but should focus solely on presenting data without interpretation. Interpretations belong in the Discussion. Values should be expressed as ranges with appropriate units, and isotopic notation must follow standard conventions (δ¹³C–CH₄, δD–CH₄, δD–H₂, δD–H₂O, δ¹⁸O–H₂O).

 The link between data and interpretation should be strengthened. Rather than listing numerous values, summarize and highlight key geochemical and isotopic trends (e.g., δD–δ¹⁸O correlations, spatial variations in CH₄/H₂/He) and discuss their geological implications.

 Remove irrelevant details such as water potability discussions. Ensure tables contain measurement units. Clarify the purpose of blind samples—specify whether they served as analytical blanks or matrix controls—and verify the consistency of repeated samples (e.g., 'Katakali-2' and 'Kivotos-2').

 5. Discussion

 The Discussion lacks structure and clarity. It should be reorganized into subsections addressing specific scientific questions such as gas sources, migration pathways, trapping mechanisms, and implications. The discussion should be hypothesis-driven, directly linking interpretations to results.

 Currently, some parts of the Discussion are embedded in the Results section. These should be consolidated. Address the noted inconsistency: the manuscript claims that 'low silica enhances H₂ generation' but also that 'low silica can be indicated by magnetite.' In ultramafic contexts, magnetite is an indicator of serpentinization and thus hydrogen generation. Clarify this contradiction.

 6. Minor, Technical, and Specific Comments

 6.1 General Technical Corrections

 - Ensure isotopic notation (δ¹³C–CH₄, δD–H₂O, δ¹⁸O–H₂O) is consistently formatted using correct Greek symbols and superscripts (use 'O' not zero).

 - Verify typographic consistency for isotopic symbols (δ¹³C, δD, δ¹⁸O).

 - Include measurement units consistently across all tables, particularly for δ-values and gas concentrations.

 - Correct typographical errors such as '1..22' in Table 5 (double dot under dissolved O₂).

 - Condense figures and tables to highlight only essential data; move over-descriptive figures to Supplementary Material.

 - Clarify the expression 'public indications' (Page 10).

 - Use consistent terminology: distinguish between 'potential gas-bearing formations' and 'storage reservoirs.'

 - Replace outdated or irrelevant references with more recent sources.

 - Rephrase ambiguous or awkward sentences for clarity, e.g., 'Hydrogen is highly reactive… preservation throughout migration is a risk' → 'Hydrogen is highly reactive and susceptible to redox conditions, making long-term preservation during migration challenging.'

 - Rephrase 'This is also making sure…' → 'This is also to make sure…'

 - Replace 'deploying natural hydrogen in the EU' with 'establishing a natural hydrogen economy in the EU.'

 - Figures 6–8 show overly detailed sampling equipment. Consider moving these to Supplementary Material to streamline the main text.

 - Clarify whether 'Katakali-2' and 'Kivotos-2' samples are duplicates or replicate analyses.

 - Explain the rationale for using 'blind samples'—was bottled water used as an analytical blank or matrix control?

 6.2 Page-Specific Remarks

 Page 4, paragraph 5: Rephrase 'Natural hydrogen mechanisms…' as 'The understanding of natural hydrogen generation mechanisms is still under development.'

 Page 4, paragraph 6: Adjust preservation and EU hydrogen economy phrasing as above.

 Page 5, paragraph 6: Clarify the contradiction regarding silica content and H₂ generation mechanisms.

 Page 10, paragraph 4: Differentiate between CO₂ storage and natural H₂ leakage—surface leaks may indicate deeper accumulations.

 Page 10, paragraph 4: Clarify what is meant by 'public indications.'

 Page 22, paragraph 2: Discuss the robustness of measurements given the small He/H₂ concentrations and analytical uncertainty.

 Tables 2–5: Ensure δD–H₂O uses the letter O (not zero).

 Overall Conclusion

 Overall, the study provides valuable geochemical and isotopic data from a poorly documented region. However, its scientific impact would increase significantly if the manuscript were restructured around its core research questions, with redundant methodological details condensed. A stronger integration of geological, hydrogeochemical, and isotopic evidence would result in a clearer conceptual model of gas generation and migration in West Macedonia.

Is the study design appropriate and does the work have academic merit?

Partly

Is the work clearly and accurately presented and does it cite the current literature?

No

If applicable, is the statistical analysis and its interpretation appropriate?

I cannot comment. A qualified statistician is required.

Are all the source data underlying the results available to ensure full reproducibility?

Yes

Are the conclusions drawn adequately supported by the results?

Partly

Are sufficient details of methods and analysis provided to allow replication by others?

Yes

Reviewer Expertise:

Geochimie et production de l’hydrogène naturel, migration et exploration.

We confirm that we have read this submission and believe that we have an appropriate level of expertise to state that we do not consider it to be of an acceptable scientific standard, for reasons outlined above.

# Response

**Published:** 2025-12-05

**Authors:** Pavlos Tyrologou

**Affiliations:** Geo-RΕsources, EΝergy and environmental management, CERTH, Athens, Greece

**Response to report 1** Review of Tyrologou et al., Manuscript

 General Assessment

**The manuscript presents an integrated study on gaseous systems (H₂, He, CH₄, and CO₂) in Western Macedonia, combining structural geology, sedimentology, and geochemistry. This multidisciplinary approach is a major strength of the paper, as it provides valuable insights into the mechanisms controlling gas generation, migration, and trapping. The authors provide an extensive new geochemical dataset (gas chromatography, inductively coupled plasma, and isotope analyses) from both water and gas samples collected at multiple hydrothermal sources, which constitutes a significant contribution to the understanding of natural hydrogen systems.**

**Response:** We thank the reviewers for their comments and feedback on the scientific concept of the manuscript. The authors truly appreciate the devoted volunteer time of the reviewer to support the rigorous process of scientific correction.  

**However, despite the richness of the data, the manuscript lacks clarity and focus. The research question and logical thread of the paper are often difficult to follow due to structural issues, redundancies, and digressions into socio-economic and political topics that are not directly relevant to a scientific article. The paper would benefit from a thorough reorganization following a classical structure (Introduction, Geological Context, Methods, Results, Discussion, and Conclusion). The clarity, conciseness, and scientific rigor of each section should be enhanced to help the reader clearly understand the problem, the methodology, and the conclusions.**  

**Response: **We thank the reviewers for their comments. The manuscript has now restructured along the suggested structure – Introduction, Geological Context, Methods, Results, Discussion and Conclusion.

**Major Comments**

**1. Introduction**

**The Introduction is overly long and fragmented, which prevents a clear understanding of the main research question. It should be condensed into a coherent scientific narrative that succinctly states the problem, its significance, and the context of natural hydrogen. Political and environmental aspects should be summarized in two or three lines at most.**

**The manuscript would benefit from a clearer definition of its scientific novelty. While it compiles valuable field and isotopic data, the research question remains vague. The authors should explicitly state their hypothesis and objectives at the end of the Introduction to help readers understand the study’s purpose.**

**Some statements are unclear or inaccurate; for example, 'Natural hydrogen mechanisms are still under development' should be rephrased as 'The understanding of natural hydrogen generation mechanisms is still under investigation.' The section 'Current State of the Art' lacks a detailed discussion of hydrogen production mechanisms and diverges into unrelated topics. It should focus on generation processes relevant to the study area and cite up-to-date references.**

**Response: **Many thanks for your comments. The introductions has now been simplified and significantly shortened about political and environmental aspects to three lines . The introduction and current state of the art of natural hydrogen have merged into one. 'Natural hydrogen mechanisms are still under development' was rephrased as 'The understanding of natural hydrogen generation mechanisms is still under investigation.' A hypothesis and objectives have been explicitly stated together with the objectives. On the interest of saving space and keeping introduction short as requested by the reviewers, only the mechanisms of natural hydrogen are provided with the relevant references to the reader’s interest. Providing more detail on the natural mechanisms for hydrogen will render the manuscript onto the review domain. The references provided have been updated and include additional newest ones where it makes sense, however the original ones kept as are still exceptionally valid.

**2. Geological Context**

**The geological description is detailed and thorough, but overly extensive. Many details are not directly relevant to the research question and tend to obscure the main message. The section should focus on key aspects such as source rocks, basin thickness, structural controls (faults) on gas migration, and potential reservoir rocks.**

**The authors should merge 'Previous research related to West Macedonia and neighbouring areas' with 'Geological setting' to avoid repetition. Maintain a logical flow—start with regional geology, then focus on specific study zones relevant to the presented data.**

**Response: **We thank the reviewers for their comments. The sections now have been merged, and repetitions have been identified and deleted accordingly. The sections are now restructured to reflect the flow from supra regional to regional to local geology based on the reviewer’s recommendations. **Clarify whether the study concerns West Macedonia or Greece, as this point remains ambiguous.** **Response: **We thank the reviewers for their comments. A specific phrase has been added in the hypothesis section in the introduction clarifying that West Macedonia is the focus of the study.

**Figures 1–5 include excessive descriptive geological information. Simplifying these or adding a conceptual schematic linking tectonic setting, source rocks (ophiolites, lignites, carbonates), and migration pathways would greatly improve readability.** **Response: **Many thanks for your comment. Figure 1 and 3 have been deleted; a conceptual schematic is provided in figure 9 with the remaining figures **3. Materials and Methods**

**The methodology is comprehensive but overly detailed. Only essential information should be provided—sample types, volumes, sampling conditions, and analytical tools. Avoid unnecessary procedural details such as ‘The values of the latter were recorded in field books or digital devices.’** **Response: **Many thanks for your comment. The section has been cleaned of overly detailed procedural details, while ensuring that methods are described in sufficient detail for others to repeat the research in accordance with the Open Research Europe requirements. **Figures 6–8 showing sampling steps could be moved to Supplementary Material.**

**Simplify over-detailed methodological descriptions and remove non-scientific instructions (e.g., 'practice a lot before commencing the sampling process,' 'check under daylight for bubbles'). Provide details relevant to the analytical precision, such as GC column type, detection limits, and isotopic accuracy.**

**Response: **Many thanks for your comments. The section has been cleaned of over-detailed procedural details, including non-scientific instructions. GC column type and detection limits for the gas analysis were added. We have kept Figures 6-8 to provide a reference for future readers who want to reproduce the methodology. We have provided and clarified the type of GC column, gas analysis detection limits and description of the isotopic precision (reproducibility) and accuracy (treacability) for all stable isotope measurements quantifying precision via one standard deviation values and ensuring accuracy through normalisation against international standards of SMOW, VSMOW2 and SLAP2 V-PDB. The analysis for He isotopes is also quantified, with its total analytical error, encompassing both precision and accuracy, stated as generally less than 1%. We understand isotopic accuracy on the basis of  which international standard was used and which Certified Reference Materials (CRMs) were used for calibration with a statement on the final quantified uncertainty (± value) on the reported δ values. Regarding gas detection limits for H2 we have modified the text in various places accordingly.

**4. Results**

**The Results section is rich but should focus solely on presenting data without interpretation. Interpretations belong in the Discussion. Values should be expressed as ranges with appropriate units, and isotopic notation must follow standard conventions (δ¹³C–CH₄, δD–CH₄, δD–H₂, δD–H₂O, δ¹⁸O–H₂O).**

**Response: **We thank the reviewers for their comments **. **We agree that, according to conventional scientific structure, interpretations should be presented primarily in the Discussion. However, given the complexity of the geochemical and isotopic datasets, we retained minimal and factual commentary within the results section to help guide the reader and enhance clarity. This includes brief contextualisation but avoids extensive interpretation or mechanism discussion. Nevertheless, we have reviewed the section again and relocated more detailed interpretations to the Discussion, in line with the reviewer’s suggestion, while preserving clarity and narrative flow. Isotopic notations follow standard conventions  (δ¹³C _CH₄_, δD _CH₄_, δD _H₂_, δD _H₂O_, δ¹⁸O _H₂O_) including Figure 9.

**The link between data and interpretation should be strengthened. Rather than listing numerous values, summarize and highlight key geochemical and isotopic trends (e.g., δD–δ¹⁸O correlations, spatial variations in CH₄/H₂/He) and discuss their geological implications.**

**Response: **We thank the reviewer for their comments. A methane genetic diagram has been added to visualise results for Katakali and Kivotos.

**Remove irrelevant details such as water potability discussions. Ensure tables contain measurement units. Clarify the purpose of blind samples—specify whether they served as analytical blanks or matrix controls—and verify the consistency of repeated samples (e.g., 'Katakali-2' and 'Kivotos-2').**

**Response: **Many thanks for your comments. Certain details have been omitted to facilitate further the flow of the text. Text has been added to clarify the purpose of blind samples. Tables have been improved and contain measurement units. We clarify that 'Katakali-2' and 'Kivotos-2' are not analytical duplicates but independent field duplicates, i.e., separately collected samples from the same spring location. These were analysed in parallel to assess site-level variability. δ¹³C _CH₄_ values showed good agreement for Katakali (–69.5‰ vs –69.8‰), but δ²H _CH₄_ differed by ~21‰. Kivotos showed greater variability (6.0‰ in δ¹³C _CH₄_ and 15.0‰ in δ²H _CH₄_), likely reflecting local heterogeneity rather than analytical error. A clarifying paragraph has been added before Table 8.

 5. Discussion

**The Discussion lacks structure and clarity. It should be reorganized into subsections addressing specific scientific questions such as gas sources, migration pathways, trapping mechanisms, and implications. The discussion should be hypothesis-driven, directly linking interpretations to results.**

**Response: **We duly noted the reviewer's comments. These made us aware of the confusion, and we have now restructured the discussion section based on the scientific hypothesis, combined with the structure pointed out by the reviewer.

**Currently, some parts of the Discussion are embedded in the Results section. These should be consolidated.**

**Response: **Many thanks for your comment. Please see our answer above. The results section has been reorganised. Some minor interpretative text remains to facilitate the argument development as well as to help guide the reader and enhance clarity.

**Address the noted inconsistency: the manuscript claims that 'low silica enhances H₂ generation' but also that 'low silica can be indicated by magnetite.'  In ultramafic contexts, magnetite is an indicator of serpentinization and thus hydrogen generation. Clarify this contradiction.**

**Response: **We thank the reviewer for their comments and support in the rigorous scientific peer-review correction process. We acknowledge that the sentence structure is confusing and have improved it for clarity and corrected it accordingly to reflect the proper H _2_ generation conditions. The section has also been enhanced with the addition of scientific references.

 6. Minor, Technical, and Specific Comments

 6.1 General Technical Corrections

**- Ensure isotopic notation (δ¹³C–CH₄, δD–H₂O, δ¹⁸O–H₂O) is consistently formatted using correct Greek symbols and superscripts (use 'O' not zero).** **Response: **Amended where needed

**- Verify typographic consistency for isotopic symbols (δ¹³C, δD, δ¹⁸O).** **Response: **Amended where needed **- Include measurement units consistently across all tables, particularly for δ-values and gas concentrations.** **Response: **Amended where needed **- Correct typographical errors such as '1..22' in Table 5 (double dot under dissolved O₂).**

**Response: **Corrected

**Condense figures and tables to highlight only essential data; move over-descriptive figures to Supplementary Material.**

**Response: **Many thanks for your comments. We believe that the current figures are essential for guiding the reader through the key geochemical and isotopic interpretations presented in the main text. They provide critical visual support for understanding the spatial and isotopic relationships discussed, and removing them may hinder the clarity and coherence of the narrative.

**Clarify the expression 'public indications' (Page 10).**

**Response: **Phrase was omitted as it provides the same meaning as the b) anecdotal data **-**

**Use consistent terminology: distinguish between 'potential gas-bearing formations' and 'storage reservoirs.'**

**Response: **Many thanks for your comments. We acknowledge the difference between the engineered or naturally suitable formations to inject and store gas such as CO _2_ and the geological formations that naturally generate or contain gas. The ambiguity arose as the text described how the research evolved from the Pilot Strategy, a carbon capture and storage project. The ambiguity is resolved with the reorganisation of the text and the provision of a definition in the introductory section

 - **Replace outdated or irrelevant references with more recent sources.**

**Response: **Thanks for your comments. We acknowledge the importance of incorporating recent literature and have reviewed the reference list accordingly. However, several of the cited long established sources remain foundational in the field and continue to be widely referenced by recent publications. Their inclusion ensures a robust conceptual framework and historical context, particularly where newer studies build directly upon or reaffirm these earlier works. Where appropriate, we have also cross-checked and added complementary recent references to demonstrate continuity and relevance with current scientific understanding. -

**Rephrase ambiguous or awkward sentences for clarity, e.g., 'Hydrogen is highly reactive… preservation throughout migration is a risk' → 'Hydrogen is highly reactive and susceptible to redox conditions, making long-term preservation during migration challenging.'**

**Response: **The authors are obliged by the constructive support of the reviewer and his thorough reading of the manuscript. We have rephrased as it was pointed out.

**- Rephrase 'This is also making sure…' → 'This is also to make sure…'**

**Response: **Corrected

**- Replace 'deploying natural hydrogen in the EU' with 'establishing a natural hydrogen economy in the EU.'**

**Response: **Many thanks for your suggestion, the phrase was deleted as part of the section shortening.

 - **Figures 6–8 show overly detailed sampling equipment. Consider moving these to Supplementary Material to streamline the main text.**

**Response: **Many thanks for your comments and we appreciate the reviewer’s suggestion to streamline the manuscript by relocating Figures 6–8 to the Supplementary Material. We respectfully believe that retaining these figures in the main body adds value by clearly illustrating the field methodology, which is essential for transparency and reproducibility, even if this sampling equipment is standardised. These visuals also enhance accessibility for readers who are less familiar with the equipment or protocols used in gas and water sampling.

**- Clarify whether 'Katakali-2' and 'Kivotos-2' samples are duplicates or replicate analyses. Explain the rationale for using 'blind samples'—was bottled water used as an analytical blank or matrix control?**

**Response: **Many thanks for your comments. The question remark has been clarified in the text and has also been answered in an aforementioned remark. We used two independent samples per site, taken minutes apart, to assess spatial or micro-scale variability within the same system. These are not laboratory duplicates or analytical replicates. An explanatory text has been added in the manuscript.

**6.2 Page-Specific Remarks**

**Page 4, paragraph 5: Rephrase 'Natural hydrogen mechanisms…' as 'The understanding of natural hydrogen generation mechanisms is still under development.'**

**Response: **Many thanks for your comments., text has been ammended

**Page 4, paragraph 6: Adjust preservation and EU hydrogen economy phrasing as above.**

**Response: **We thank the reviewer for their comments , the phrase was deleted as part of the section shortening and restructuring.

**Page 5, paragraph 6: Clarify the contradiction regarding silica content and H₂ generation mechanisms.**

**Response: **We acknowledge that the sentence structure is confusing and have improved it for clarity and corrected it accordingly to reflect the proper H2 generation conditions. The section has also been enhanced with the addition of scientific references.

**Page 10, paragraph 4: Differentiate between CO₂ storage and natural H₂ leakage—surface leaks may indicate deeper accumulations.**

**Response:** We thank the reviewer for their comment, which has strengthened the manuscript's scientific credibility. We have differentiated between CO _2_ storage and hydrogen mobility based on their solubility properties. Text has been added in the corresponding paragraph for further clarification.

**Page 10, paragraph 4: Clarify what is meant by 'public indications.'**

**Response: **Phrase was omitted as it provides the same meaning as the b) anecdotal data

**Page 22, paragraph 2: Discuss the robustness of measurements given the small He/H₂ concentrations and analytical uncertainty.**

**Response: We** thank the reviewer for this important observation. Hydrogen and helium were analysed by gas chromatography (Perkin Elmer Clarus 500 equipped with a double Carboxen 1000 packed column system and TCD–FID detectors), using Ar as the carrier gas. The TCD detection limit for H₂ and He is 1–10 μmol/mol (ppmv). The H _2_ values reported are near the lower end of this range, and we acknowledge that such low concentrations require cautious interpretation. We still believe that the H₂ hold value since they were reproducibly detected at the same locations across multiple campaigns apart from us and show spatial coherence with ultramafic lithologies, major fault systems, and independent geochemical indicators such as alkaline pH, elevated Li–B, and reducing Fe–Mn conditions. We have added a new paragraph in the Discussion clarifying these points and acknowledging the limitations while demonstrating why the H₂ signals remain valid for interpretation. We appreciate the reviewer’s comment, which has improved the transparency of our dataset interpretation.  

**Tables 2–5: Ensure δD–H₂O uses the letter O (not zero).**

**Response: **Ammended  

**Overall Conclusion**

**Overall, the study provides valuable geochemical and isotopic data from a poorly documented region. However, its scientific impact would increase significantly if the manuscript were restructured around its core research questions, with redundant methodological details condensed. A stronger integration of geological, hydrogeochemical, and isotopic evidence would result in a clearer conceptual model of gas generation and migration in West Macedonia.**  

**Response: **We thank the reviewer for the constructive feedback and his commitment to the scientific review process. The manuscript was revised and restructured accordingly.  

**Is the work clearly and accurately presented and does it cite the current literature?** No – **Response: **Thanks for your comments and we partially agree on the work that needs enhancement and needs to be clearer as it was described previously. The manuscript benefits from a very comprehensive literature review, we have included more references and we remain open to any additional literature that we may have missed by not being aware. Foundational sources are retained because they remain central in the current scientific literature, and more recent studies are built directly upon them. While every effort has been made to engage with the most relevant and up-to-date scientific literature, it is important to acknowledge a structural limitation inherent to the current scholarly landscape. A significant proportion of research outputs remains behind paywalls, as many authors publish in subscription-based journals. Access to these works is therefore restricted and not uniformly available across institutions or research teams, creating an unavoidable disparity in information accessibility. This manuscript reflects the fullest integration of all literature that was accessible through institutional subscriptions, open-access platforms, and publicly available databases at the time of writing. The authors strongly support the principles of open science, as unrestricted access to scientific publications and data is essential for ensuring equitable participation in research, fostering transparency, and accelerating scientific progress.
